# A geminal antimony(iii)/phosphorus(iii) frustrated Lewis pair†

**DOI:** 10.1039/d4sc02785j

**Published:** 2024-07-01

**Authors:** Jonas Krieft, Pia C. Trapp, Yury V. Vishnevskiy, Beate Neumann, Hans-Georg Stammler, Jan-Hendrik Lamm, Norbert W. Mitzel

**Affiliations:** a Chair of Inorganic and Structural Chemistry, Center for Molecular Materials CM_2_, Faculty of Chemistry, Bielefeld University, Universitätsstraße 25 Bielefeld 33615 Germany mitzel@uni-bielefeld.de

## Abstract

The geminal Lewis pair (F_5_C_2_)_2_SbCH_2_P(*t*Bu)_2_ (1) was prepared by reacting (F_5_C_2_)_2_SbCl with LiCH_2_P(*t*Bu)_2_. Despite its extremely electronegative pentafluoroethyl substituents, the neutral 1 exhibits a relatively soft acidic antimony function according to the HSAB concept (hard–soft acid–base). These properties lead to a reversibility in the binding of CS_2_ to 1, as observed by VT-NMR spectroscopy, while no reaction with CO_2_ is observed. The reaction behaviour towards heterocumulenes and the specific interaction situation in the CS_2_ adduct were analysed by quantum chemical calculations. The FLP-type reactivity of 1 has also been demonstrated by reaction with a variety of small molecules (SO_2_, PhNCO, PhNCS, (MePh_2_P)AuCl). The reactions of 1 with PhNCO and PhNCS led to different types of cyclic addition products: PhNCO adds with its N=C bond and PhNCS adds preferentially with its C=S bond. The reaction of 1 with (MePh_2_P)AuCl gave an adduct {[(F_5_C_2_)_2_SbCH_2_(*t*Bu)_2_P]_2_Au}^+^ with a clamp-like structure binding a chloride anion by its two antimony atoms in chelate mode. Compound 1 and its adducts have been characterised by X-ray diffraction experiments, multinuclear NMR spectroscopy, elemental analyses and computational calculations (DFT, QTAIM, IQA).

## Abstract 2

(F_5_C_2_)_2_SbCH_2_P(*t*Bu)_2_ reacts in a FLP-like manner with CS_2_, SO_2_, PhNCO/S and (MePh_2_P)AuCl. The CS_2_ and AuCl adduct show secondary interactions between the Lewis acidic Sb atom and electron rich atoms, intra- and intermolecular, respectively.

## Graphical abstract

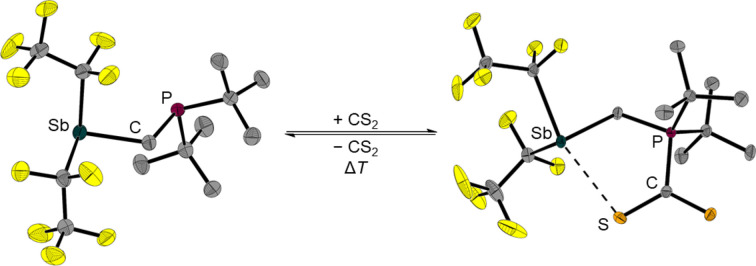

## Introduction

Since the pioneering work of Stephan and Erker in the field of Frustrated Lewis Pairs (FLP), this part of modern main group chemistry has developed rapidly and in many directions.^1^ Steric shielding and ring strain can prevent the formation of a stable adduct between Lewis acid and Lewis base sites and thus the neutralisation of the two functions within one molecule. The unused reaction potential can now be used to activate various small molecules or to catalyse reactions.^2–5^ The diversity of combinations of Lewis acids and bases in FLP systems continues to grow, but the “typical” combinations of Lewis acids of the third main group (B, Al) and Lewis bases of the fifth main group (N, P) dominate.^6,7^ The use of pnictogens in the base functions is established, but the elements of this main group can also have interesting Lewis acidic properties, making them very interesting and variable building blocks.^8^ At least since Olah's work on the so-called magic acid, antimony compounds have become an indispensable part of Lewis acid chemistry.^9,10^ Several contributions of Gabbaï and co-workers have shown that Lewis acidic stibonium ions and various stiboranes are not only able to activate C–F bonds,^11^ but also to trap halide ions^12^ and to act as ligands for transition metal complexes.^13^ Antimony has a special position in this respect due to its most pronounced Lewis acidity within the group.^14^ Despite Sb(iii) atoms have a free pair of electrons, their Lewis acidity can be reinforced by introducing perfluorinated substituents.^10,15^ In this way, a distinct σ-hole can be induced on the formally Lewis basic Sb(iii) atom. The acidity of this σ-hole depends on the electron-withdrawing properties of the spatially opposite substituent, making it an interesting and flexible building block for the synthesis of functionalised Lewis acids and FLP systems.^16^

We have recently reported a bidentate and a tetradentate Sb(iii)-based poly-Lewis acid capable of chelate-binding halide ions, dimethyl chalcogenides and nitrogen heterocycles by pnictogen bonding.^17,18^ We have also exploited the special properties of the Sb(C_2_F_5_)_2_ moiety to develop the new neutral geminal FLP presented here. In terms of Pearson's HSAB concept,^19^ the relatively soft Lewis acid Sb(iii) of this unit should be favourable for reversible reactions. Similarly, we have previously introduced the Sn/P-FLP (F_5_C_2_)_3_SnCH_2_P(*t*Bu)_2_, which reversibly binds CO_2_ while forming a stable adduct with the softer CS_2_. (F_5_C_2_)_3_SnCH_2_P(*t*Bu)_2_ also reacts with a variety of small molecules and stabilises highly reactive species, including the elusive sulphur monoxide.^5,20,21^

## Results and discussion

**Scheme 1:**
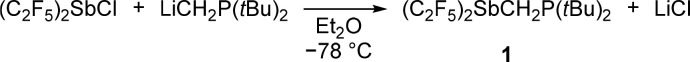
Synthesis of FLP 1 from (F_5_C_2_)_2_SbCl and LiCH_2_P(*t*Bu)_2_.

Starting from (F_5_C_2_)_2_SbCl,^17^ the intramolecular FLP (F_5_C_2_)_2_SbCH_2_P(*t*Bu)_2_ (1) was prepared by reaction with LiCH_2_P(*t*Bu)_2_ ^22^ in a nucleophilic substitution (Scheme 1).

**Fig. 1:**
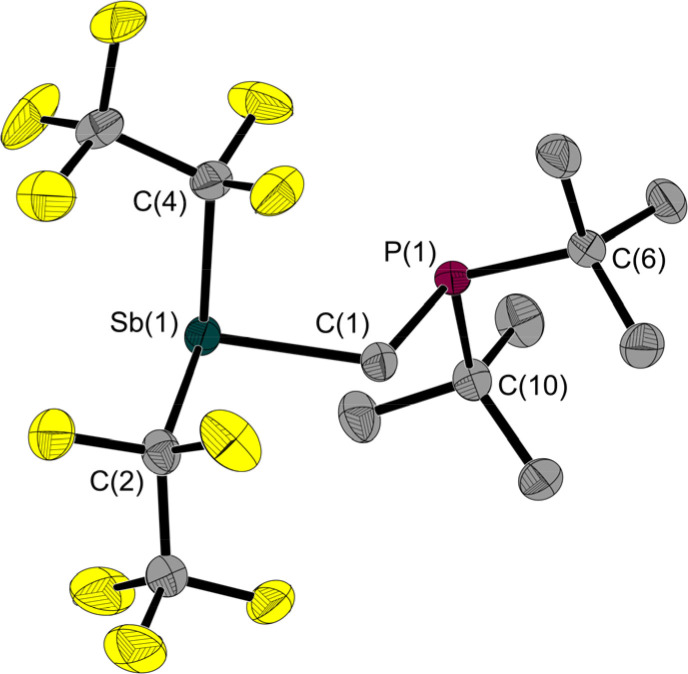
Molecular structure of 1 in the solid state. Ellipsoids are set at 50% probability; hydrogen atoms and minor occupied disordered atoms are omitted for clarity. Selected interatomic distances [Å] and angles [°]: Sb(1)–C(1) 2.157(1), Sb(1)–C(2) 2.238(1), Sb(1)–C(4) 2.217(2), P(1)–C(1) 1.860(1), P(1)–C(6) 1.891(1), P(1)–C(10) 1.886(1), Sb(1)⋯P(1) 3.306(1); C(1)–Sb(1)–C(2) 93.2(1), C(1)–Sb(1)–C(4) 91.3(1), C(4)–Sb(1)–C(2) 95.8(1), C(1)–P(1)–C(6) 100.2(1), C(1)–P(1)–C(10) 101.6(1), C(10)–P(1)–C(6) 111.3(1), P(1)–C(1)–Sb(1) 110.6(1).

Since 1 is a liquid under normal conditions, a single crystal suitable for X-ray diffraction was grown by *in situ* crystallisation at 283.9 K on the diffractometer. After the formation of a tiny seed crystal, the sample was cooled to 100 K. The molecular structure (Fig. 1) shows a distance between the Sb and P atoms in 1 of 3.306(1) Å, much longer than the sum of the covalent radii indicating that, at most, only a weakly stabilising interaction exists.^23^ The Sb⋯P distance is between geminal atoms and thus by its nature less than the sum of the van der Waals radii. Therefore, the Sb–C–P angle is more telling about a possible attractive Sb⋯P interaction. At 110.6(1)° it is smaller than the corresponding Sn–C–P angle in the Sn/P-FLP mentioned above with 113.9(1)°.^5^ However, the Sb–C–P angle of the rigid methylene backbone is too obtuse for the formation of an intramolecular Lewis acid/base adduct, as was also observed in all (F_5_C_2_)_*n*_ECH_2_P(*t*Bu)_2_ FLPs we have presented so far.^3–5^ The Sb atom is trigonal-pyramidal coordinated, as is common for trisubstituted pnictogen atoms in the oxidation state +III. Expectedly, the angles including the Sb position are between 91.3(1) and 95.8(1)°, *i.e.* close to 90°.^24^

The ^31^P NMR resonance of 1 at 15.5 ppm is in the typical range for methylene-bridged Lewis acid/P(*t*Bu)_2_ systems (*e.g. δ*(^31^P) (F_5_C_2_)_3_SnCH_2_P(*t*Bu)_2_ 17.2 ppm,^5^ (F_5_C_2_)_3_SiCH_2_P(*t*Bu)_2_ 18.5 ppm ^4^). Also typical for these systems are the ^13^C{^1^H} NMR resonances of 1 for the methylene carbon atoms at 9.0 ppm and the ^19^F NMR resonances of the pentafluoroethyl groups at −82.7 and −110.5/−111.1 ppm.^4,5^

We performed Lewis acidity tests by the Gutmann–Beckett method^25^ with OPEt_3_ and the modified method for soft Lewis acids with SePMe_3_ presented by Lichtenberg.^26^ After addition of OPEt_3_ to 1, we did not see any variation in the chemical shifts of 1 and OPEt_3_. We assume that the antimony-oxygen interaction is too unfavourable. With the softer Lewis base SePMe_3_, we observed a selenium transfer from SePMe_3_ to 1 to give (F_5_C_2_)_2_SbCH_2_(Se)P(*t*Bu)_2_ and PMe_3_.

Since these experimental Lewis acidity tests did not give a conclusive answer, we calculated the fluorine ion affinity (FIA) of 1 using a method by Greb *et al.* with an FIA of 278 kJ mol^−1^ it is well comparable to AsCl_3_ (276 kJ mol^−1^) and is below, for example, SbF_3_ (290 kJ mol^−1^), SbCl_3_ (309 kJ mol^−1^) and Sb(C_2_F_5_)_3_ (315 kJ mol^−1^).^15^

**Fig. 2:**
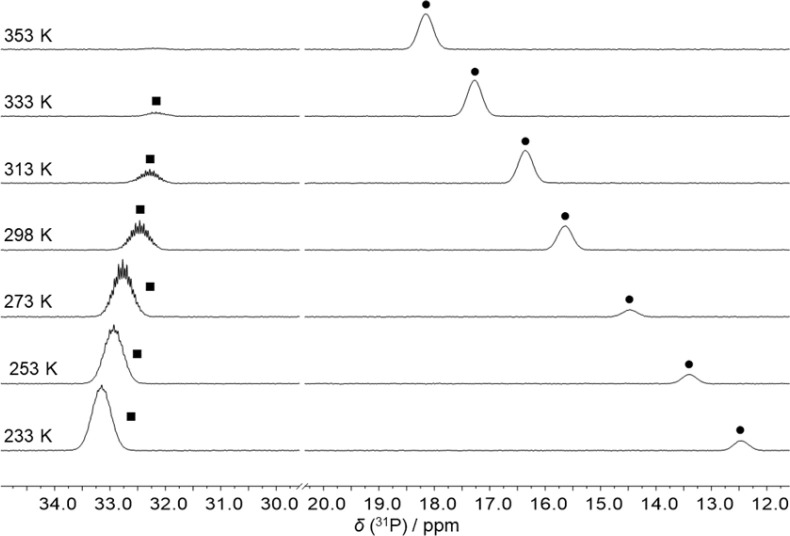
^31^P NMR spectra of a sample of a mixture of 1 and CS_2_ at different temperatures. The peaks of the FLP 1 (●) and of the CS_2_ adduct 2 (■) are labelled.

The reaction of FLP 1 with CO_2_ gave no detectable adduct. In contrast, the reaction with CS_2_ resulted in a temperature-dependent equilibrium, as observed by VT-NMR spectroscopy (Fig. 2). At room temperature, there is an equilibrium between the adduct 2 and the free FLP 1 plus free CS_2_ in approximately equal proportions. After cooling the solution to 233 K, the adduct is dominant in the solution and only 10% of the free FLP remains unbound. Cooling the solution shifts the resonance of 1 (*δ*(^31^P) at 298 K: 15.6 ppm) towards high field, while the multiplet of 2 (*δ*(^31^P) at 298 K: 32.5 ppm) is low-field shifted.

This experimentally observed behaviour is confirmed by the results of Density Functional Theory (DFT) calculations (composite method r^2^SCAN-3c).^27^ For the reaction of 1 with CS_2_ to give the adduct 2 at room temperature, the change in free enthalpy is predicted to be very small: 4 kJ mol^−1^.

The calculation predicts that the reaction is exergonic at 233 K (Δ*G*_233 K_ = −9 kJ mol^−1^). In contrast, when considering the conversion of 1 with CO_2_, clearly positive values are calculated for both temperatures, 298 K (Δ*G*_298 K_ = 23 kJ mol^−1^) and 233 K (Δ*G*_233 K_ = 11 kJ mol^−1^). Even when the pressure is increased from 1 to 10 atm, the values for the two temperatures remain positive, although slightly lower (Δ*G*_298 K_ = 17 kJ mol^−1^; Δ*G*_233 K_ = 7 kJ mol^−1^). This theoretical prediction supports the experimental finding that 1 does not react with CO_2_ to form a corresponding adduct.

**Fig. 3:**
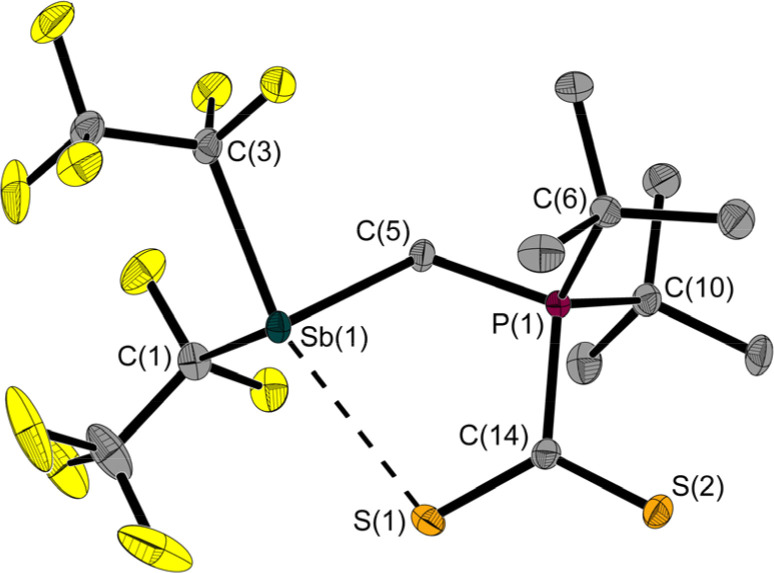
Molecular structure of 2 in the solid state. Ellipsoids are set at 50% probability; hydrogen atoms are omitted for clarity. Selected interatomic distances [Å] and angles [°]: Sb(1)–C(1) 2.265(2), Sb(1)–C(3) 2.293(2), Sb(1)–C(5) 2.193(2), P(1)–C(5) 1.791(2), P(1)–C(6) 1.870(2), P(1)–C(10) 1.875(2), P(1)–C(14) 1.844(2), S(1)–C(14) 1.681(2), S(2)–C(14) 1.657(2), Sb(1)–S(1) 2.964(1); C(1)–Sb(1)–C(3) 91.1(1), C(5)–Sb(1)–C(1) 90.4(1), C(5)–Sb(1)–C(3) 85.9(1), C(5)–P(1)–C(14) 108.4(1), P(1)–C(5)–Sb(1) 115.2(1), S(1)–C(14)–P(1) 116.6(1), S(2)–C(14)–S(1) 127.1(1), S(2)–C(14)–P(1) 116.3(1), S(1)–Sb(1)–C(3) 160.8(1).

The room temperature labile deep red crystals of 2 have been examined by X-ray diffraction. Unlike CS_2_ adducts of comparable FLPs,^5,7^ the structure of 2 is not that of a typical five-membered heterocycle (Fig. 3).

The Sb⋯S distance of 2.964(1) Å is intermediate between the sum of the *van der Waals* radii (Σ*r*_vdW_(Sb,S) = 3.86 Å)^28,29^ and the sum of the covalent radii (Σ*r*_covalent_(Sb,S) = 2.45 Å) with a tendency towards the latter. The attractive Sb⋯S interaction leads to a quasi-five-membered ring. The S(1)–Sb(1)–C(3) angle of 160.8(1)° identifies the Sb⋯S interaction as a weak pnictogen bond with a deviation from 180° expected for a σ-hole-type interaction. The P–C–Sb angle in 2 at 115.2(1)° is larger than in 1 at 110.6(1)°.

Another even weaker (intermolecular) interaction is between Sb(1) and S(2)’ (symmetry: 1 − *x*, 1 − *y*, 1 − *z*; Fig. S41†). The distance of 3.640(1) Å is slightly below the sum of the *van der Waals* radii (Σ*r*_vdW_(Sb,S) = 3.86 Å).^28,29^ This interaction presumably also contributes to the non-formation of a five-membered ring.

In order to better describe the interaction of the two heteroatoms Sb(1) and S(1), quantum chemical calculations were carried out. A Quantum Theory of Atoms in Molecules (QTAIM, PBE0/def2-TZVPP)^30^ analysis gives a bond path for the Sb(1)⋯S(1) interaction with a not-so-small value for the charge density at the bond critical point (BCP) of 0.27*e* Å^−3^ compared to the value of the Sb–C bond of 0.67*e* Å^−3^ and other similar systems like Me_2_Sb–SMe (*ρ*_BCP_ (Sb–S) 0.61*e* Å^−3^) or the adduct of the anthracene based, (F_5_C_2_)_2_Sb–C≡C– substituted poly-Lewis acid with SMe_2_ (*ρ*_BCP_ (Sb⋯S) 0.11/0.16*e* Å^−3^).^17^

This confirms the classification as half covalent, which is supported by the distance criterion. The corresponding Laplacian ∇^2^*ρ*_BCP_(Sb–S) has a small value of 0.96*e* Å^−5^.

**Fig. 4:**
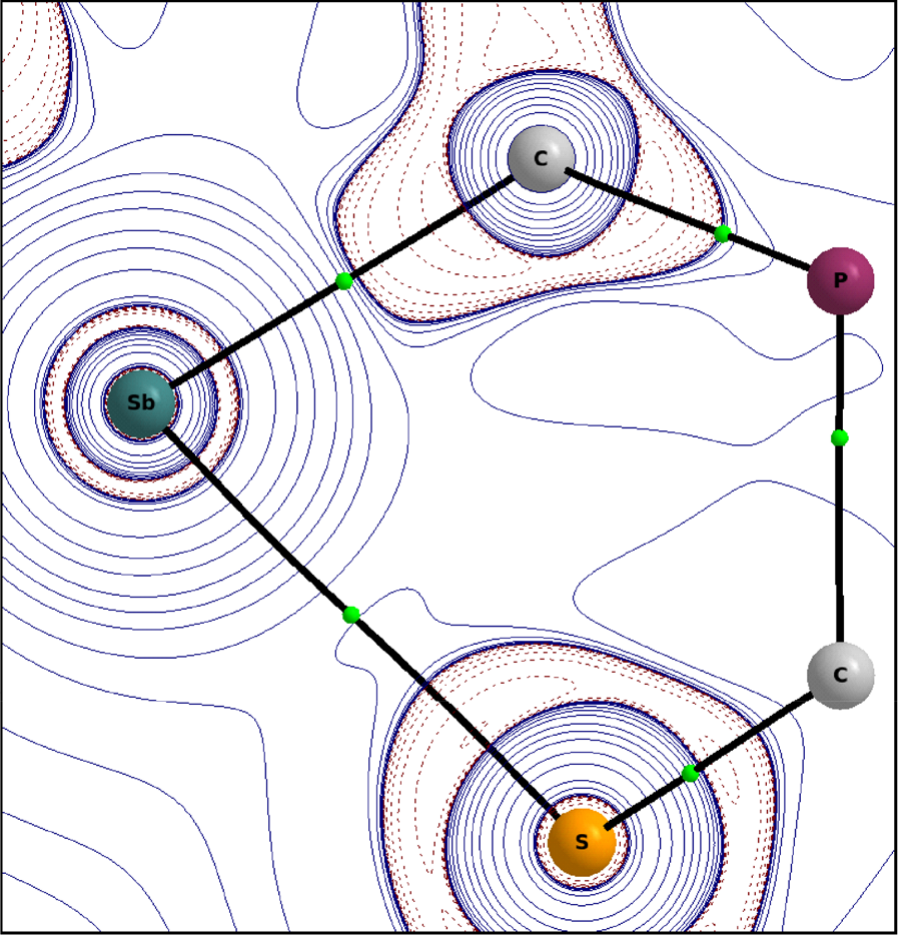
Contour plot of the Laplacian ∇^2^*ρ* (positive isovalues are printed in full blue and negative ones as dashed red lines) of the electron density in the C(5)–Sb(1)–S(1) plane of 2.

Based on the results of the QTAIM^30^ and IQA (Interacting Quantum Molecules)^31^ analyses (Tables 1, S2,†Fig. 4), the interaction can be described as weakly stabilising, polar and partially covalent. For classification purposes we calculated reference systems, which are listed in the ESI.†

**Table 1:** Results of QTAIM^30^ analyses for selected atom pairs in adducts 2 and 6. Electron density at bond critical point, *ρ*_BCP_ [*e* Å^−3^], and Laplacian of the electron density at bond critical point, ∇^2^*ρ*_BCP_ [*e* Å^−5^]. Note that the numeration in the ESI is partly different. For more details, see the ESI

Compound	Atom pair A–B	*ρ*_BCP_	∇^2^*ρ*_BCP_
2	Sb(1)–S(1)	0.27	0.96
	Sb(1)–C(5)	0.67	1.69
	S(1)–C(14)	1.55	−9.64
6	Au(1)–P(1)	0.73	1.66
	Au(1)–P(2)	0.72	1.66
	Au(1)–Cl(1)	0.22	2.19
	Sb(1)–Cl(1)	0.19	1.20
	Sb(2)–Cl(1)	0.18	1.13

The interaction energy *E*^AB^_int_ of the atom pair Sb–S with −8.20 × 10^−2^ a.u., lies between those of Li–F (*E*^AB^_int_ = −3.27 × 10^−1^ a.u.) and Xe–Xe (*E*^AB^_int_ = −5.78 × 10^−3^ a.u.) as reference values for typical ionic and typical dispersion interactions. As with the interaction in the dixenon molecule, the majority of the Sb–S interaction energy (84%) is due to electron exchange and correlation effects (see ESI† for more details).

In order to be able to make a statement about the influence of the Lewis acid on the reactivity of the phosphorus Lewis base towards CS_2_ and the formation of a corresponding adduct, additional DFT calculations^27^ were carried out. Due to the presence of the Lewis acid site in 1, the reaction 1 + CS_2_ → 1·CS_2_ (Δ*H*_298K_ = −53 kJ mol^−1^) is significantly more exothermic than a comparable reaction of a phosphane of similar constitution around phosphorus, namely di-*tert*-butylmethylphosphane, with CS_2_ (Δ*H*_298 K_ = −13 kJ mol^−1^).

Although both reactions are endergonic, the reaction with di-*tert*-butylmethylphosphane, *i.e.* without the influence of a Lewis acid, is significantly more endergonic (Δ*G*_298 K_ = 36 kJ mol^−1^). The theoretical values thus indicate that the Sb⋯S interaction, although weak, supports the adduct formation.

**Fig. 5:**
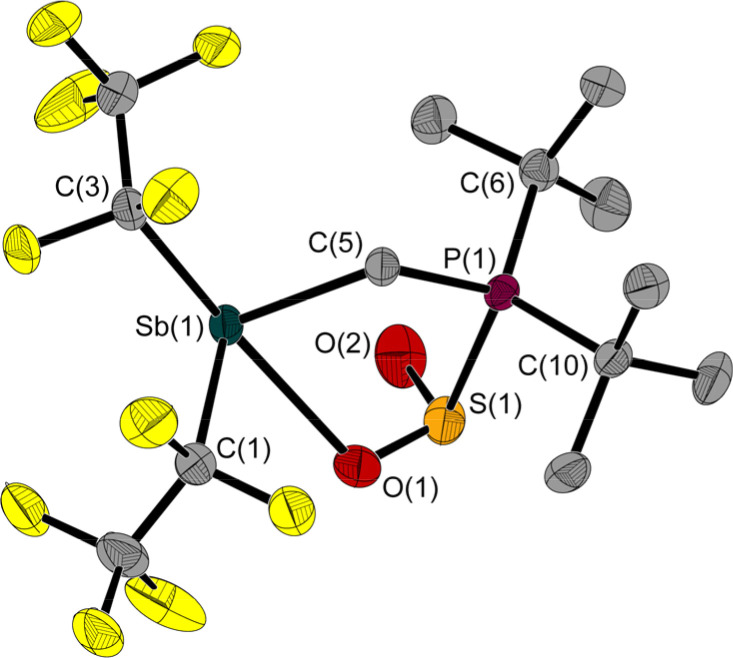
Molecular structure of 3 in the solid state. Ellipsoids are set at 50% probability; hydrogen atoms and minor occupied disordered atoms are omitted for clarity. Selected bond lengths [Å] and angles [°]: Sb(1)–O(1) 2.483(2), Sb(1)–C(1) 2.242(3), Sb(1)–C(3) 2.281(3), Sb(1)–C(5) 2.199(2), S(1)–P(1) 2.285(1), S(1)–O(1) 1.498(2), S(1)–O(2) 1.470(2), P(1)–C(5) 1.803(2), P(1)–C(6) 1.852(3), P(1)–C(10) 1.857(2); C(1)–Sb(1)–O(1) 77.1(1), C(1)–Sb(1)–C(3) 87.7(1), C(3)–Sb(1)–O(1) 158.5(1), C(5)–Sb(1)–O(1) 78.0(1), C(5)–Sb(1)–C(1) 95.5(1), C(5)–Sb(1)–C(3) 88.6(1), O(1)–S(1)–P(1) 95.1(1), O(2)–S(1)–P(1) 102.8(1), O(2)–S(1)–O(1) 111.8(1), C(5)–P(1)–S(1) 104.3(1), S(1)–O(1)–Sb(1) 112.9(1), P(1)–C(5)–Sb(1) 114.9(1).

The reaction of 1 with SO_2_ gives the adduct 3 with a five-membered heterocycle and an exocyclic S–O bond (Fig. 5). The angles around the sulphur atom add up to 309.7(3)°, describing distortion of the trigonal pyramidal coordination environment. The Sb atom is bisphenoidally surrounded with a C(3)–Sb(1)–O(1) angle of 158.5(1)° and a C(5)–Sb(1)–C(1) angle of 95.5(1)°.

Similar to 2, the Sb(1)–C(5)–P(1) angle of 114.9(1)° in 3 is wider than in 1. The ^31^P NMR chemical shift of 3 is 39.8 ppm, which is in a typical range for P atoms with a similar substitution pattern (*e.g.*: *δ*(^31^P) (F_5_C_2_)_3_SnCH_2_P(*t*Bu)_2_·SO_2_: 47.4 ppm ^5^).

**Fig. 6:**
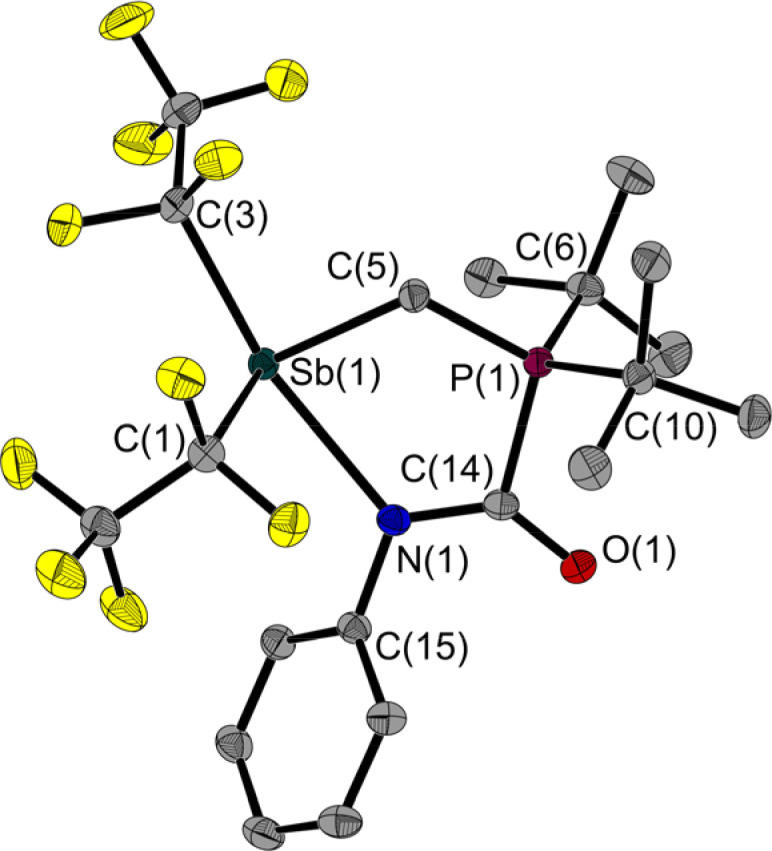
Molecular structure of 4 in the solid state. Ellipsoids are set at 50% probability; hydrogen atoms are omitted for clarity. Selected bond lengths [Å] and angles [°]: Sb(1)–N(1) 2.473(1), Sb(1)–C(1) 2.253(1), Sb(1)–C(3) 2.332(1), Sb(1)–C(5) 2.180(1), P(1)–C(5) 1.792(1), P(1)–C(6) 1.856(1), P(1)–C(10) 1.852(1), P(1)–C(14) 1.860(1), O(1)–C(14) 1.243(2), N(1)–C(14) 1.322(2), N(1)–C(15) 1.428(2); C(1)–Sb(1)–N(1) 84.0(1), C(3)–Sb(1)–N(1) 159.7(1), C(5)–Sb(1)–N(1) 78.1(1), C(5)–P(1)–C(14) 108.0(1), C(14)–N(1)–Sb(1) 121.5(1), C(14)–N(1)–C(15) 117.7(1), C(15)–N(1)–Sb(1) 120.8(1), P(1)–C(5)–Sb(1) 113.6(1), O(1)–C(14)–P(1) 116.7(1), O(1)–C(14)–N(1) 132.0(1), N(1)–C(14)–P(1) 111.3(1).

Surprisingly, the addition of phenyl isocyanate to 1 does not proceed *via* the C=O bond but *via* the C=N bond. This results in a five-membered heterocycle with exocyclic C=O and N–C_*ipso*_ bonds (Fig. 6).

However, in the light of the HSAB concept, this behaviour is to be expected: comparing the O and N atoms of the phenyl isocyanate, the latter is the softer one and should therefore be preferred to interact with the soft Sb atom. In 4, the Sb(1)–C(5)–P(1) angle is also widened at 113.6(1)° compared to the free FLP 1. In addition to the product signal set, the dissolved NMR sample contains a small proportion of the two reactants. IR spectroscopy was used to compare the isolated product as a solid and dissolved in CCl_4_ with a solution of phenyl isocyanate in CCl_4_. A band characteristic of phenyl isocyanate was detected in both solutions, but not in the solid sample. The isolated product 4 seems to decompose to a small extent into its reactants by dissolution.

**Fig. 7:**
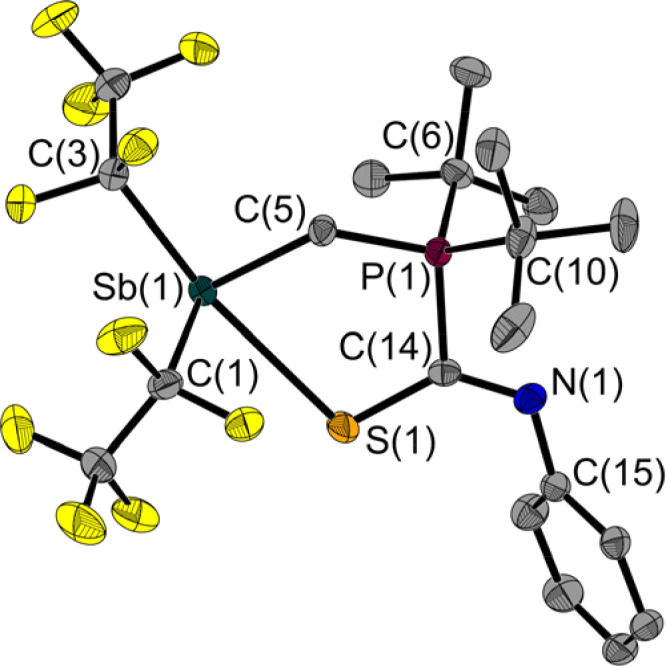
Molecular structure of 5a in the solid state. Only one of the two molecules in the asymmetric unit is shown. Ellipsoids are set at 50% probability; hydrogen atoms are omitted for clarity. Selected bond lengths [Å] and angles [°]: Sb(1)–S(1) 2.881(1), Sb(1)–C(1) 2.243(2), Sb(1)–C(3) 2.311(2), Sb(1)–C(5) 2.172(2), S(1)–C(14) 1.713(2), P(1)–C(5) 1.792(2), P(1)–C(6) 1.856(2), P(1)–C(10) 1.857(2), P(1)–C(14) 1.837(2), N(1)–C(14) 1.289(2), N(1)–C(15) 1.419(2); C(1)–Sb(1)–S(1) 86.1(1), C(3)–Sb(1)–S(1) 164.7(1), C(5)–Sb(1)–S(1) 80.1(1), C(14)–S(1)–Sb(1) 102.8(1), C(5)–P(1)–C(14) 109.4(1), C(14)–N(1)–C(15) 122.2(2), P(1)–C(5)–Sb(1) 119.3(1), S(1)–C(14)–P(1) 118.0(1), N(1)–C(14)–S(1) 131.8(1); N(1)–C(14)–P(1) 110.1(2).

In contrast to the addition of phenyl isocyanate described above, 1 reacts only to a small extent with phenyl isothiocyanate at the C=N but mainly at the C=S double bond (Fig. 7). The main product 5a has a five-membered heterocycle, but in this case with an exocyclic C=N-Ph unit. This is consistent with the predictions of the HSAB concept. Compared to the other adducts presented here, the largest Sb(1)–C(5)–P(1) angle is found in 5a with 119.3(1)°. The Sb(1)–S(1) bond with 2.881(1) Å is shorter than that in the CS_2_ adduct 2, and thus shorter than the sum of the *van der Waals* radii (Σ*r*_vdW_(Sb–S) = 3.86 Å).^28,29^ Again, the Sb atom is bisphenoidally surrounded, with the C(3)–Sb(1)–S(1) angle of 164.7(1)° in the same range as in the previously discussed adducts.

**Scheme 2:**
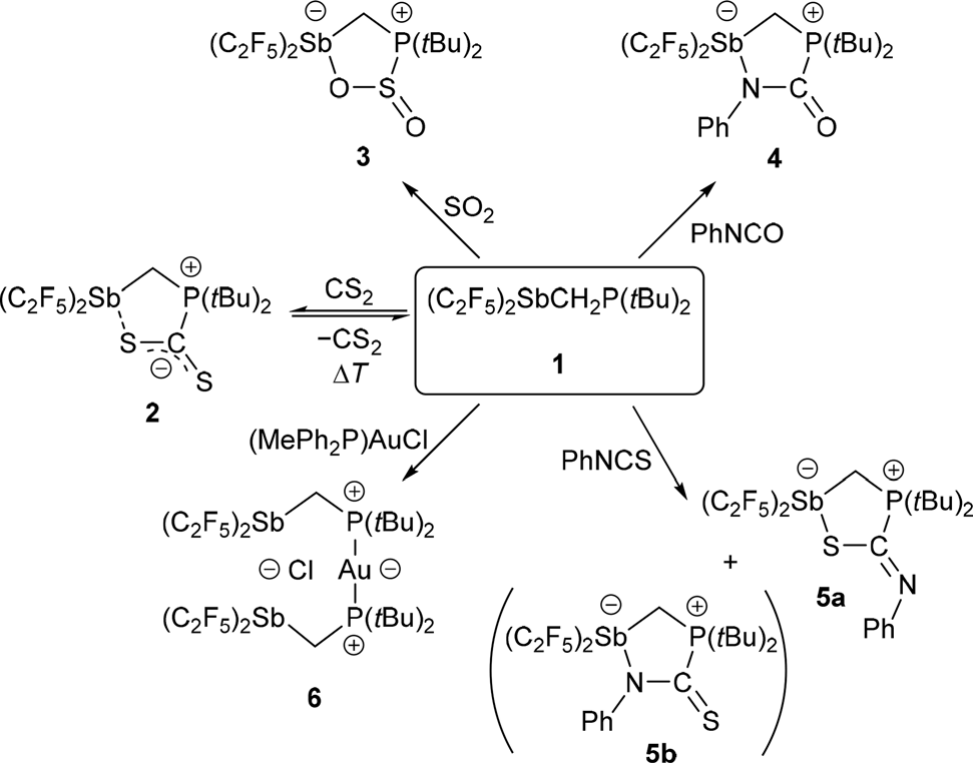
Reactions of FLP 1 with selected substrates at room temperature.

Quantum chemical calculations (DFT)^27^ on the different types of adduct formation behaviour are in agreement with the experimental results: the formation of the addition product to phenyl isocyanate 4 at the C=N bond is not significantly favoured in terms of energy (4: Δ*G* = −10 kJ mol^−1^) compared to the addition at the C=O bond (Δ*G* = −6 kJ mol^−1^). The same is true for the addition to phenyl isothiocyanate (5a: Δ*G* = −16 kJ mol^−1^; C=N addition product 5b: Δ*G* = −11 kJ mol^−1^), so it is not surprising that addition to both sites is experimentally observed and not exclusively adduct 5a is formed (Scheme 2). In the NMR spectra of the isolated solid of this reaction, there is a second set of signals with only slightly different chemical shifts, representing about a quarter of the mixture. The recorded data allow the following considerations: The connectivity of 5a and the related tin compound, the adduct (F_5_C_2_)_3_SnCH_2_P(*t*Bu)_2_·PhNCS, is analogous; consequently, there is a clear similarity in the chemical shifts of the respective isothiocyanate carbon atom and the *ipso* carbon atoms (Table 2). In contrast, 5b shows larger deviations from the data of (F_5_C_2_)_3_SnCH_2_P(*t*Bu)_2_·PhNCS.

**Table 2:** Chemical shifts *δ* [ppm] of the PhN**C**E (E = O, S) and the *ipso* carbon atoms of the adducts of 1 with PhNCS and PhNCO and of (F_5_C_2_)_3_SnCH_2_P(*t*Bu)_2_ with its respective adducts^5,20^

Compound	*δ*(^13^C) PhNCE	*δ*(^13^C) C_*ipso*_
1·PhNCS (5a)	168.7	151.0
1·PhNCS (5b)	178.2	147.3
1·PhNCO (4)	160.1	144.6
(F_5_C_2_)_3_SnCH_2_P(*t*Bu)_2_·PhNCS	164.3	150.0
(F_5_C_2_)_3_SnCH_2_P(*t*Bu)_2_·PhNCO	150.0	146.4

We also analysed the adducts of phenyl isocyanate 4 and phenyl isothiocyanate 5a/5b using two-dimensional NMR techniques (^15^N ^1^H HMBC).

The spectra contained signals of the free phenyl isocyanate or phenyl isothiocyanate, respectively, which were used as additional references for these samples. For both samples we observed only one other cross peak. These were very different from the chemical shifts of the reactants (*δ*(^15^N) of 4 : 150.3 ppm, *δ*(^15^N) of PhNCO: 48.4 ppm; *δ*(^15^N) of 5a: 345.2 ppm, *δ*(^15^N) of PhNCS: 107.7 ppm) and also from each other; for 5b we would expect a less significant deviation compared to 4. Probably due to too low a concentration and a possible different relaxation behaviour, we could not detect a cross peak attributable to 5b.

An attempt to assign the IR spectroscopic data of the adducts by using the results of quantum chemical calculations failed due to the lack of characteristic vibrational bands; a more precise identification of the constitutional isomers 5a and 5b is therefore not possible.

We note that a reference system for FLP 1 without Lewis acid function, namely MeP*t*Bu_2_, shows no reactions with the substrates SO_2_ and PhNCO, whereas it forms equilibria of adducts and precursors with CS_2_ and PhNCS. The fact that all adducts of 1 are more stable than those of MeP*t*Bu_2_ demonstrates that the antimony function in 1 (*i.e.* its FLP nature) is crucial for adduct formation with SO_2_ and PhNCO and highly supportive for CS_2_ and PhNCS.

**Fig. 8:**
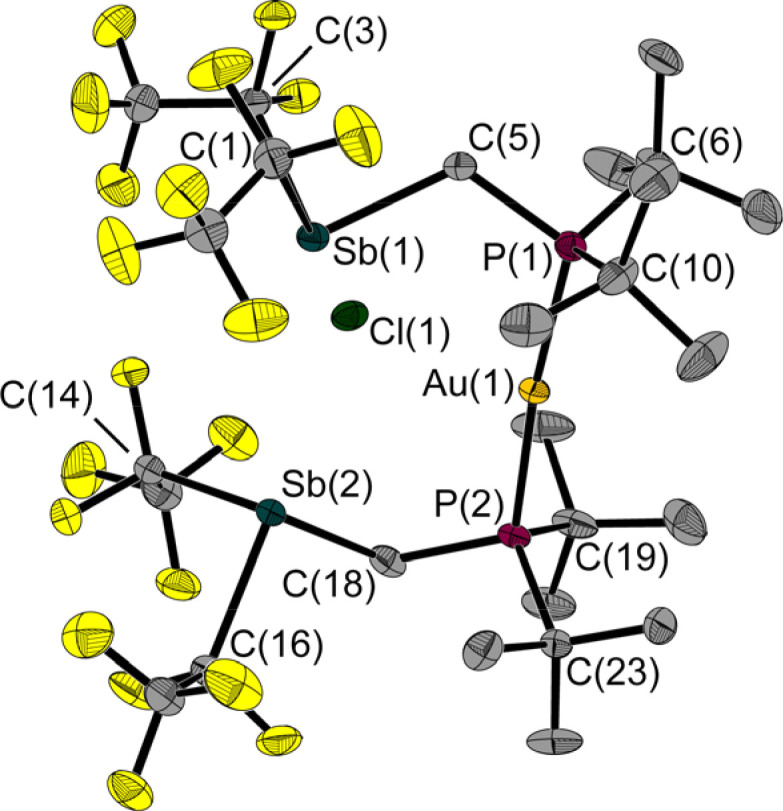
Molecular structure of 6 in the solid state. Ellipsoids are set at 50% probability; hydrogen atoms and minor occupied disordered atoms are omitted for clarity. Selected bond lengths and interatomic distances [Å] and angles [°]: Au(1)–P(1) 2.311(1), Au(1)–P(2) 2.319(1), Sb(1)–C(1) 2.309(3), Sb(1)–C(3) 2.259(3), Sb(1)–C(5) 2.169(2), Sb(2)–C(14) 2.173(5), Sb(2)–C(16) 2.310(3), Sb(2)–C(18) 2.153(3), P(1)–C(5) 1.823(3), P(2)–C(18) 1.826(3), Au(1)⋯Cl(1) 2.939(1), Cl(1)⋯Sb(1) 2.966(1), Cl(1)⋯Sb(2) 2.981(1); P(1)–Au(1)–P(2) 174.6(1), C(3)–Sb(1)–C(1) 89.7(1), C(5)–Sb(1)–C(1) 93.3(1), C(5)–Sb(1)–C(3) 86.9(1), C(14)–Sb(2)–C(16) 89.8(1), C(18)–Sb(2)–C(16) 91.0(1), C(18)–Sb(2)–C(14) 107.1(2), C(5)–P(1)–Au(1) 112.7(1), C(18)–P(2)–Au(1) 113.7(1), P(1)–C(5)–Sb(1) 118.6(1), P(2)–C(18)–Sb(2) 117.8(1), Sb(1)⋯P(1)⋯P(2)⋯Sb(2) 4.8(1).

In a reaction of 1 with the phosphane-gold chloride (MePh_2_P)AuCl, two molecules of the free FLP reacted with one molecule of the gold compound, the MePPh_2_ being displaced by the phosphane function of the FLP (Fig. 8). An almost linear coordinated gold atom is obtained; the P(1)–Au(1)–P(2) angle is 174.6(1)°, similar to other gold(i) complexes with two phosphane ligands.^32,33^ The distance between the gold and chlorine atom (2.939(1) Å) is greater than the sum of the corresponding covalence radii of 2.23 Å,^23^ but well below the sum of the *van der Waals* radii of 3.41 Å,^29^ indicating an attractive interaction between these two atoms. A distance shorter than the sum of the *van der Waals* radii is also found between the chlorine and the two antimony atoms (Cl(1)–Sb(1): 2.966(1) Å, Cl(1)–Sb(2): 2.981(1) Å, Σ*r*_vdW_(Sb–Cl) = 3.81 Å).^28,29^ The Sb–C–P angles are 118.6(1)° and 117.8(1)°, which are wider than in the reactant 1. For molecule 6, we also performed QTAIM and IQA analyses (PBE0/def2-TZVPP) to describe the interaction of selected atom pairs (Tables 1 and S3†).^30,31^ Based on the calculations, the Au(1)–P(1/2) bonds are typically polarised bonds with a strong covalent character. In contrast the Au(1)–Cl(1) interaction can be considered as a weakly polarised bond with covalent character and a stabilisation energy about three times lower than that for Au(1)–P(1/2).

The interactions between the two antimony atoms and the chlorine atom are strongly stabilising, mainly ionic and have a stabilisation energy similar to that of the Au–P bonds. The calculations show that the P⋯Cl interactions are purely ionic and are even twice as strong as the Au(1)–Cl(1) interaction, despite the absence of bond critical points and bond paths. Also noteworthy is the presence of other low electron density bond paths for Cl⋯F and Cl⋯H (see ESI† for more details). The pronounced clamp-like structure is thus maintained not only in the solid state, but also likely to exist in the free molecules as predicted by quantum chemical optimisations. This seems to be due to the stabilising interactions between the FLP clamp and the chlorine atom.

To see if this structural motif was also present without the chloride anion, we reacted compound 6 with silver triflate (AgOTf). By replacing the chloride anion with triflate, compound 7 was obtained as a colourless solid.

**Fig. 9:**
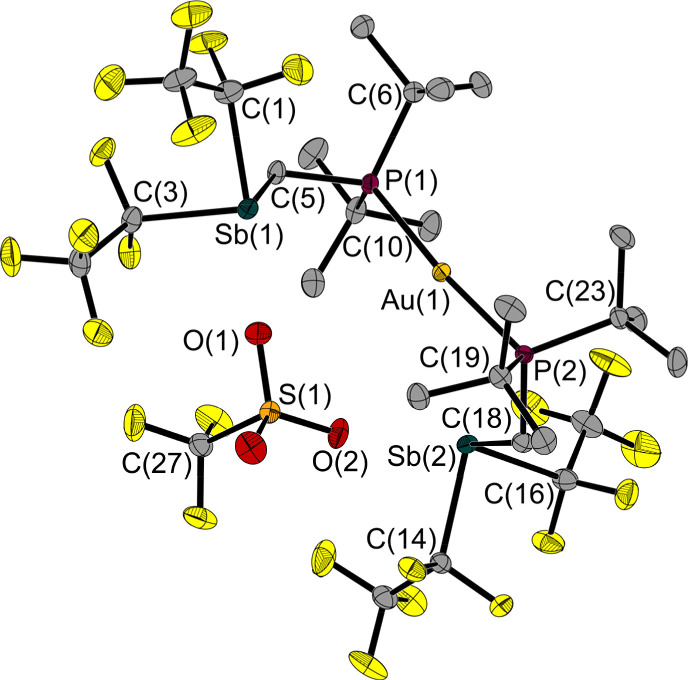
Molecular structure of 7 in the solid state. Ellipsoids are set at 50% probability; hydrogen atoms are omitted for clarity. Selected bond lengths and interatomic distances [Å] and angles [°]: Au(1)–P(1) 2.328(1), Au(1)–P(2) 2.328(1), Sb(1)–C(1) 2.275(2), Sb(1)–C(3) 2.254(2), Sb(1)–C(5) 2.164(2), Sb(2)–C(14) 2.254(2), Sb(2)–C(16) 2.268(2), Sb(2)–C(18) 2.172(2), P(1)–C(5) 1.826(2), P(2)–C(18) 1.831(2), Au(1)⋯O(2) 3.521(2), O(1)⋯Sb(1) 2.647(2), O(2)⋯Sb(2) 2.808(2); P(1)–Au(1)–P(2) 173.9(1), C(3)–Sb(1)–C(1) 92.3(1), C(5)–Sb(1)–C(1) 91.8(1), C(5)–Sb(1)–C(3) 87.1(1), C(14)–Sb(2)–C(16) 89.8(1), C(18)–Sb(2)–C(16) 96.7(1), C(18)–Sb(2)–C(14) 87.6(1), C(5)–P(1)–Au(1) 116.8(1), C(18)–P(2)–Au(1) 111.7(1), P(1)–C(5)–Sb(1) 120.4(2), P(2)–C(18)–Sb(2) 115.8(2), Sb(1)⋯P(1)⋯P(2)⋯Sb(2) 111.1(1).

Its structure in the solid state shows no clamp-like shape (Fig. 9); the torsion angle (Sb–P–P–Sb) over the P axis is 106° wider than in 6. The P(1)–Au(1)–P(2) angle of 6 (174.6(1)°) is almost identical to that of 7 (173.9(1)°).

The distance between the gold atom and the nearest oxygen atom of the triflate is 3.521(2) Å and thus exceeds the sum of the *van der Waals* radii (Σ*r*_vdW_(Au–O) = 3.18 Å).^29^ In turn, the distances between the antimony atoms and the nearest oxygen atoms of the anion fall below (O(1)–Sb(1): 2.647(2) Å, O(2)–Sb(2): 2.808(2) Å, Σ*r*_vdW_(Sb–O) = 3.58 Å).^27,28^ The ^31^P NMR chemical shifts of the two gold adducts of this work (*δ*(^31^P) 6: 74.0 ppm, 7: 78.3 ppm) and the Sn FLP adduct (*δ*(^31^P) (F_5_C_2_)_3_SnCH_2_P(*t*Bu)_2_·AuCl(PPh_3_) 74.4 ppm)^5^ are in a similar range.

## Conclusions

We present here the neutral pre-organised Sb/P-Lewis pair (F_5_C_2_)_2_SbCH_2_P(*t*Bu)_2_ (1) capable of forming the corresponding 1,2-addition products with various substrates, including CS_2_, SO_2_ and PhNCS, and the 2,3-addition product with PhNCO. The relatively soft acidic Sb(iii)-Lewis function allows reversible binding of CS_2_, whereas an adduct formation with CO_2_ was not observed under similar conditions; an evaluation of the energy contributions to both reactions by quantum chemical calculations explains this experimental finding: the difference in free enthalpy for the formation of the CS_2_ adduct at 298 K is 19 kJ mol^−1^ less than for the formation of the analogous CO_2_ adduct and therefore its formation is much more favourable. These results are consistent with qualitative predictions from the HSAB concept. QTAIM and IQA analyses found a bond path with a bond critical point for the Sb–S interaction in the distorted five-membered ring adduct (F_5_C_2_)_2_SbCH_2_P(*t*Bu)_2_·CS_2_ and predicted its stabilisation energy to be −8.20 × 10^−2^ a.u.

For the adduct formation of the FLP with phenyl isocyanate and phenyl isothiocyanate, we found a preference for the adduct favoured by the HSAB concept, although the energetic difference between the different addition products is not significant. In the case of phenyl isothiocyanate both possible adducts are formed.

During adduct formation with (MePh_2_P)AuCl, the phosphorus base of the gold moiety is displaced by a second FLP molecule, resulting in a stabilising clamp-like structure. Replacing the chloride anion with the larger triflate ion twists the FLP arms by 106° – another proof of the soft acid properties of 1.

## Data availability

The data published in this contribution are available as ESI, submitted with the manuscript. Crystallographic data have been deposited with the Cambridge Crystal Structure Database (CCDC).†

## Author contributions

J. Krieft: investigation, methodology, validation, visualization, writing (original draft), P. C. Trapp: investigation (DFT), Y. V. Vishnevskiy: investigation (QTAIM, IQA), B. Neumann: investigation (SCXRD), H.-G. Stammler: investigation (SCXRD), J.-H. Lamm: investigation (SCXRD), N. W. Mitzel: funding, acquisition, project administration, supervision, reviewing and editing.

## Conflicts of interest

There are no conflicts to declare.

## Supplementary Material

SC-015-D4SC02785J-s001

SC-015-D4SC02785J-s002
