# Supplementary material for: A geminal antimony(iii)/phosphorus(iii) frustrated Lewis pair
Source: Chem Sci. 2024 Jul 1;15(30):12118–25. doi: 10.1039/d4sc02785j (PMC11290451; doi:10.1039/d4sc02785j)
Supplement: SC-015-D4SC02785J-s001 [file SC-015-D4SC02785J-s001.pdf]

Electronic Supplementary Material (ESI) for Chemical Science.
This journal is © The Royal Society of Chemistry 2024

Supporting Information

A Geminal Antimony(III)/Phosphorus(III) Frustrated Lewis Pair

Jonas Krieft, Pia C. Trapp, Yury V. Vishnevskiy, Beate Neumann, Hans-Georg Stammler, Jan-Hendrik Lamm and Norbert W. Mitzel*

*Corresponding Author.

Chair of Inorganic and Structural Chemistry, Center for Molecular Materials CM_2

Faculty of Chemistry, Bielefeld University

Universitätsstraße 25, 33615 Bielefeld (Germany)

E-mail: mitzel@uni-bielefeld.de

Table of Contents

Experimental Procedures

NMR Spectroscopy Data

IR Spectroscopy Data

Crystallographic Data

Quantum Chemical Calculations

References

Experimental Procedures

General Information

All operations with air- and moisture-sensitive substances were performed under conventional Schlenk techniques or in a glove box using argon as inert gas. Volatile compounds were handled in a vacuum line. All solvents (diethyl ether and *n*-pentane dried over $LiAlH_4$, *n*-hexane, toluene, benzene-d_6 and toluene-d_8 dried over Na/K alloy, dichloromethane-d_2, toluene-d_8, benzene-d_6 and tetrachloromethane dried over molecular sieves) were distilled and degassed prior to use. $(F_5C_2)_2SbCl$,[1] $LiCH_2P(tBu)_2$[2] and $(MePPh_2)AuCl$[3] were prepared according to literature procedures. CS_2 (99.9 %, J. T. Baker) was dried over P_4O_{10}, distilled and degassed prior to use. SO_2 (99.98 %, Air Liquide), PhNCO (≥99.0 %, Sigma Aldrich), PhNCS (>99 %, fluorochem) and silver trifluoromethanesulfonate (99 %, fluorochem) were used without further purification. NMR spectra were recorded using Bruker Avance III 500 HD and Bruker Avance III 600 spectrometers at ambient temperature if not noted otherwise. NMR spectroscopic chemical shifts were referenced to the residual proton or carbon peaks of the solvent (CD_2Cl_2: ^1H: 5.32 ppm, ^{13}C: 54.0 ppm; $C_6D_5(CD_3)$: ^1H: 2.09 ppm (CD_3), ^{13}C: 20.4 ppm (CD_3), C_6D_6: ^1H: 7.16 ppm, ^{13}C: 128.1 ppm) or externally (^{15}N: NH_3 (l), ^{19}F: $CFCl_3$, ^{31}P: 85% H_3PO_4 in H_2O). Elemental analyses were carried out by a co-worker of the University of Bielefeld using a HEKATECH EURO Element Analyser. The elemental analyses were determined from samples of the isolated compounds. IR spectroscopic measurements were performed on a Bruker-Alpha-FT-IR spectrometer with a diamond crystal. SC-XRD was performed on a Rigaku Supernova diffractometer using Cu-Kα or Mo-Kα radiation.

Syntheses

Synthesis of $(F_5C_2)_2SbCH_2P(tBu)_2$ (1):

(Lithiomethyl)bis(*tert*-butyl)phosphane (1.58 g, 9.45 mmol) was suspended in diethyl ether (100 mL) and cooled to −78 °C. A solution of bis(pentafluoroethyl)chlorostibane (4.35 g, 8.93 mmol) and toluene in diethyl ether (50 mL) was added dropwise. The mixture was allowed to reach room temperature overnight to give a pale brown suspension. After removing the solvent under reduced pressure, *n*-pentane (50 mL) was added and the suspension was filtered inert. The solvent was removed from the filtrate under reduced pressure. Vacuum distillation (0.05 mbar, 55 °C) yielded $(F_5C_2)_2SbCH_2P(tBu)_2$ (**1**, 2.46 g, 4.74 mmol, 53 %) as a colourless liquid.

Analytical data:

^{1}H NMR (600 MHz, CD_2Cl_2): δ [ppm] = 2.17 (d, $^2J_{P,H}$ = 1 Hz, 2H, C**H**$_2$), 1.17 (d, $^3J_{P,H}$ = 11 Hz, 18H, C(C**H**$_3$)$_3$).

^{13}C{^{1}H} NMR (151 MHz, CD_2Cl_2): δ [ppm] = 122.0 (m, $^1J_{F,C}$ = 315 Hz, $^2J_{F,C}$ = 45 Hz, $^3J_{P,C}$ = 12 Hz, **C**F$_2$), 120.3 (qt, $^1J_{F,C}$ = 284 Hz, $^2J_{F,C}$ = 29 Hz, **C**F$_3$), 33.2 (d, $^1J_{P,C}$ = 24 Hz, **C**(CH$_3$)$_3$), 29.7 (d, $^2J_{P,C}$ = 13 Hz, C(**C**H$_3$)$_3$), 9.0 (d, $^1J_{P,C}$ = 44 Hz, **C**H$_2$).

^{19}F NMR (565 MHz, CD_2Cl_2): δ [ppm] = −82.7 (m, C**F**$_3$), −110.5/−111.1 (m, AB-spin system, C**F**$_2$, broad).

^{31}P{^{1}H} NMR (243 MHz, CD_2Cl_2): δ [ppm] = 15.9 (m).

Elemental analysis calcd (%) for $C_{13}H_{20}F_{10}PSb$ (M_r = 519.02): C 30.08, H 3.88; found: C 30.12, H 3.87.

$(F_5C_2)_2SbCH_2P(tBu)_2·CS_2$ (2):

A solution of **1** (0.25 g, 0.48 mmol) in *n*-pentane (5 mL) treated with CS_2 (0.3 mL, 5 mmol) was stirred at ambient temperature for 1 h. Crystallisation from the quiescent solution yielded $(F_5C_2)_2SbCH_2P(tBu)_2·CS_2$ (**2**, 0.16 g, 0.31 mmol, 64 %). The deep red crystals were suitable for X-ray diffraction, but decompose slowly under reduced pressure.

For NMR data, a separate batch was prepared in a NMR tube with a solution of **1** (50 mg, 0.10 mmol) in toluene-d_8, which was treated with CS_2 (0.05 mL, 0.8 mmol); due to the equilibrium, a small amount of **1** is still present.

Analytical data:

^{1}H NMR (600 MHz, CD_2Cl_2): δ [ppm] = 2.42 (d, $^2J_{P,H}$ = 9 Hz, 2H, C**H**$_2$), 1.57 (d, $^3J_{P,H}$ = 15 Hz, 18H, C(C**H**$_3$)$_3$).

^{13}C{^{1}H} NMR (151 MHz, CD_2Cl_2): δ [ppm] = 227.8 (d, $^1J_{P,C}$ = 32 Hz, **C**S$_2$), 125.5 (m, $^1J_{F,C}$ = 317 Hz, $^2J_{F,C}$ = 44 Hz, **C**F$_2$), 121.1 (qt, $^1J_{F,C}$ = 285 Hz, $^2J_{F,C}$ = 29 Hz, **C**F$_3$), 38.5 (d, $^1J_{P,C}$ = 31 Hz, **C**(CH$_3$)$_3$), 28.8 (s, C(**C**H$_3$)$_3$), 3.5 (d, $^1J_{P,C}$ = 43 Hz, **C**H$_2$).

^{19}F NMR (565 MHz, CD_2Cl_2): δ [ppm] = −81.8 (m, C**F**$_3$), −111.8/−113.6 (m, AB-spin system C**F**$_2$, broad).

^{31}P{^{1}H} NMR (243 MHz, CD_2Cl_2): δ [ppm] = 35.2 (s).

Elemental analysis calcd (%) for $C_{14}H_{20}F_{10}PS_2Sb$ (M_r = 595.15): C 28.25, H 3.39; found: C 28.85, H 3.37.

$(F_5C_2)_2SbCH_2P(tBu)_2·SO_2$ (3):

SO_2 (1.1 mmol) was condensed onto **1** (110 mg, 212 µmol). On thawing, a colourless solid was immediately formed. After 15 min the excess of SO_2 was replaced with argon to give $(F_5C_2)_2SbCH_2P(tBu)_2·SO_2$ (**3**, 124 mg, 212 µmol, quant.) as an amorphous colourless solid, which decomposes rapidly in an argon stream or under reduced pressure. Crystals, suitable for X-ray diffraction experiments, were obtained by cooling a solution of **3** in *n*-hexane to −20 °C.

For NMR data, a separate batch was prepared in a NMR tube with a solution of **1** (56 mg, 0.11 mmol) in CD_2Cl_2, which was treated with SO_2 (0.40 mmol).

Due to decomposition, it was not possible to prepare a sample for elemental analysis.

Analytical data:

^{1}H NMR (600 MHz, CD_2Cl_2): δ [ppm] = 1.98 (d, $^2J_{P,H}$ = 13 Hz, 2H, C**H**$_2$), 1.46 (d, $^3J_{P,H}$ = 14 Hz, 18H, C(C**H**$_3$)$_3$).

^{13}C{^{1}H} NMR (151 MHz, CD_2Cl_2): δ [ppm] = 126.6–121.5 (m, $^1J_{F,C}$ = 317 Hz, $^2J_{F,C}$ = 44 Hz, **C**F$_2$), 123.9–117.8 (qt, $^1J_{F,C}$ = 285 Hz, $^2J_{F,C}$ = 29 Hz, **C**F$_3$), 37.9 (d, $^1J_{P,C}$ = 3 Hz, **C**(CH$_3$)$_3$), 28.5 (s, C(**C**H$_3$)$_3$), −2.2 (d, $^1J_{P,C}$ = 10 Hz, **C**H$_2$).

^{13}C{^{19}F} NMR (126 MHz, CD_2Cl_2): δ [ppm] = 124.0 (s, **C**F$_2$), 120.9 (s, **C**F$_3$).

^{19}F NMR (565 MHz, CD_2Cl_2): δ [ppm] = −82.1 (m, C**F**$_3$), −114.0/−114.5 (m, AB-spin system C**F**$_2$, broad).

^{31}P{^{1}H} NMR (243 MHz, CD_2Cl_2): δ [ppm] = 43.7 (s).

$(F_5C_2)_2SbCH_2P(tBu)_2$·PhNCO (4):

A solution of **1** (155 mg, 300 µmol) in dichloromethane (2 mL) was treated with a solution of phenyl isocyanate (40 mg, 0.34 mmol) in dichloromethane (1 mL). After stirring for 1 h at ambient temperature, the solution was concentrated under reduced pressure. By addition of *n*-hexane (2 mL), a colourless solid precipitated, which was washed with *n*-hexane (3 × 1 mL) and dried under vacuum. $(F_5C_2)_2SbCH_2P(tBu)_2$·PhNCO (**4**, 178 mg) was obtained as colourless solid. NMR spectra and elemental analysis indicate a small proportion of remaining phenyl isocyanate.

Single crystals suitable for X-ray diffraction were received by cooling a saturated solution of **4** in CD_2Cl_2 down to −20 °C.

In addition to the product signal set, the dissolved NMR sample contains a small amount of the two reactants. IR analysis was used to compare the isolated product as a solid and as a solution in CCl_4 with a solution of phenyl isocyanate in CCl_4. A band characteristic of phenyl isocyanate was detected for both solutions; this was not detected in the solid sample (see below).

Due to the low natural abundance of the ^{15}N nucleus, ^{15}N ^{1}H HMBC spectra of highly concentrated samples were recorded to detect the chemical shift. A small excess of phenyl isocyanate is present in the separately recorded spectra to facilitate comparison between product and reactant.

Analytical data:

^{1}H NMR (600 MHz, CD_2Cl_2): δ [ppm] = 7.31 (m, 2H, Ph), 7.12 (m, 3H, Ph), 2.15 (d, $^{2}J_{P,H}$ = 10 Hz, 2H, C**H**$_2$), 1.58 (d, $^{3}J_{P,H}$ = 15 Hz, 18H, C(C**H**$_3$)$_3$).

^{13}C{^{1}H} NMR (151 MHz, CD_2Cl_2): δ [ppm] = 160.1 (d, $^{1}J_{P,C}$ =97 Hz, P**C**(=O)N), 144.6 (m, **C**$_{ipso}$), 129.0 (s, Ph), 126.3 (m, **C**F$_2$, broad and overlapping with another signal), 125.5 (s, Ph), 124.8 (s, Ph), 121.3 (qt, $^{1}J_{F,C}$ = 285 Hz, $^{2}J_{F,C}$ = 29 Hz, **C**F$_3$), 35.8 (d, $^{1}J_{P,C}$ = 32 Hz, **C**(CH$_3$)$_3$), 27.9 (s, C(**C**H$_3$)$_3$), −1.6 (d, $^{1}J_{P,C}$ = 32 Hz, **C**H$_2$).

^{15}N NMR (61 MHz, CD_2Cl_2): δ [ppm] = 150.3 (from ^{15}N ^{1}H HMBC).

^{19}F NMR (565 MHz, CD_2Cl_2): δ [ppm] = −82.3 (s, C**F**$_3$), −112.9/−115.4 (d, $^{2}J_{F,F}$ = 303 Hz/300 Hz, AB-spin system C**F**$_2$, broad).

^{31}P{^{1}H} NMR (243 MHz, CD_2Cl_2): δ [ppm] = 27.4 (s).

Elemental analysis calcd (%) for $C_{20}H_{25}F_{10}NOPSb$ (M_r = 638.14): C 37.64, H 3.95, N 2.19; found: C 39.39, H 4.02, N 2.62.

$(F_5C_2)_2SbCH_2P(tBu)_2$·PhNCS (5):

A solution of **1** (158 mg, 304 µmol) in dichloromethane (2 mL) was treated with a solution of phenyl isothiocyanate (48 mg, 0.36 mmol) in dichloromethane (1 mL). After stirring at ambient temperature for 1 h, the yellow solution was concentrated under reduced pressure. By adding *n*-hexane (2 mL), a yellowish solid precipitated, which was washed with *n*-hexane (3 × 1 mL) and dried in vacuum. $(F_5C_2)_2SbCH_2P(tBu)_2$·PhNCS (**5**, 191 mg, 292 µmol, 96 %) was received as yellowish solid. Single crystals suitable for X-ray diffraction were received from a saturated solution of **5** in *n*-hexane.

The NMR spectra show two sets of signals (**A** and **B** in the following) that are similar in structure, but have a slight deviation in the chemical shift and the intensity of the respective resonances. When compared with the adducts listed above, both sets of signals give reasonable variants for phenyl isothiocyanate adducts.

Attempts to separate the two species either failed or resulted in unspecific degradation.

The experiment was repeated several times with an analogous procedure, including execution at −78 °C and 80 °C, with the two species detected in a similar ratio by NMR analysis. Additional VT-NMR studies (−40–80 °C) in toluene-d_8 do not indicate a temperature dependent equilibrium of **A** and **B**. At 80 °C both **A** and **B** decomposed to their reactants.

Due to the low natural abundance of the ^{15}N nucleus, ^{15}N ^{1}H HMBC spectra of highly concentrated samples were recorded to determine the chemical shift. A small excess of phenyl isothiocyanate is present in the separately recorded spectra to facilitate comparison between product and reactant.

Analytical data:

^{1}H NMR (600 MHz, CD_2Cl_2): δ [ppm] = 7.41 – 6.91 (Ph, broad and overlap of different signals), 2.28 (d, $^{2}J_{P,H}$ = 9 Hz, 2H, **A** C**H**$_2$), 2.16 (d, $^{2}J_{P,H}$ = 9 Hz, **B** C**H**$_2$), 1.70 (d, $^{3}J_{P,H}$ = 15 Hz, **B** C(C**H**$_3$)$_3$), 1.59 (d, $^{3}J_{P,H}$ = 15 Hz, 18H, **A** C(C**H**$_3$)$_3$).

^{13}C{^{1}H} NMR (151 MHz, CD_2Cl_2): δ [ppm] = 178.2 (d, $^{1}J_{P,C}$ = 77 Hz, **B** S**C**N), 168.7 (d, $^{1}J_{P,C}$ = 91 Hz, **A** S**C**N), 151.0 (d, $^{3}J_{P,C}$ = 28 Hz, **A** **C**$_{ipso}$), 147.3 (d, $^{3}J_{P,C}$ = 22 Hz, **B** **C**$_{ipso}$), 130.2 (s), 129.3 (s), 128.6 (s), 126.3 (s), 125.4 (s), 124.0 (s), 121.8 (s), 121.4 (qt, $^{1}J_{F,C}$ = 285 Hz, $^{2}J_{F,C}$ = 29 Hz, **A** **C**F$_3$), 37.7 (d, $^{1}J_{P,C}$ = 31 Hz, **A** and **B** **C**(CH$_3$)$_3$, ^{13}C ^{1}H HMBC shows overlap of signals), 28.5 (s, **A** and **B** C(**C**H$_3$)$_3$, ^{13}C ^{1}H HMQC shows overlap of signals), −0.3 (d, $^{1}J_{P,C}$ = 51 Hz, **A** **C**H$_2$), −2.3 (d, $^{1}J_{P,C}$ = 34 Hz, **B** **C**H$_2$). (**A** and **B** **C**F$_2$ and **B** **C**F$_3$ are not observable, due to low intensity and overlapping with other signals).

^{15}N NMR (61 MHz, CD_2Cl_2): δ [ppm] = 345.2 (**A**, from ^{15}N ^{1}H HMBC).

^{19}F NMR (565 MHz, CD_2Cl_2): δ [ppm] = −81.6 (m, **A** C**F**$_3$), −82.4 (m, **B** C**F**$_3$), −112.5 – −115.6 (m, AB-spin systems **A** and **B** C**F**$_2$, broad and overlapping).

^{31}P{^{1}H} NMR (243 MHz, CD_2Cl_2): δ [ppm] = 33.3 (s, **A**), 30.1 (s, **B**).

Elemental analysis calcd (%) for $C_{20}H_{25}F_{10}NPSSb$ (M_r = 654.20): C 36.72, H 3.85, N 2.14, S 4.90; found: C 36.80, H 3.84, N 2.07, S 4.91.

[(F$_5$C$_2$)$_2$SbCH$_2$P(*t*Bu)$_2$]$_2$·AuCl (6):

A solution of **1** (137 mg, 264 µmol) in toluene (5 mL) was treated with (MePPh$_2$)AuCl (55 mg, 0.13 mmol). After stirring at ambient temperature for 1 h, all volatile compounds were removed in vacuum and the obtained residue was washed with toluene (2 × 2 mL) to yield [(F$_5$C$_2$)$_2$SbCH$_2$P(*t*Bu)$_2$]$_2$·AuCl (**6**, 165 mg, 130 µmol, quant.) as colourless solid. Single crystals suitable for X-ray diffraction were received from a saturated solution of **6** in CD_2Cl_2.

Analytical data:

^{1}H NMR (600 MHz, CD_2Cl_2): δ [ppm] = 2.52 (m, 4H, C**H**$_2$), 1.41 (m, 36H, C(C**H**$_3$)$_3$).

^{13}C{^{1}H} NMR (151 MHz, CD_2Cl_2): δ [ppm] = 127.2–122.1 (m, $^{1}J_{F,C}$ = 317 Hz, $^{2}J_{F,C}$ = 44 Hz, **C**F$_2$), 122.0–117.8 (qt, $^{1}J_{F,C}$ = 285 Hz, $^{2}J_{F,C}$ = 29 Hz, **C**F$_3$), 37.3 (m, **C**(CH$_3$)$_3$), 29.9 (m, C(**C**H$_3$)$_3$), 7.5 (m, **C**H$_2$).

^{13}C{^{19}F} NMR (126 MHz, CD_2Cl_2): δ [ppm] = 124.7 (m, **C**F$_2$), 120.9 (s, **C**F$_3$).

^{19}F NMR (565 MHz, CD_2Cl_2): δ [ppm] = −81.7 (m, C**F**$_3$), −109.5/−111.5 (m, AB-spin system C**F**$_2$, broad).

^{31}P{^{1}H} NMR (243 MHz, CD_2Cl_2): δ [ppm] = 74.0 (s).

Elemental analysis calcd (%) for $C_{26}H_{40}AuClF_{20}P_2Sb_2$ (M_r = 1270.46): C 24.58, H 3.17; found: C 24.61, H 3.25.

[$(F_5C_2)_2SbCH_2P(t\text{Bu})_2]_2\cdot$AuOTf (7):

A suspension of AgOTf (7.0 mg, 27 µmol) in *n*-hexane (1 mL) was treated with a solution of **6** (18 mg, 14 µmol) in *n*-hexane (1 mL). A colourless solid precipitated rapidly. This suspension was stirred overnight at ambient temperature, the solid was filtered and washed with *n*-hexane (3 × 1 mL). The volatile compounds were removed in vacuum, **7** was solved in CD_2Cl_2 and the solution was separated from the residue. After drying under vacuum, $[(F_5C_2)_2SbCH_2P(t\text{Bu})_2]_2\cdot$AuOTf (**7**, 17 mg) was obtained as a colourless solid. Single crystals suitable for X-ray diffraction were obtained from a saturated solution of **7** in C_6D_6.

Analytical data:

^{1}H NMR (600 MHz, CD_2Cl_2): δ [ppm] = 2.53 (m, 4H, C**H**$_2$), 1.46 (m, 36H, C(C**H**$_3$)$_3$).

^{13}C{^{1}H} NMR (151 MHz, CD_2Cl_2): δ [ppm] = 39.0 (m, **C**(CH$_3$)$_3$), 29.8 (m, C(**C**H$_3$)$_3$), 6.1 (m, **C**H$_2$).

^{13}C{^{19}F} NMR (151 MHz, CD_2Cl_2): δ [ppm] = 121.1 (s, O$_3$SC**F**$_3$), 119.8 (s, **C**F$_3$).

^{19}F NMR (565 MHz, CD_2Cl_2): δ [ppm] = −78.3 (s, O$_3$SC**F**$_3$), −81.9 (m, CF$_2$C**F**$_3$), −106.5/−108.8 (m, AB-spin system C**F**$_2$, broad).

^{31}P{^{1}H} NMR (243 MHz, CD_2Cl_2): δ [ppm] = 78.2 (s).

Elemental analysis calcd (%) for $C_{27}H_{40}AuF_{23}O_3P_2SSb_2$ (M_r = 1384.07): C 23.42, H 2.91, S 2.32; found: C 24.22, H 3.03, S 1.24.

NMR spectroscopy data

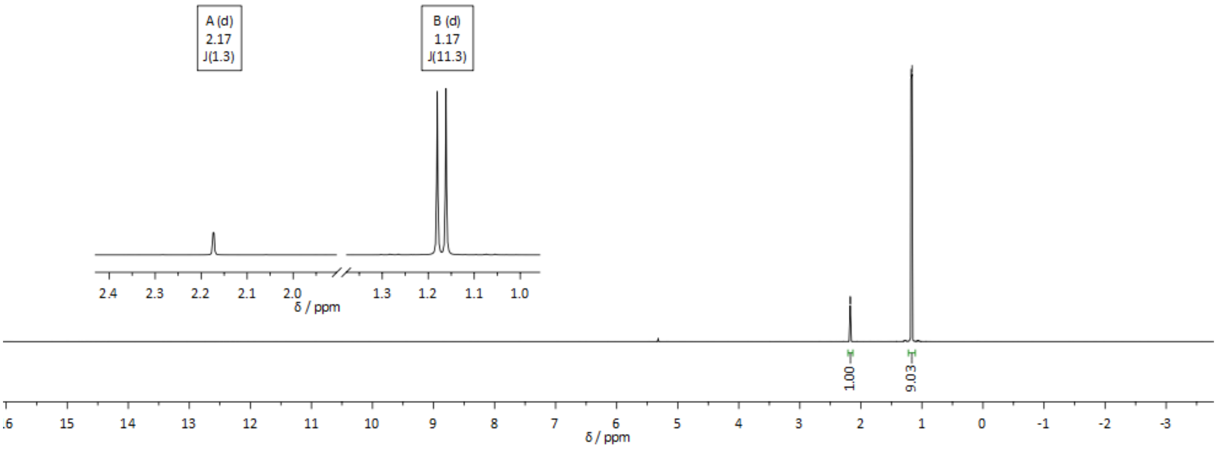

Figure S1 ^{1}H NMR spectrum of **1** in CD_2Cl_2.

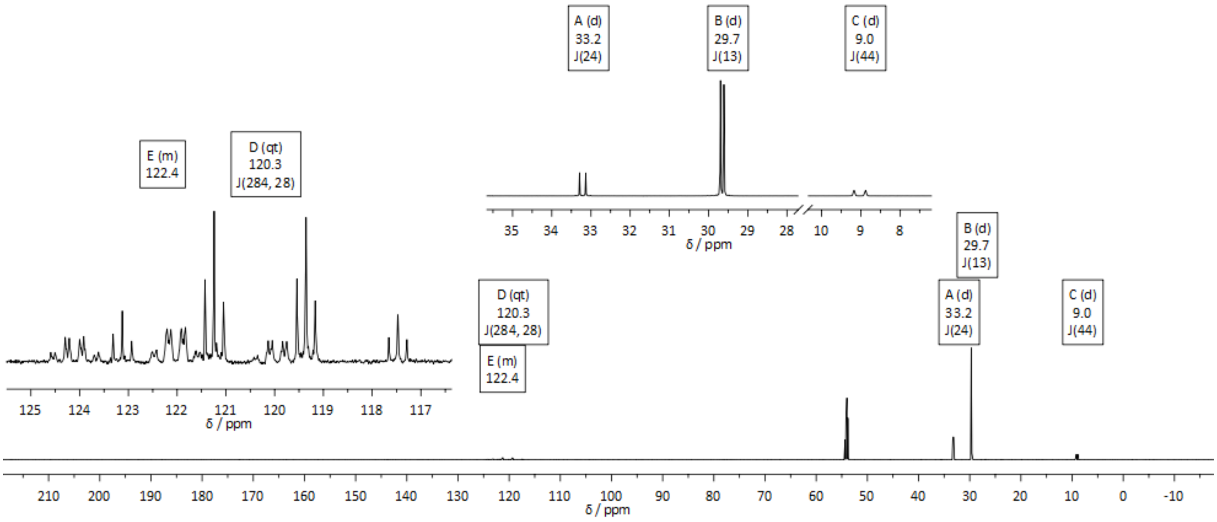

Figure S2 $^{13}C\{^{1}H\}$ NMR spectrum of **1** in CD_2Cl_2.

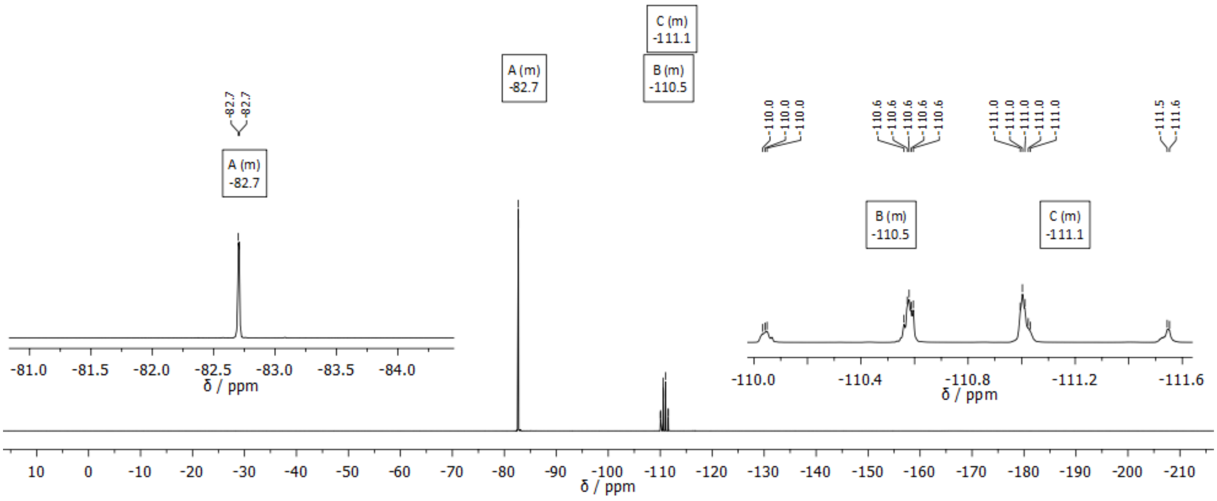

Figure S3 ^{19}F NMR spectrum of **1** in CD_2Cl_2.

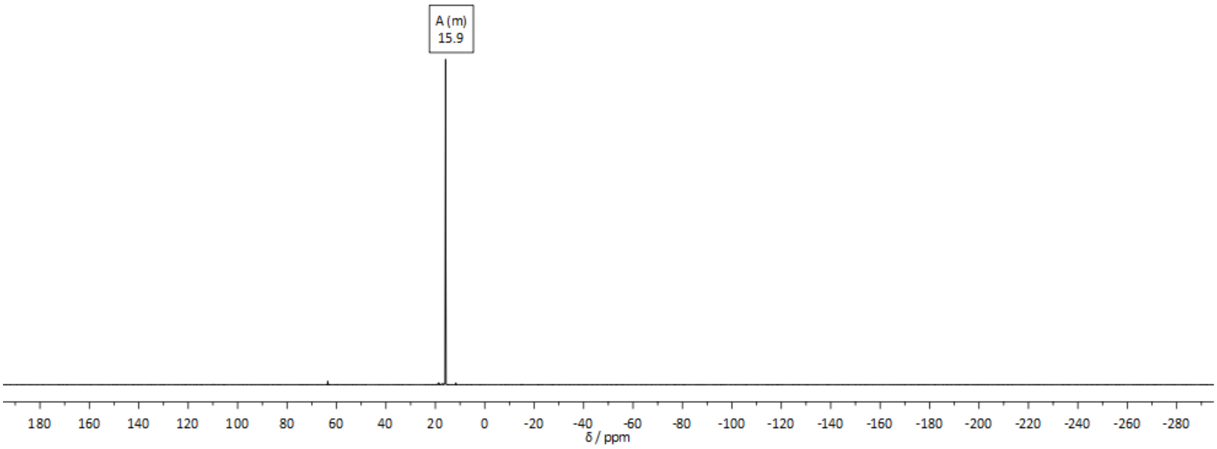

Figure S4 $^{31}P\{^{1}H\}$ NMR spectrum of **1** in CD_2Cl_2.

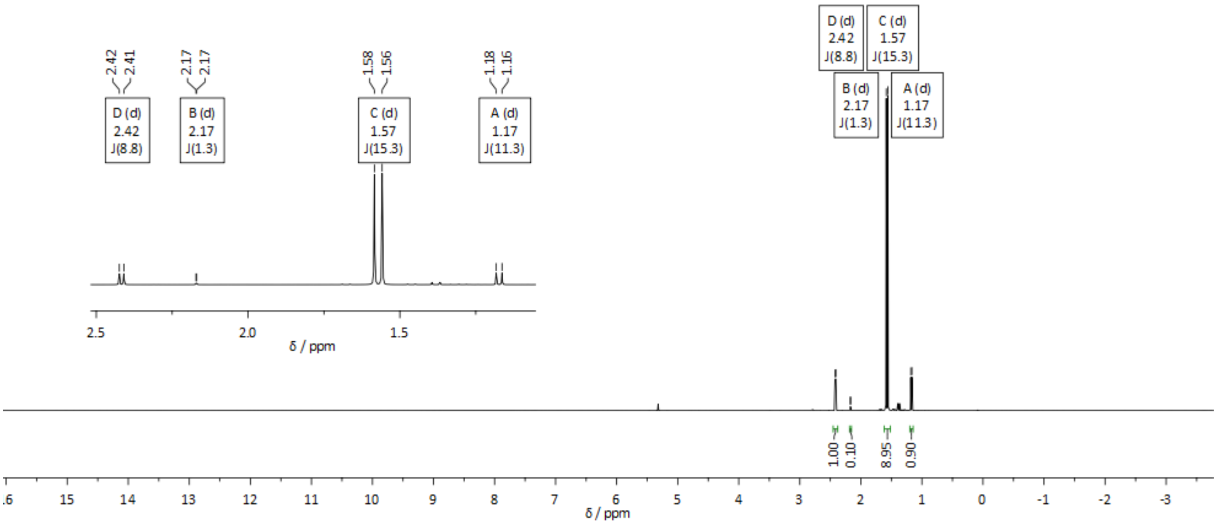

Figure S5 ^{1}H NMR spectrum of **2** in CD_2Cl_2.

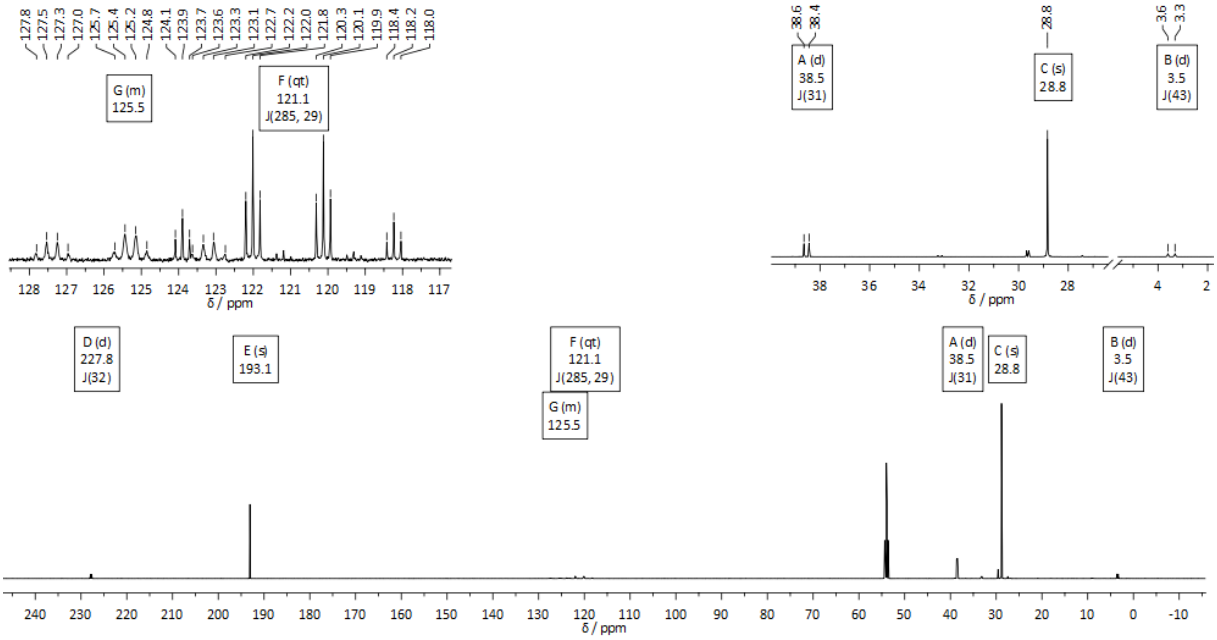

Figure S6 $^{13}C\{^{1}H\}$ NMR spectrum of **2** in CD_2Cl_2.

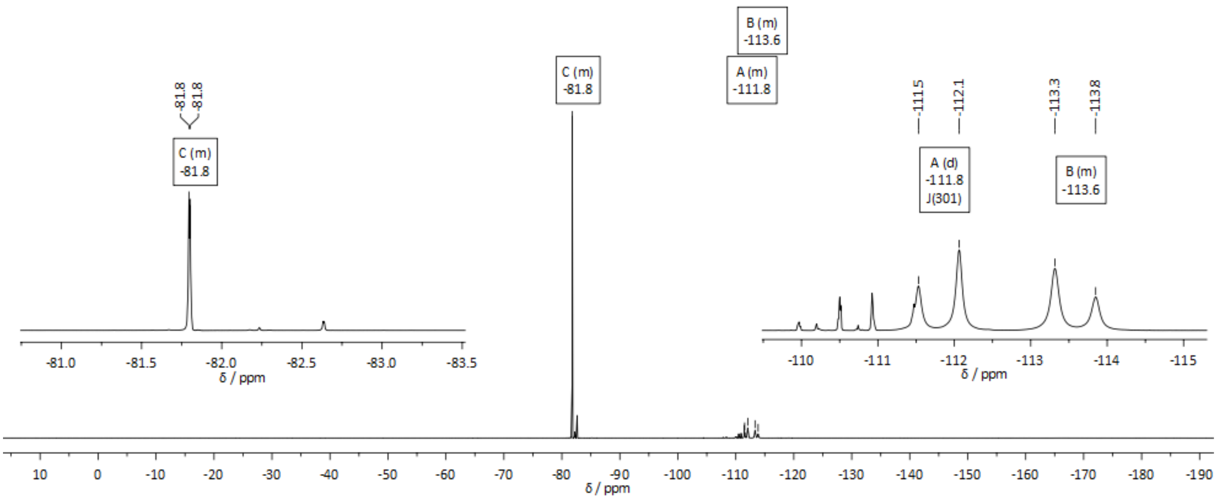

Figure S7 ^{19}F NMR spectrum of **2** in CD_2Cl_2.

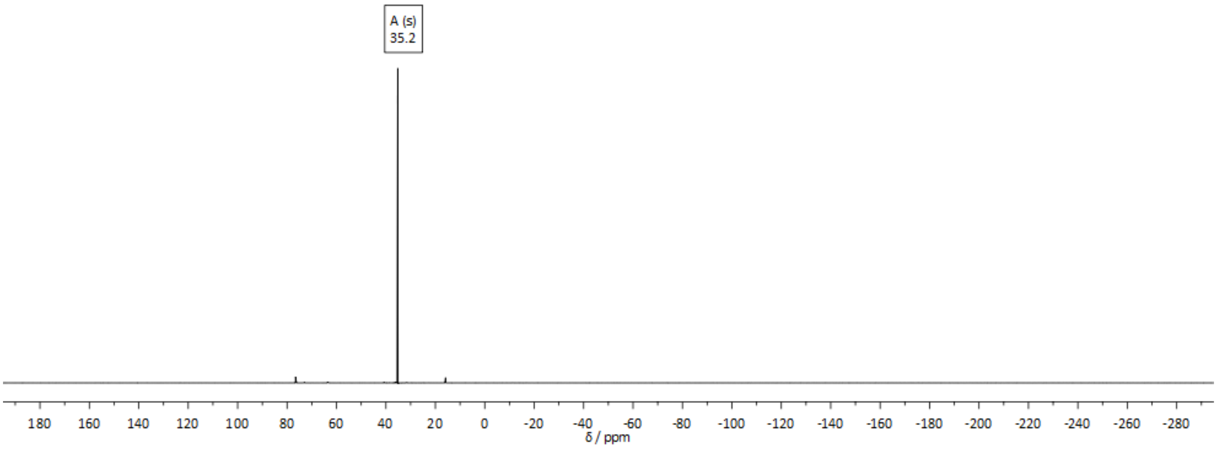

Figure S8 ^{31}P{^{1}H} NMR spectrum of **2** in CD_2Cl_2.

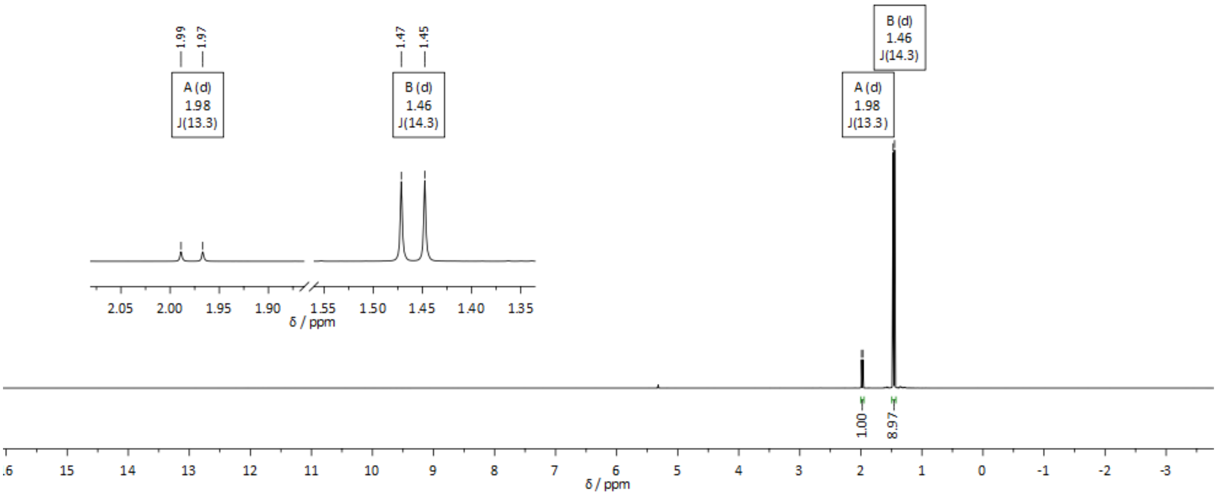

Figure S9 ^{1}H NMR spectrum of **3** in CD_2Cl_2.

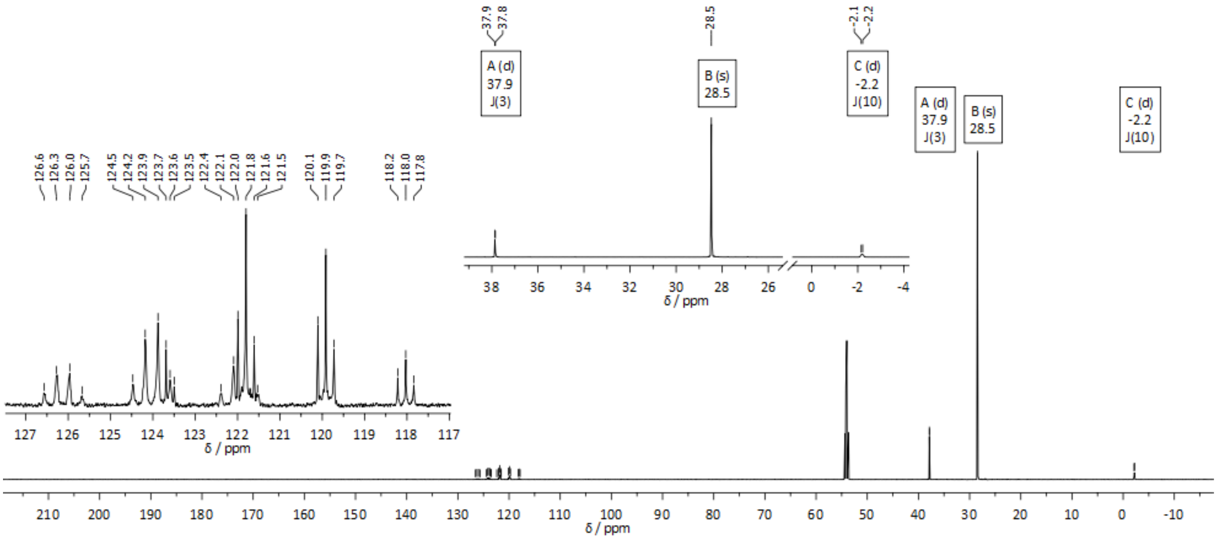

Figure S10 $^{13}C\{^{1}H\}$ NMR spectrum of **3** in CD_2Cl_2.

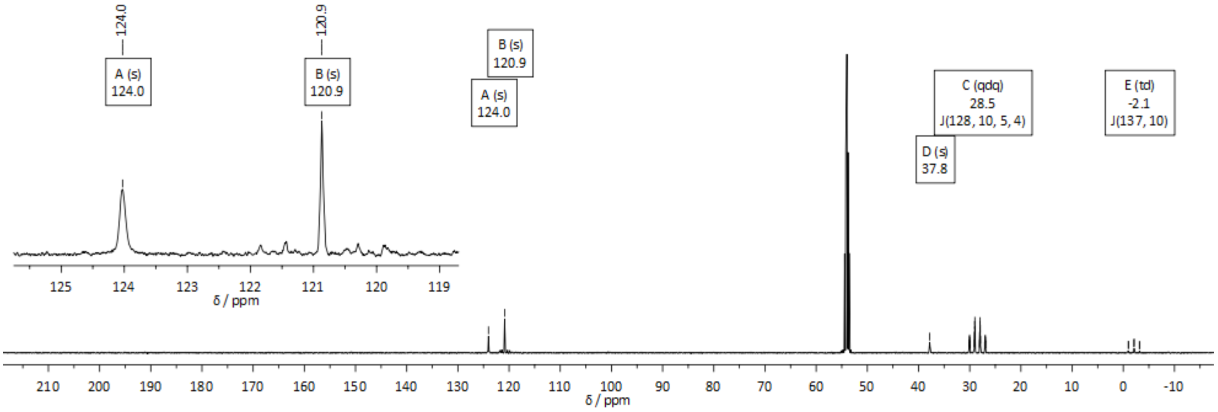

Figure S11 $^{13}C\{^{19}F\}$ NMR spectrum of **3** in CD_2Cl_2.

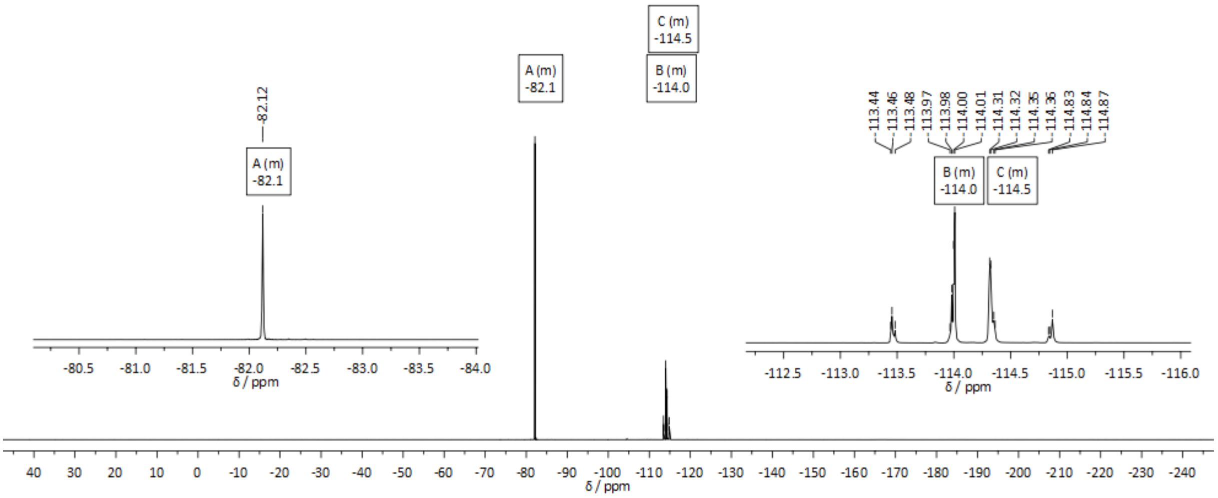

Figure S12 ^{19}F NMR spectrum of **3** in CD_2Cl_2.

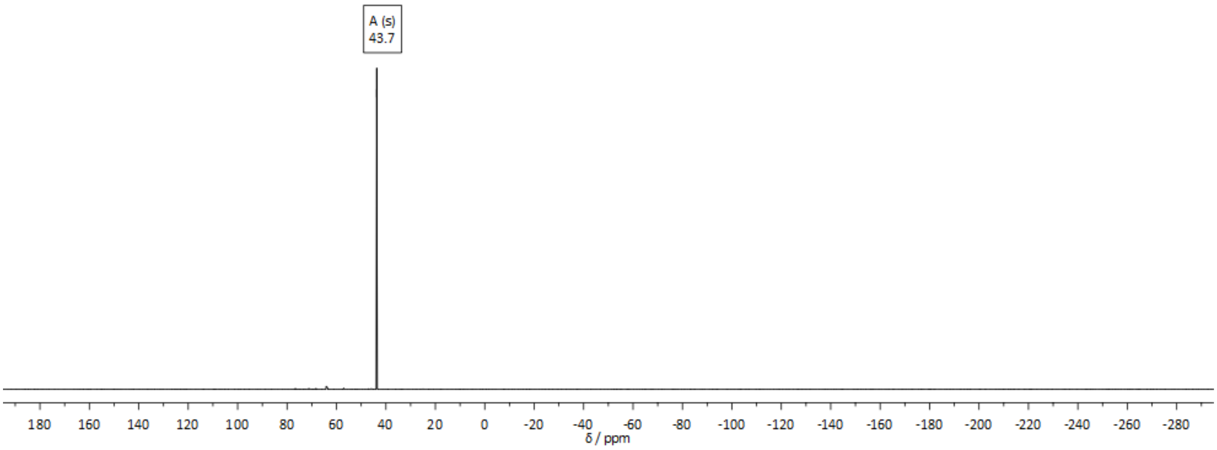

Figure S13 $^{31}P\{^{1}H\}$ NMR spectrum of **3** in CD_2Cl_2.

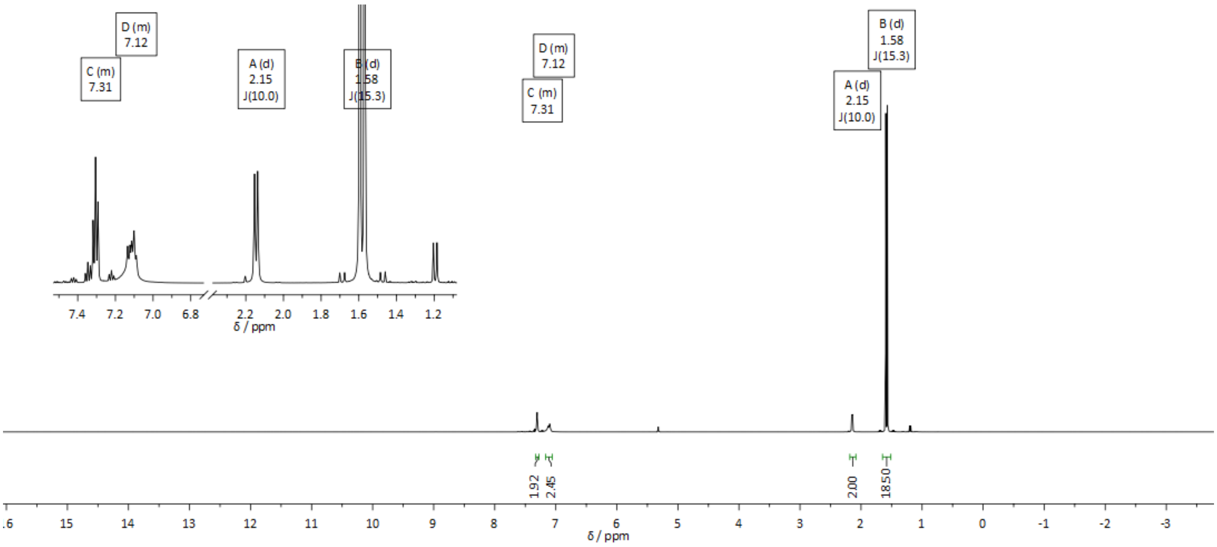

Figure S14 ^{1}H NMR spectrum of **4** in CD_2Cl_2.

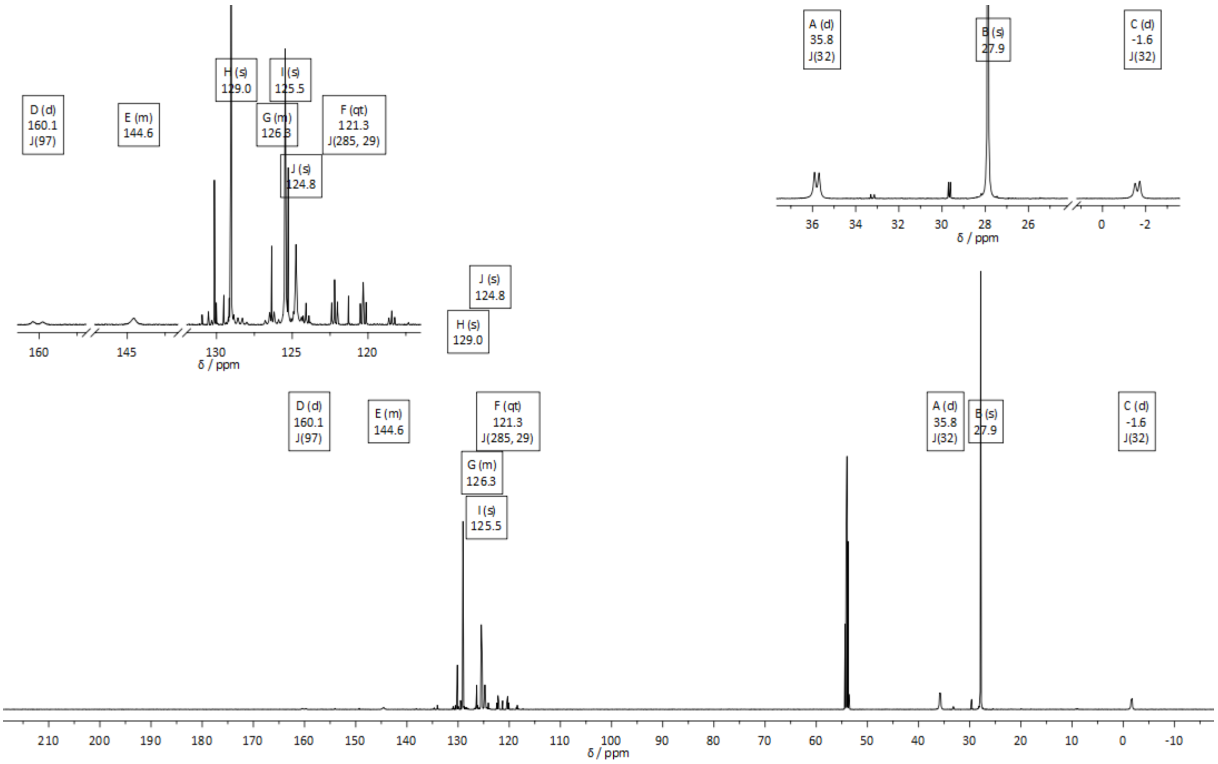

Figure S15 $^{13}C\{^{1}H\}$ NMR spectrum of **4** in CD_2Cl_2.

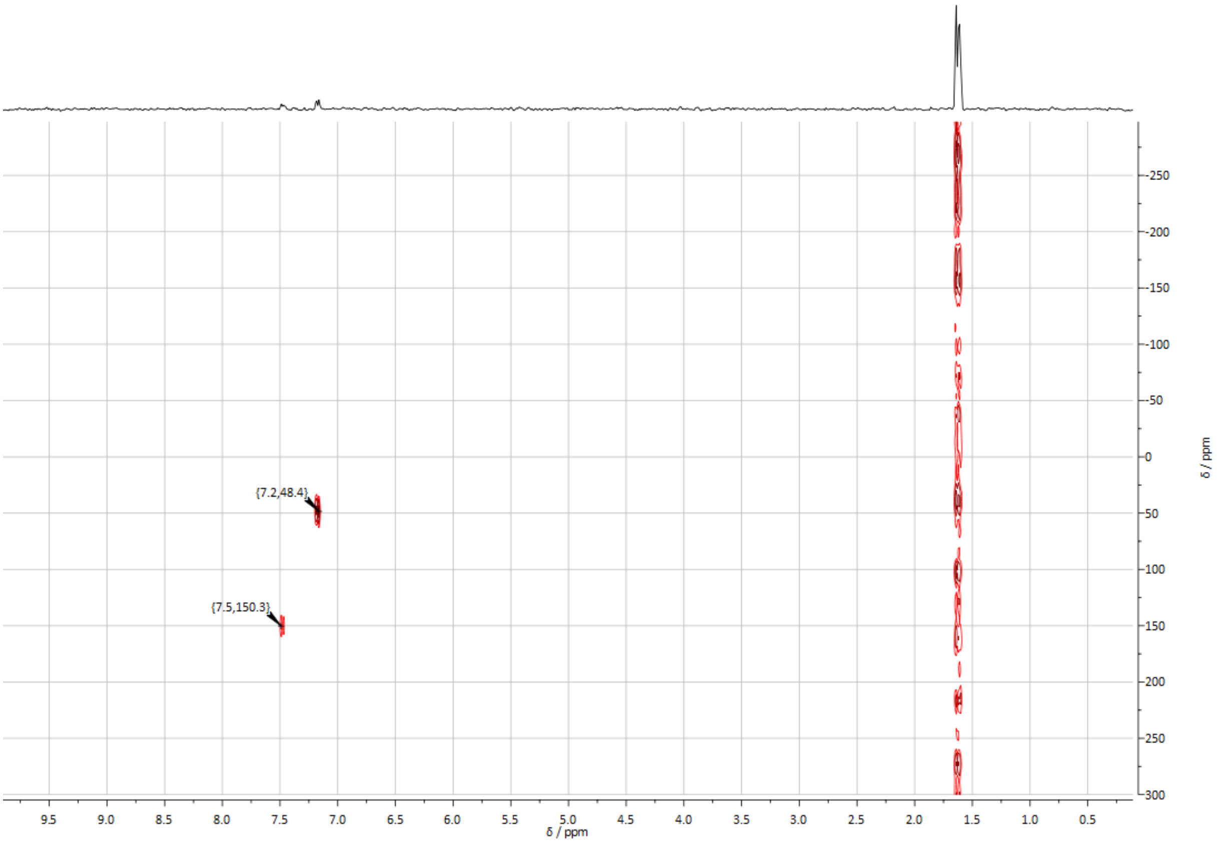

Figure S16 ^{15}N ^{1}H HMBC NMR spectrum of **4** in CD_2Cl_2

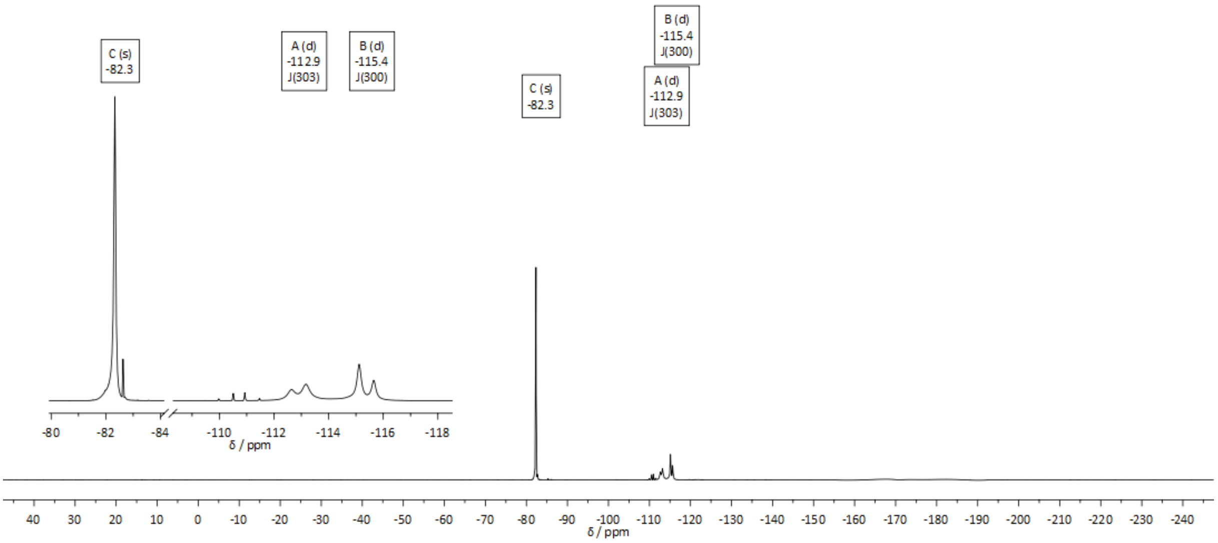

Figure S17 ^{19}F NMR spectrum of **4** in CD_2Cl_2.

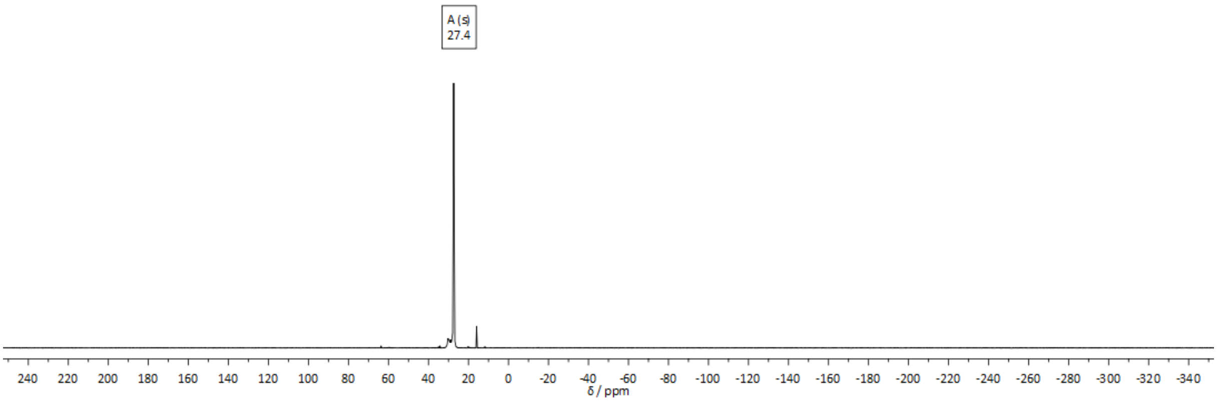

Figure S18 $^{31}P\{^1H\}$ NMR spectrum of **4** in CD_2Cl_2.

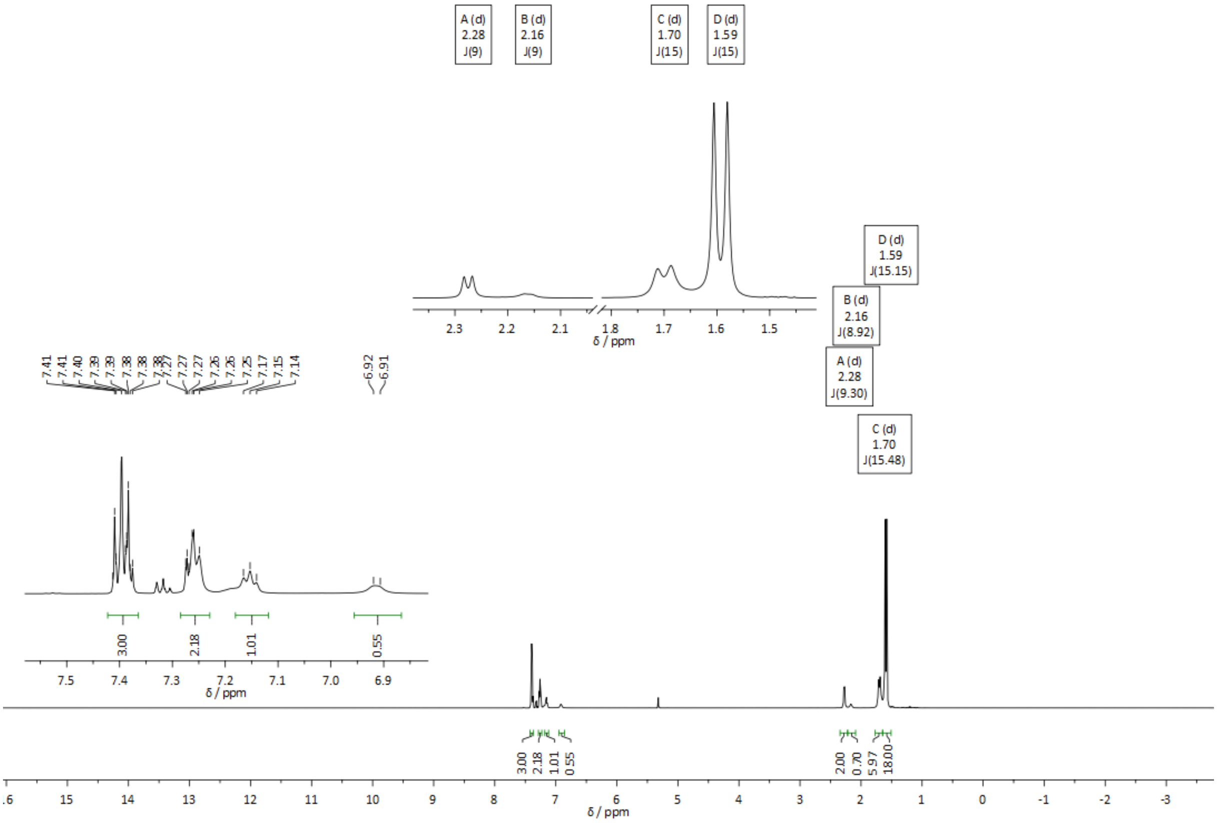

Figure S19 ^{1}H NMR spectrum of **5** in CD_2Cl_2.

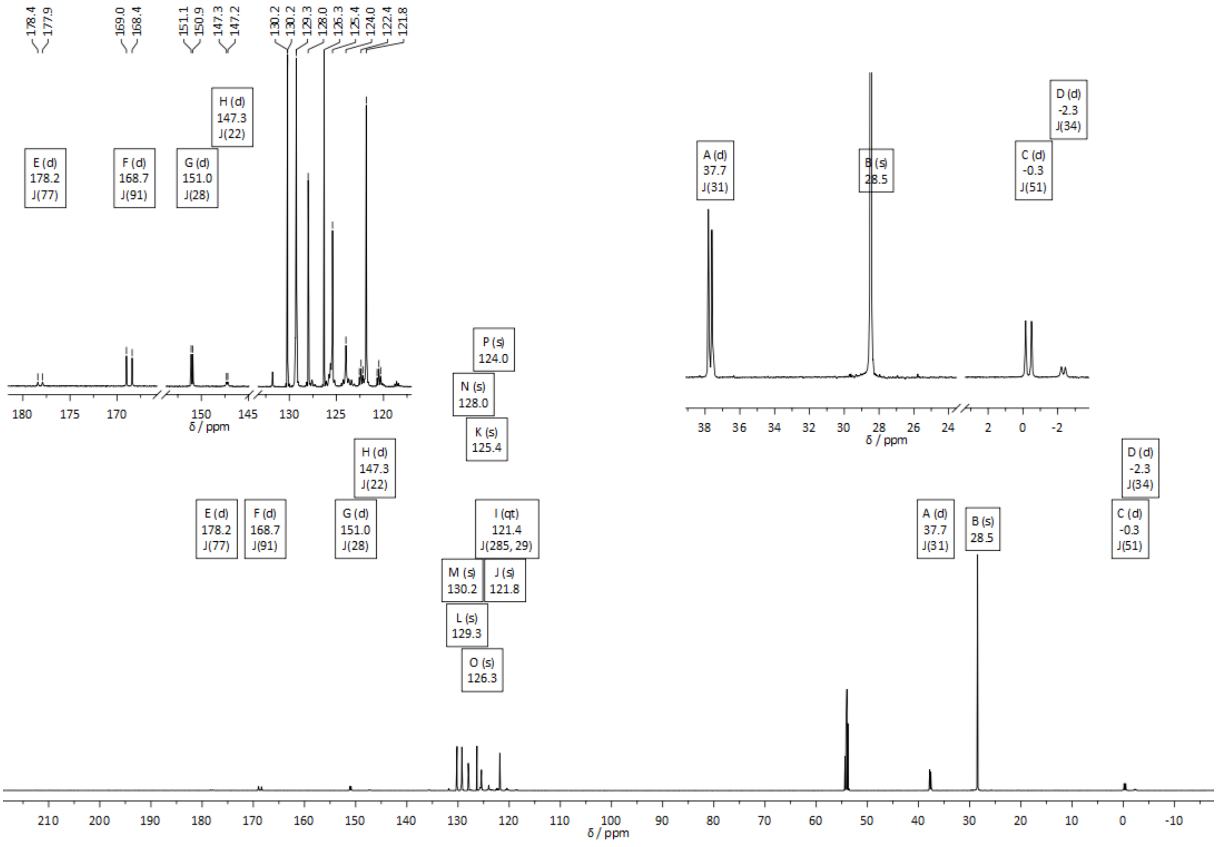

Figure S20 $^{13}C\{^{1}H\}$ NMR spectrum of **5** in CD_2Cl_2.

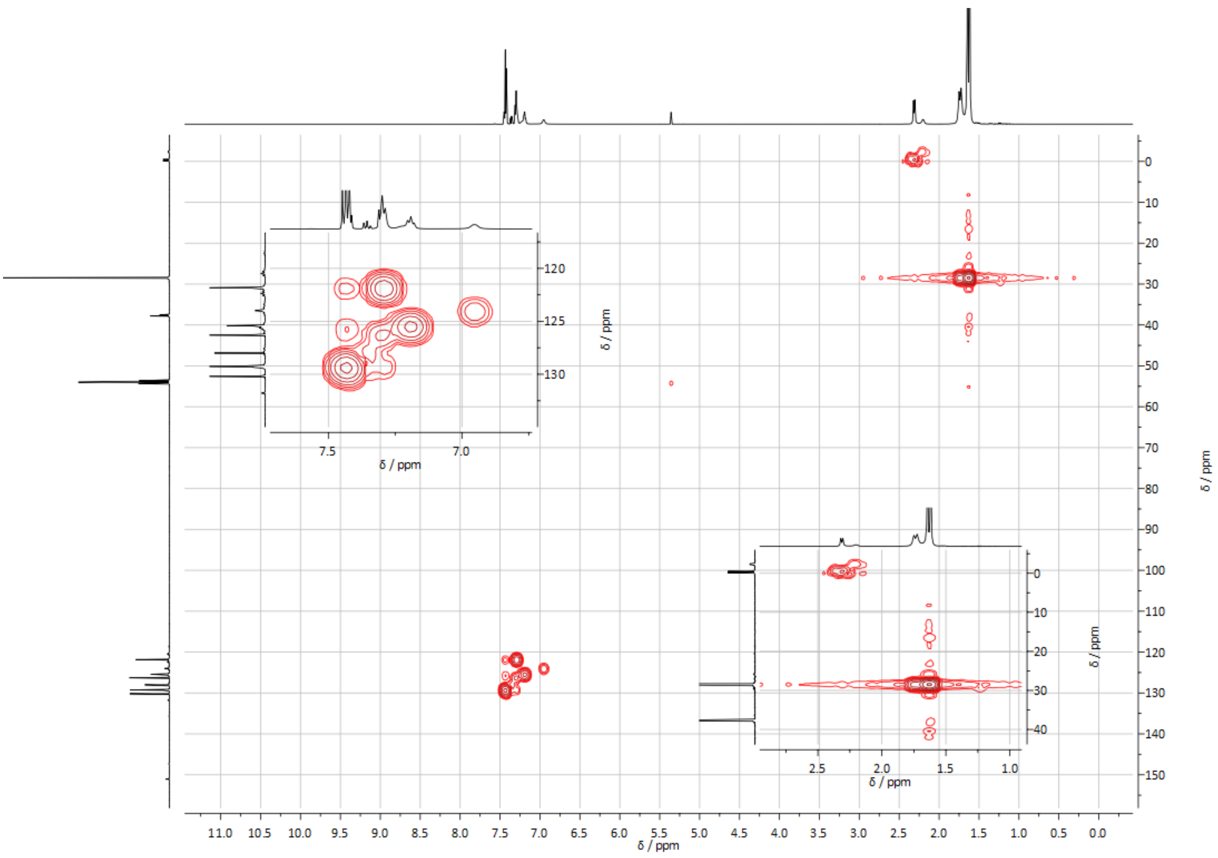

Figure S21 ^{13}C ^{1}H HMQC NMR spectrum of **5** in CD_2Cl_2.

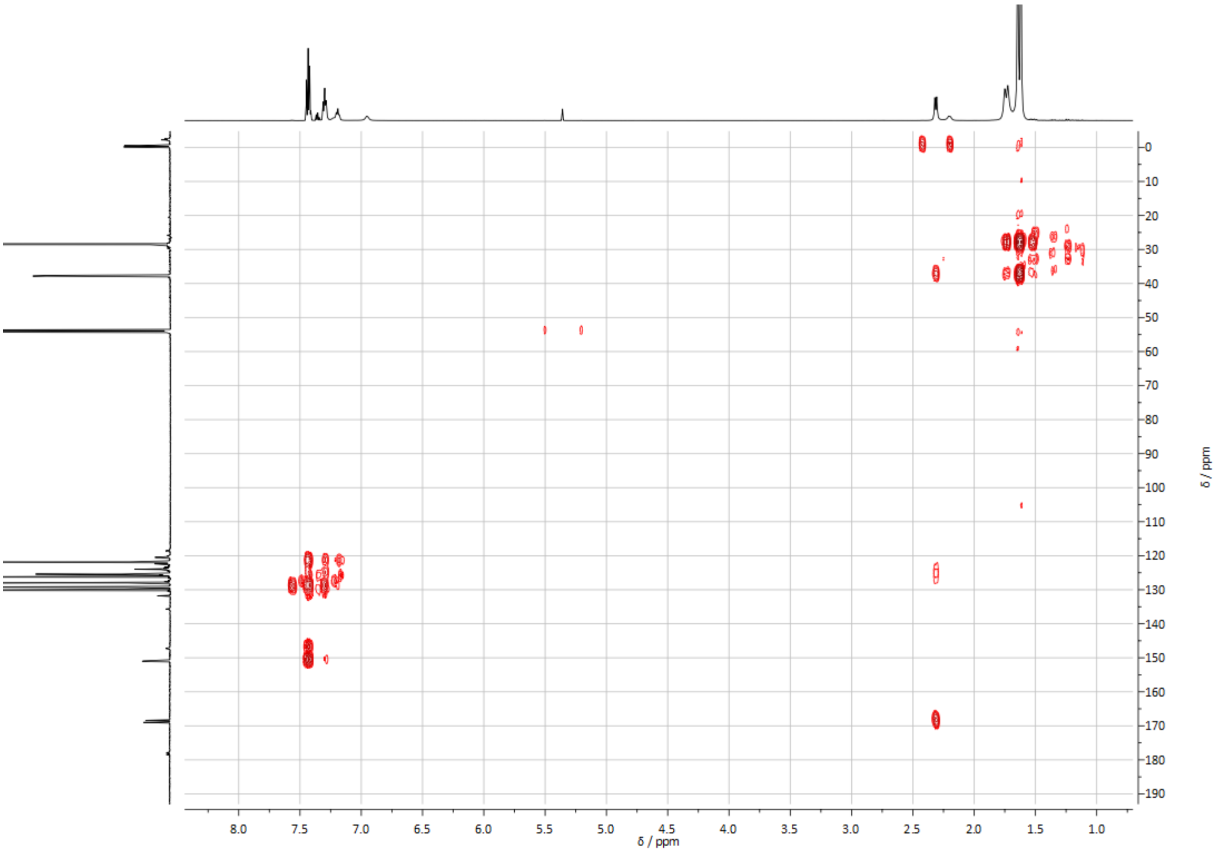

Figure S22 ^{13}C ^{1}H HMQC NMR spectrum of **5** in CD_2Cl_2.

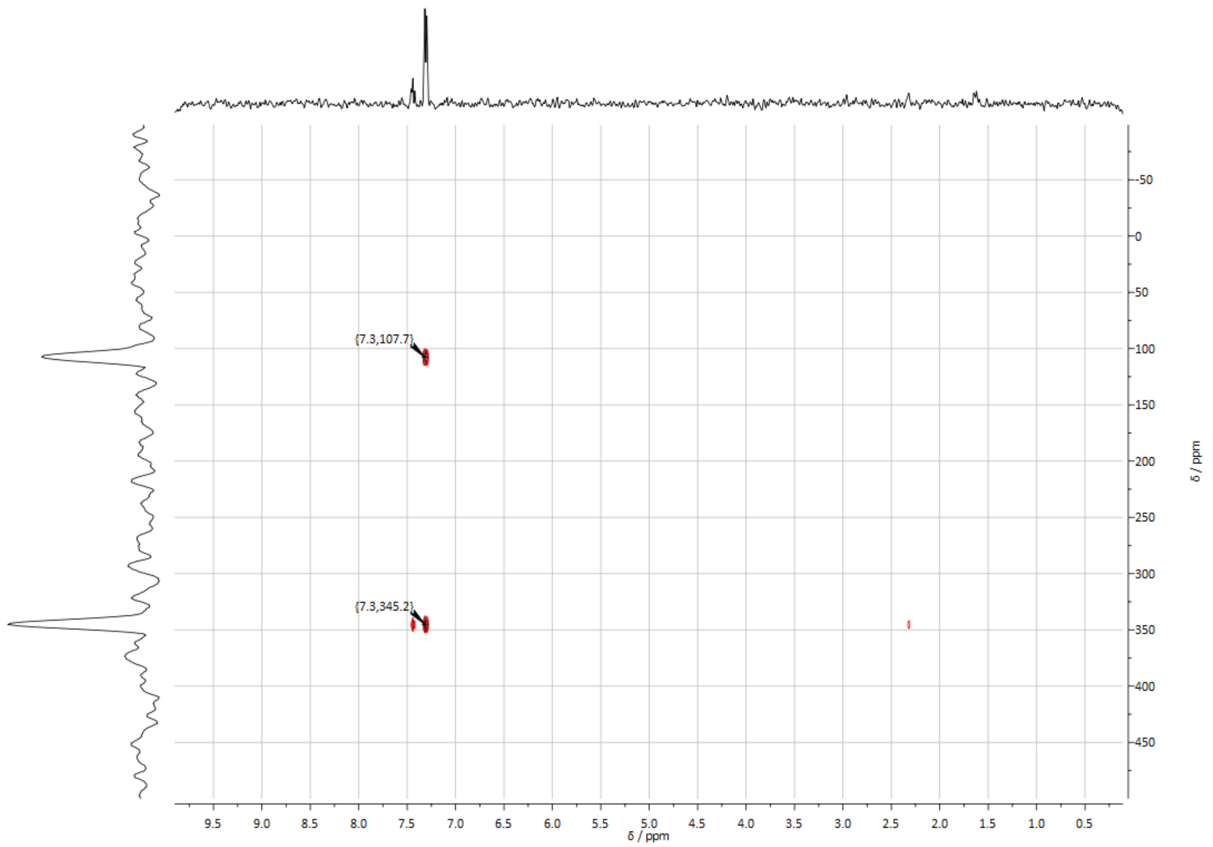

Figure S23 ^{15}N ^{1}H HMBC NMR spectrum of **5** in CD_2Cl_2.

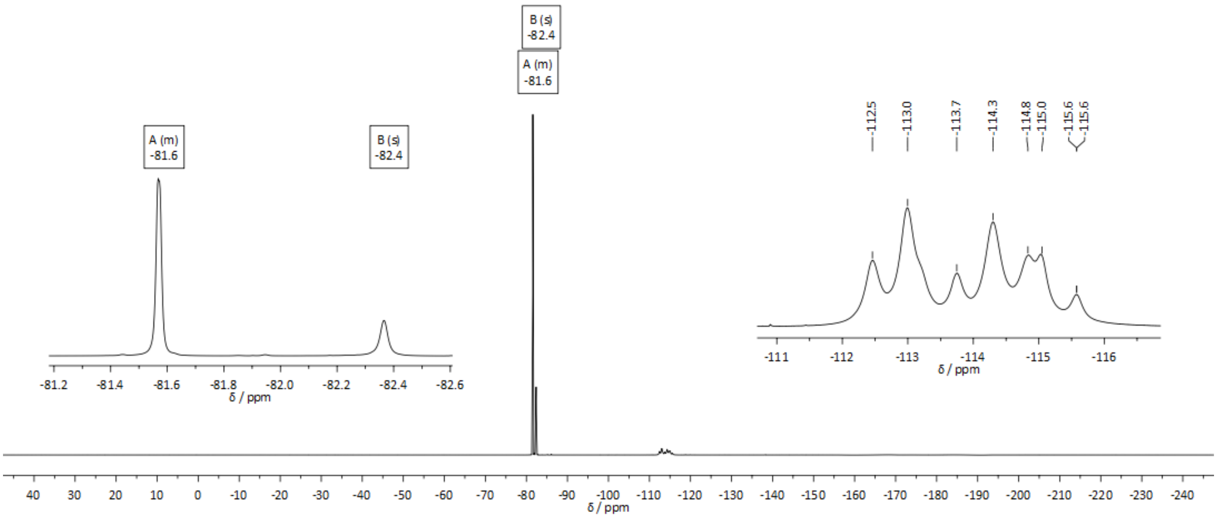

Figure S24 ^{19}F NMR spectrum of **5** in CD_2Cl_2.

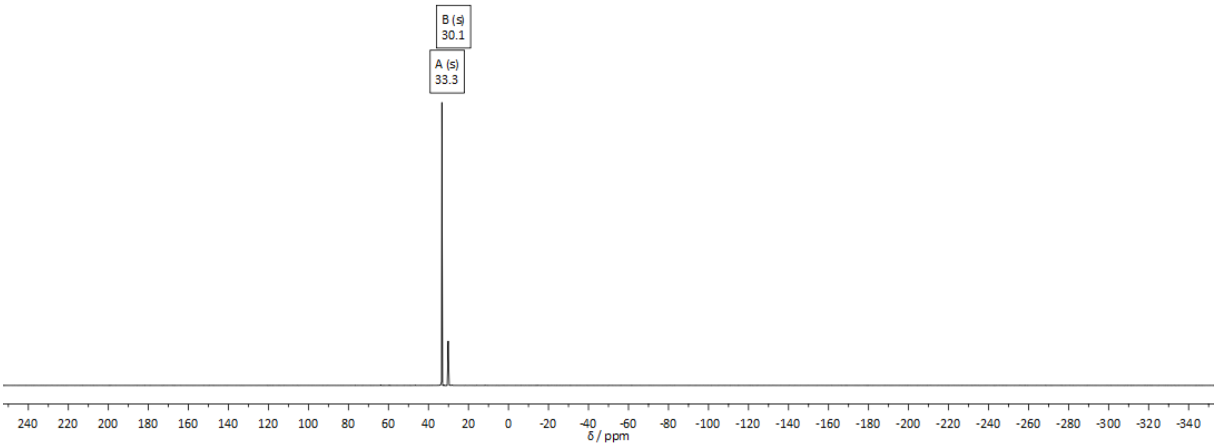

Figure S25 $^{31}P\{^{1}H\}$ NMR spectrum of **5** in CD_2Cl_2.

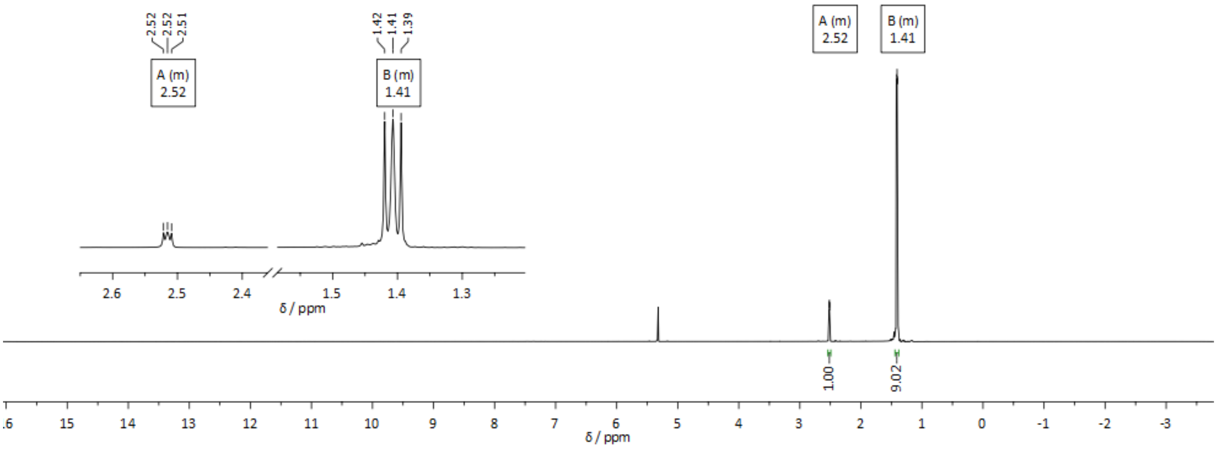

Figure S26 ^{1}H NMR spectrum of **6** in CD_2Cl_2.

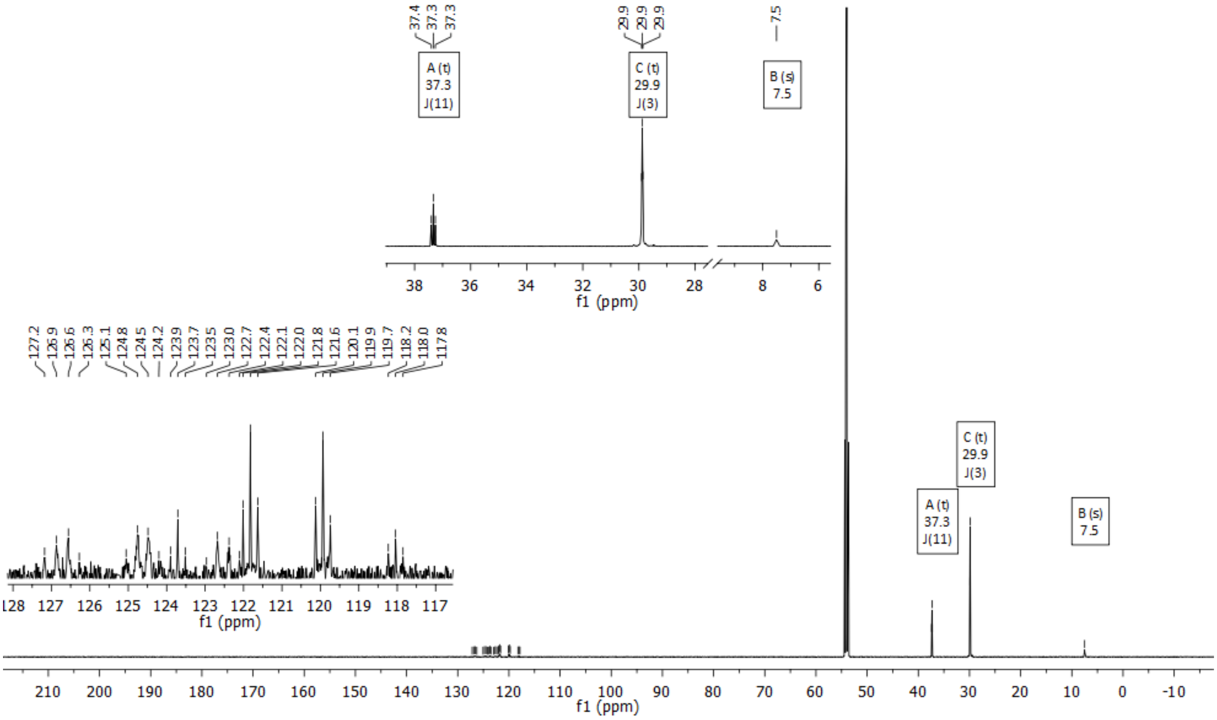

Figure S27 $^{13}C\{^{1}H\}$ NMR spectrum of **6** in CD_2Cl_2.

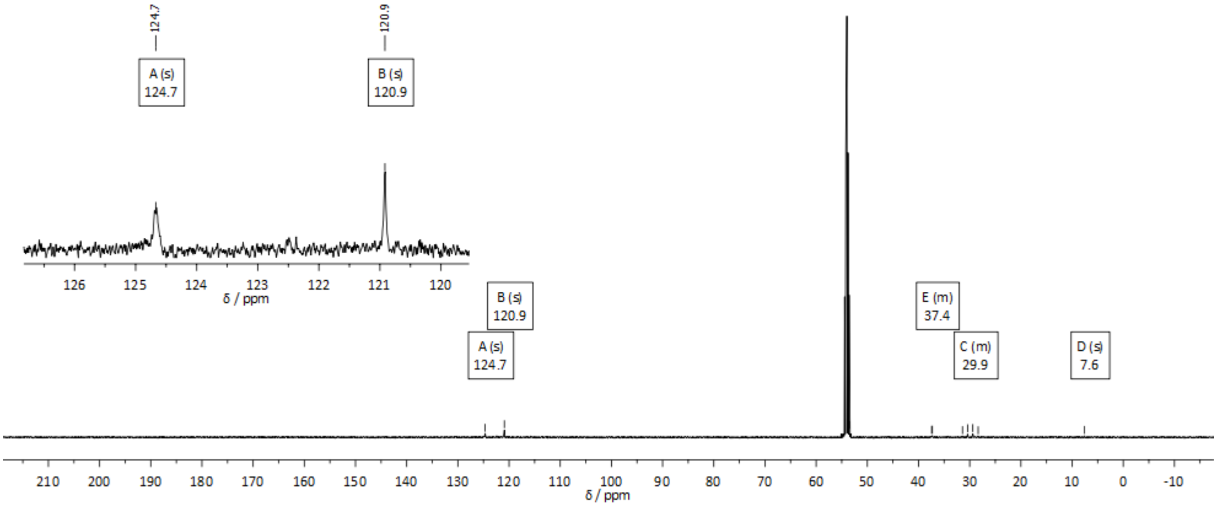

Figure S28 $^{13}C\{^{19}F\}$ NMR spectrum of **6** in CD_2Cl_2.

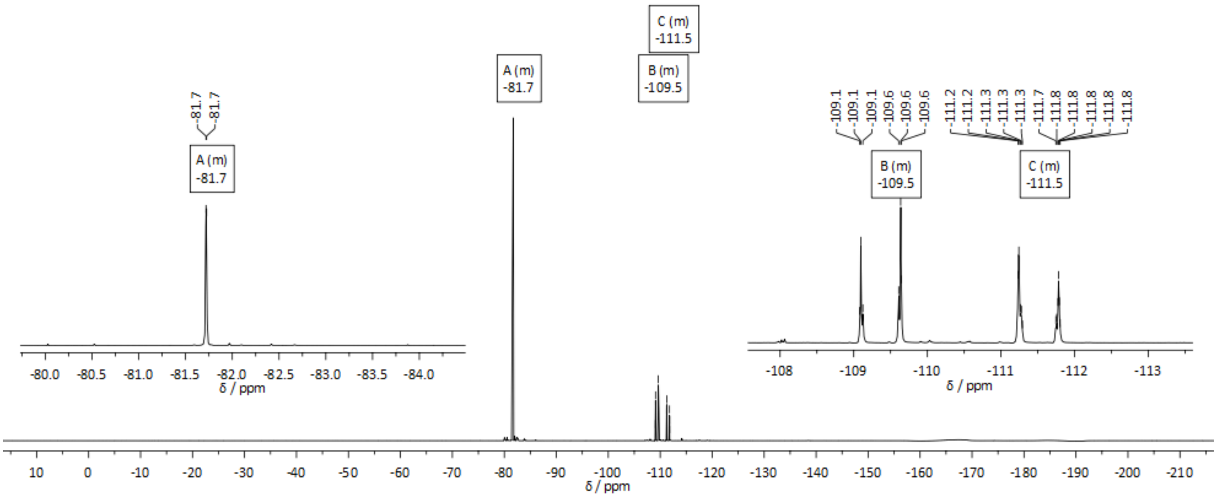

Figure S29 ^{19}F NMR spectrum of **6** in CD_2Cl_2.

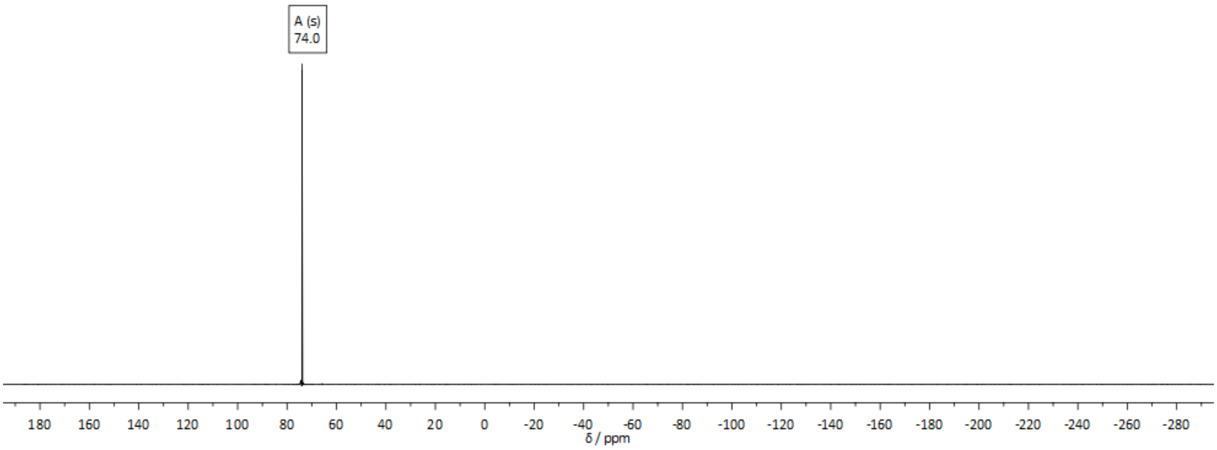

Figure S30 $^{31}P\{^{1}H\}$ NMR spectrum of **6** in CD_2Cl_2.

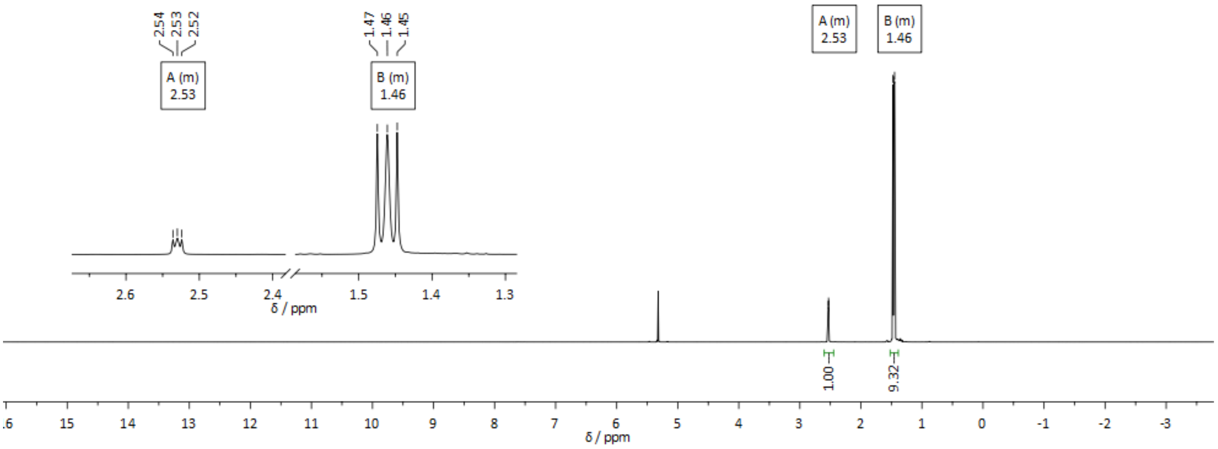

Figure S31 ^{1}H NMR spectrum of **7** in CD_2Cl_2.

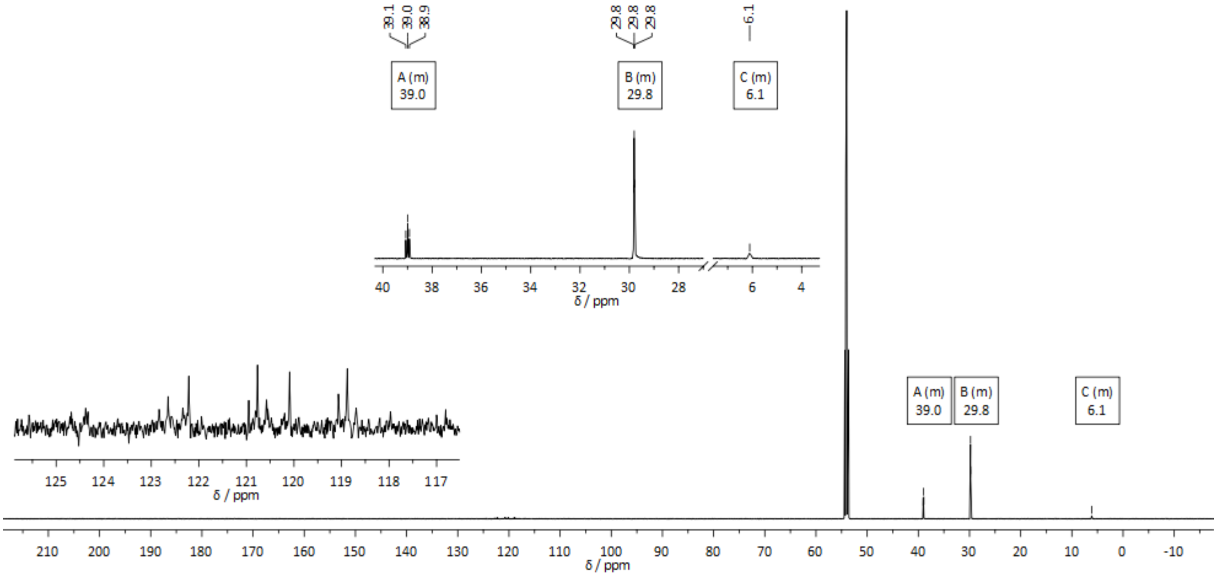

Figure S32 $^{13}C\{^{1}H\}$ NMR spectrum of **7** in CD_2Cl_2.

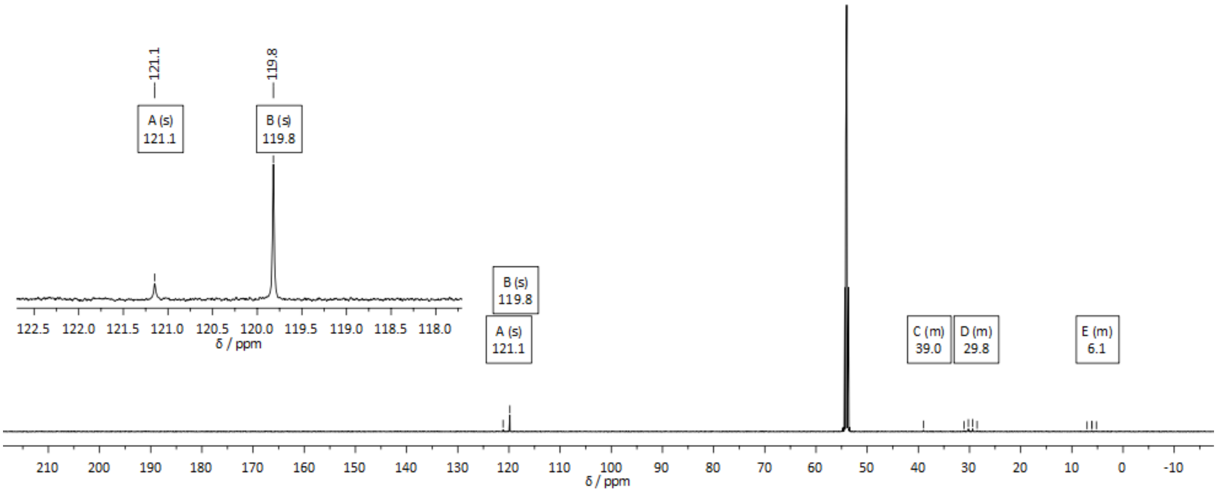

Figure S33 $^{13}C\{^{19}F\}$ NMR spectrum of **7** in CD_2Cl_2.

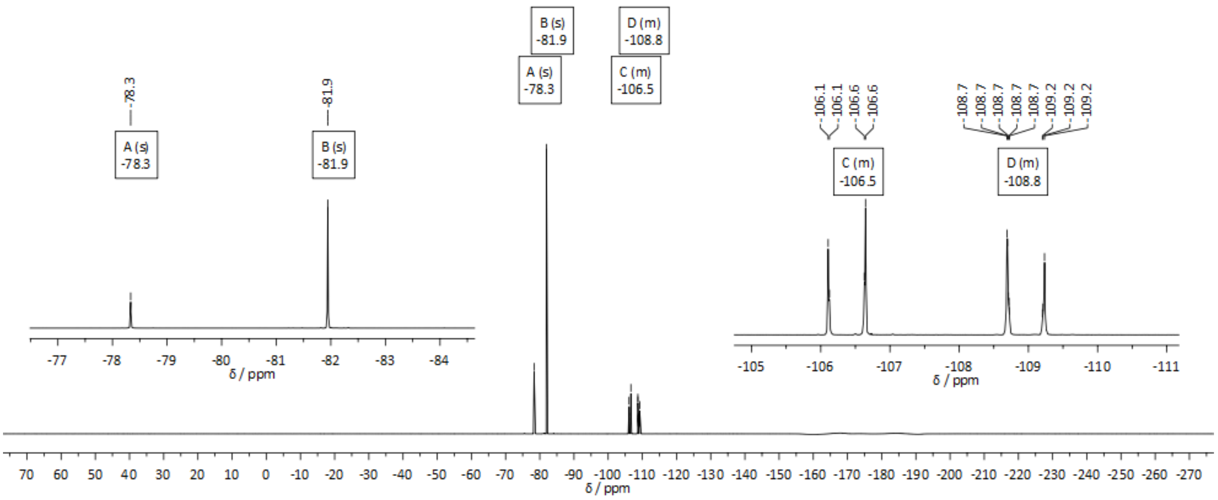

Figure S34 ^{19}F NMR spectrum of **7** in CD_2Cl_2.

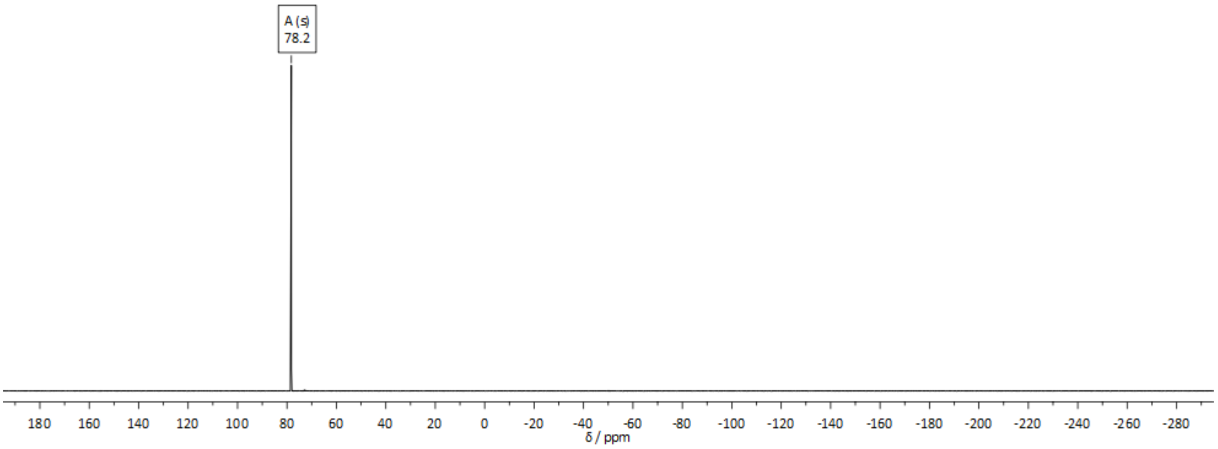

Figure S35 $^{31}P\{^{1}H\}$ NMR spectrum of **7** in CD_2Cl_2.

Supplemental NMR studies

In the following, supplemental NMR studies are listed to examine several aspects.

We performed Lewis acidity tests with the Gutmann-Beckett method[4] with $OPEt_3$ and the modified method for soft Lewis acids with $SePMe_3$ presented by Lichtenberg.[5] After addition of $OPEt_3$ to **1** (1:1 eq. and 1:5 eq.: $OPEt_3$:**1**), we do not see any variation of the $^{31}P\{^1H\}$ NMR chemical shifts of **1** (15.9 ppm) and $OPEt_3$ (50.4 ppm), respectively.

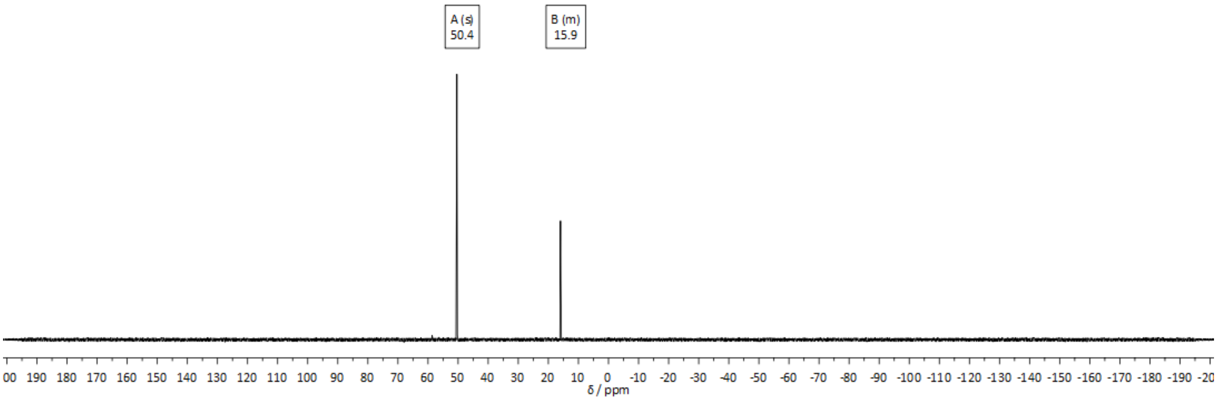

Figure S36 Gutmann-Beckett test in CD_2Cl_2 with 1 eq. $OPEt_3$ and 1 eq. **1**.

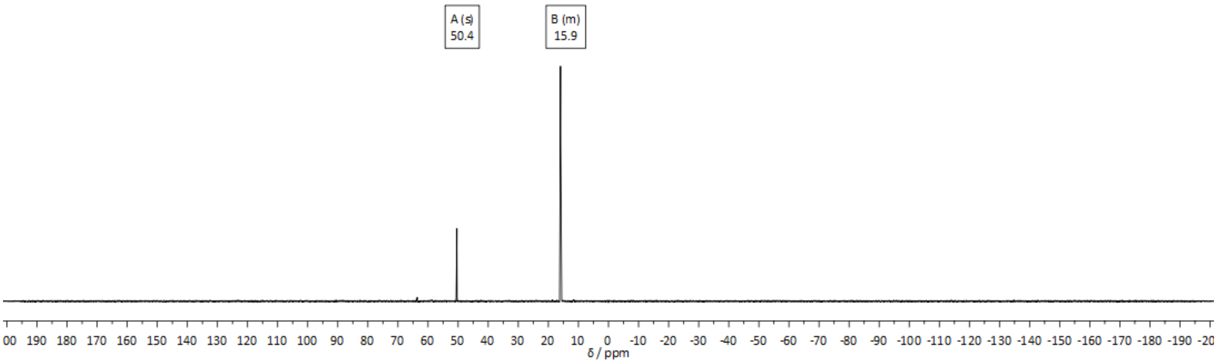

Figure S37 Gutmann-Beckett test in CD_2Cl_2 with 1 eq. $OPEt_3$ and 5 eq. **1**.

With the softer Lewis base $SePMe_3$ (1:1 eq. and 1:5 eq.; $SePMe_3$:**1**), we observe a selenium transfer from $SePMe_3$ (8.8 ppm) to **1** (15.9 ppm) to give $(F_5C_2)_2SbCH_2(Se)P(tBu)_2$ (73.0 ppm) and PMe_3 (−61.6 ppm).

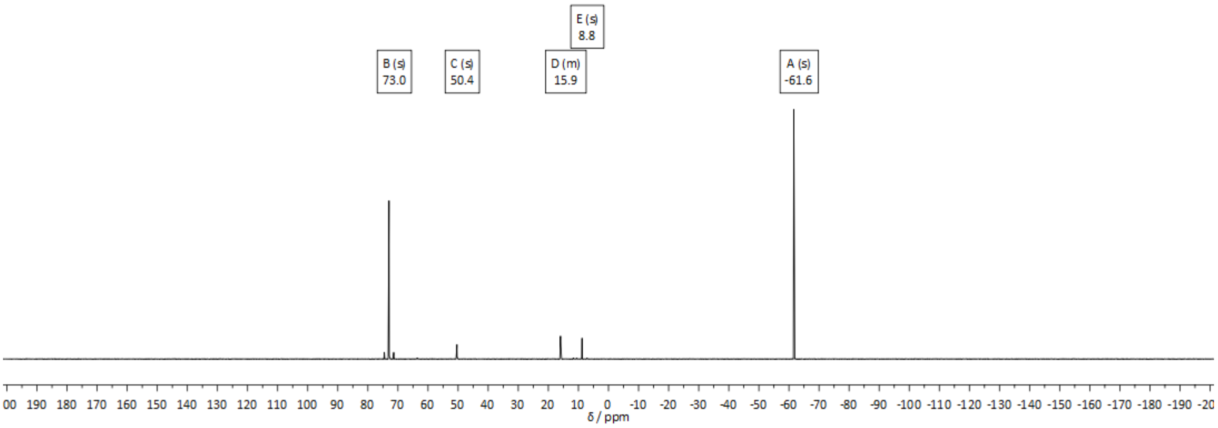

Figure S38 Modified Gutmann-Beckett test in CD_2Cl_2 with 1 eq. $SePMe_3$ and 1 eq. **1**. ($OPEt_3$ is present because of its previous addition in a Gutmann-Beckett test).

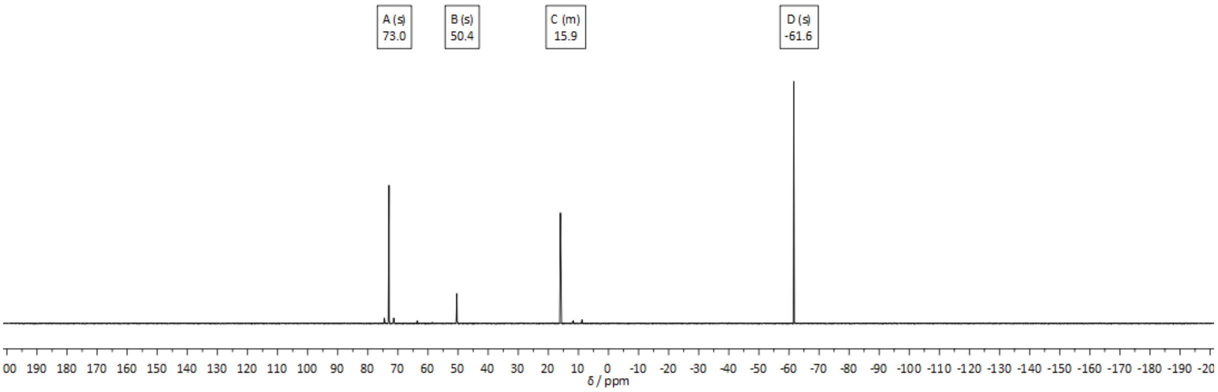

Figure S39 Modified Gutmann-Beckett test in CD_2Cl_2 with 1 eq. $SePMe_3$ and 5 eq. **1**. ($OPEt_3$ is present because of its previous addition in a Gutmann-Beckett test).

In Figures S40–S42 are reactivity tests of di-*tert*-butylmethylphosphane and the substrates CS_2, SO_2, PhNCO and PhNCS depicted. SO_2 and PhNCO do not show product formation, whereby CS_2 and PhNCS do. Additional VT-NMR studies show a temperature dependent equilibrium for the reactant/product mixtures, respectively.

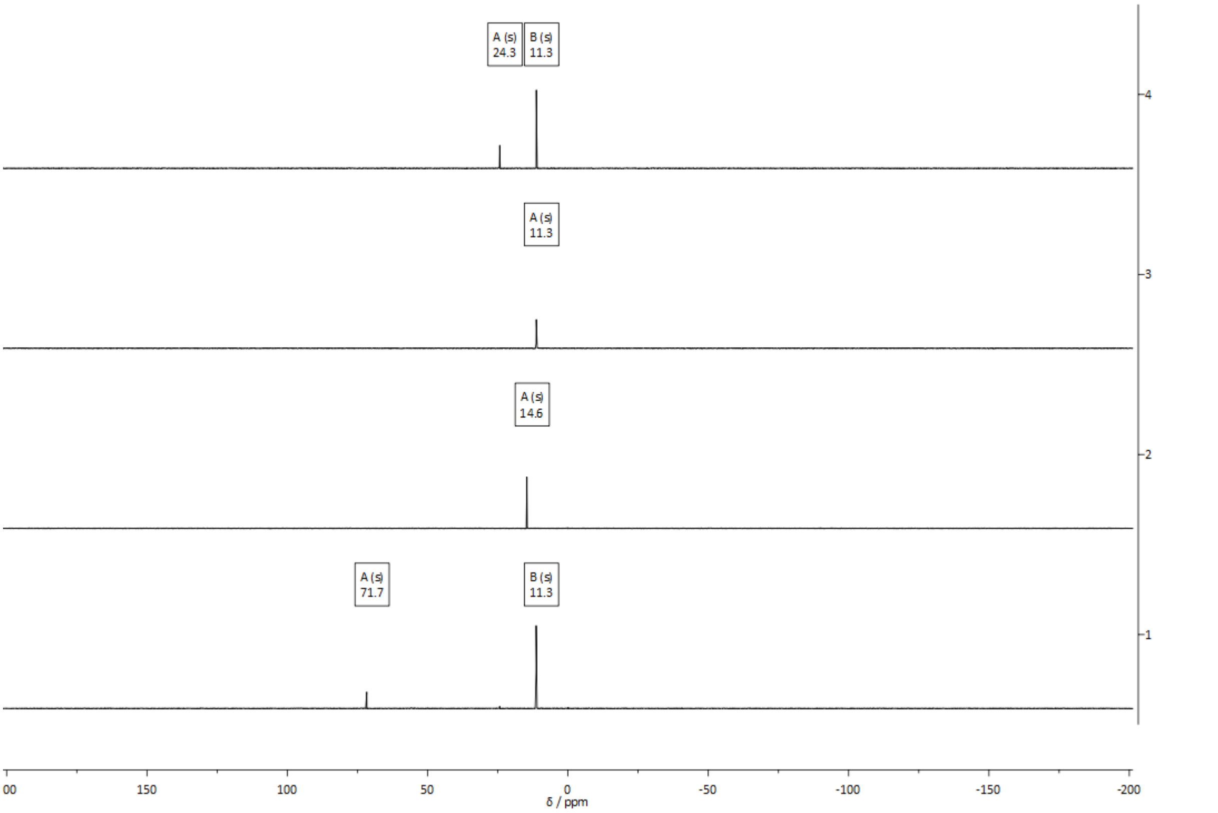

Figure S40 $^{31}P\{^1H\}$ NMR spectra of mixtures of $MeP(tBu)_2$ (11.3 ppm) and CS_2 (1), SO_2 (2), PhNCO (3) and PhNCS (4) in C_6D_6. The slightly shifted reactant peak in 2, may be because of the high excess of SO_2 in the NMR tube (>1 bar SO_2).

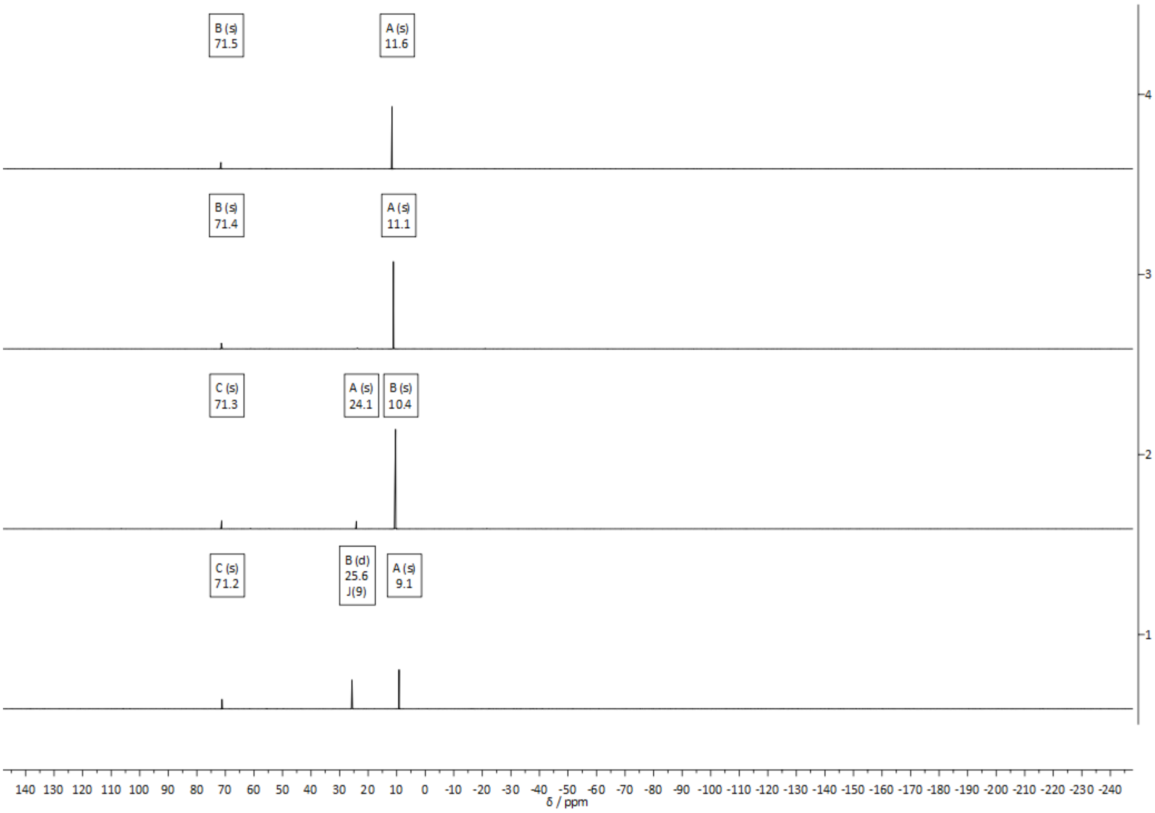

Figure S41 $^{31}P\{^1H\}$ NMR spectra of a mixture of MeP(*t*Bu)$_2$ (11.1 ppm at 298 K) and CS_2 at several temperatures (1: 233 K, 2:273 K, 3: 298 K, 4: 313 K) in toluene-d_8.

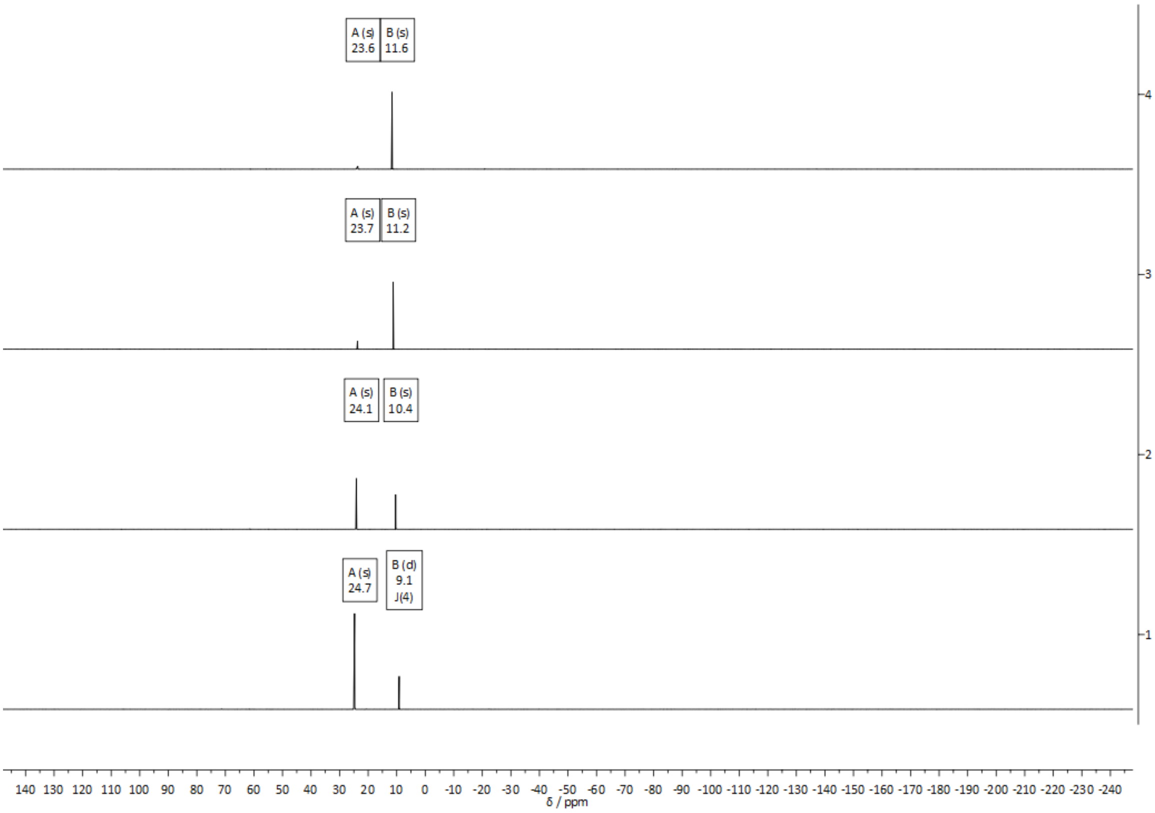

Figure S42 $^{31}P\{^1H\}$ NMR spectra of a mixture of MeP(*t*Bu)$_2$ (11.2 ppm at 298 K) and PhNCS at several temperatures (1: 233 K, 2:273 K, 3: 298 K, 4: 313 K) in toluene-d_8.

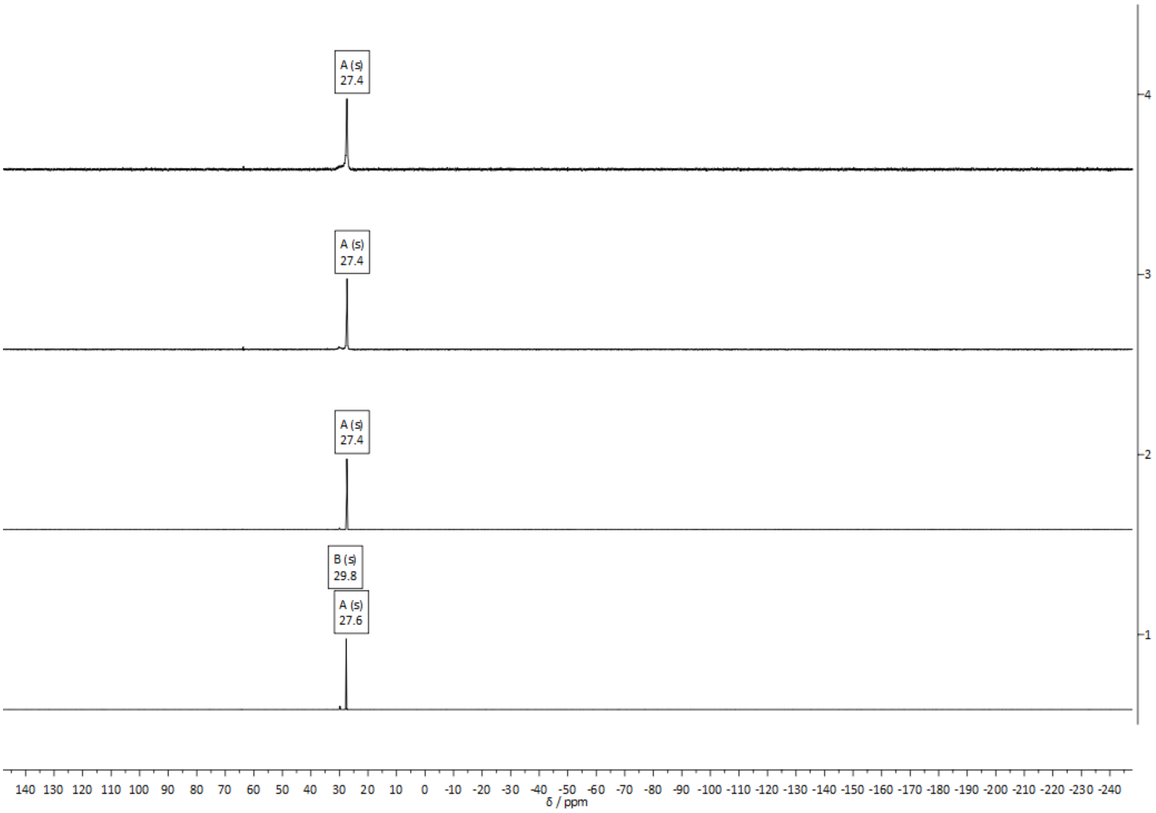

Figure S43 $^{31}P\{^1H\}$ NMR spectra of **4** at several temperatures (1: 233 K, 2:273 K, 3: 298 K, 4: 318 K) in CD_2Cl_2.

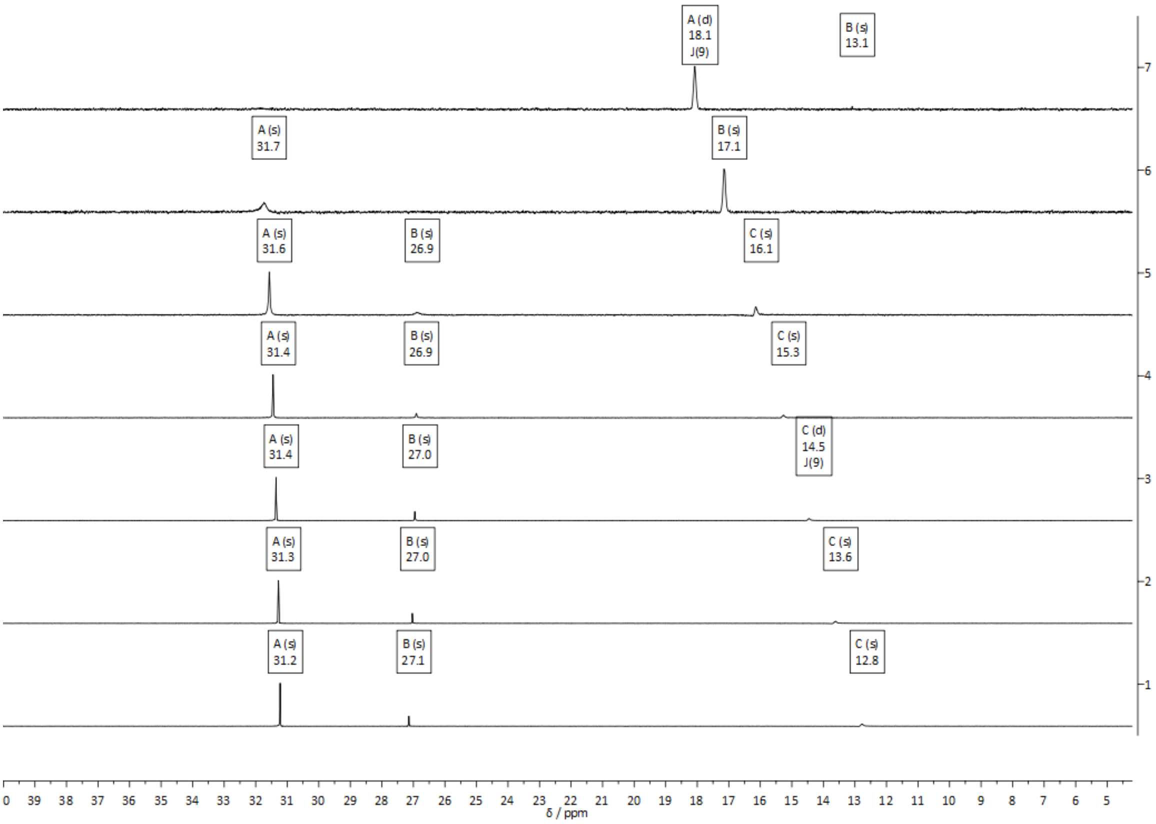

Figure S44 $^{31}P\{^1H\}$ NMR spectra of **5a/b** at several temperatures (1: 233 K, 2:253 K, 3: 273 K, 4: 293 K, 5: 313 K, 6: 333 K, 7: 353 K) in toluene-d_8. The concentration of **1** (15.3 ppm at 293 K) increases by increasing temperature, while the concentration of **5a** (31.4 ppm at 293 K) and **5b** (26.9 ppm at 293 K) decreases. The ratio of **5a/b** seems to be the same at all temperatures.

IR Spectroscopy Data

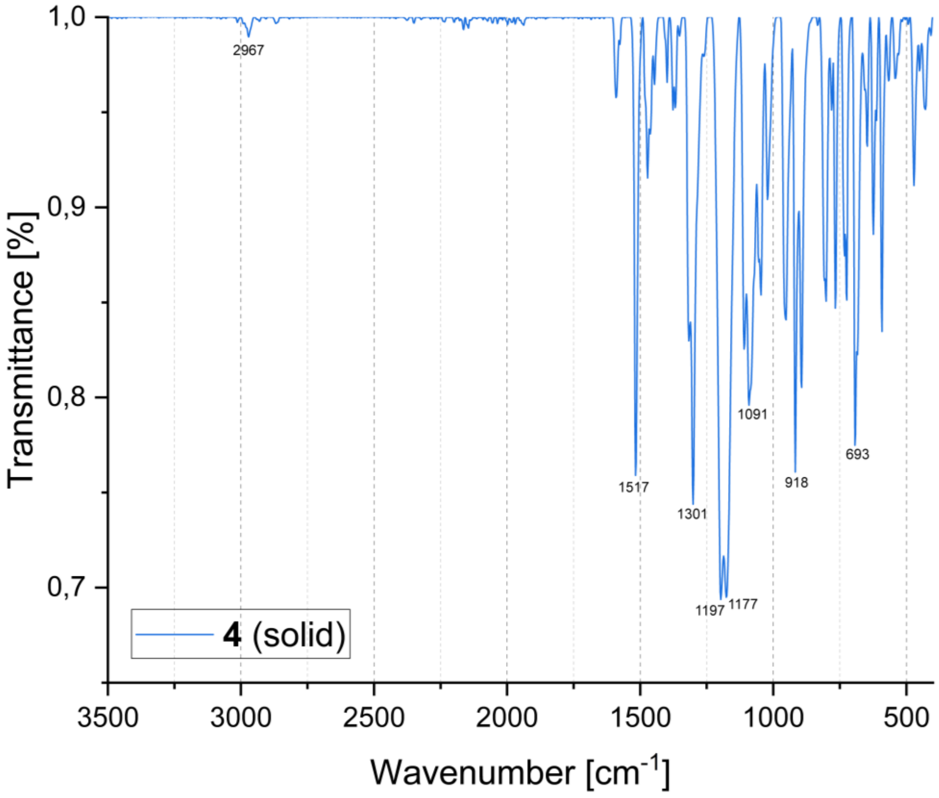

Figure S45 IR spectrum of **4** as solid; some bands were labelled with their respective wavenumber.

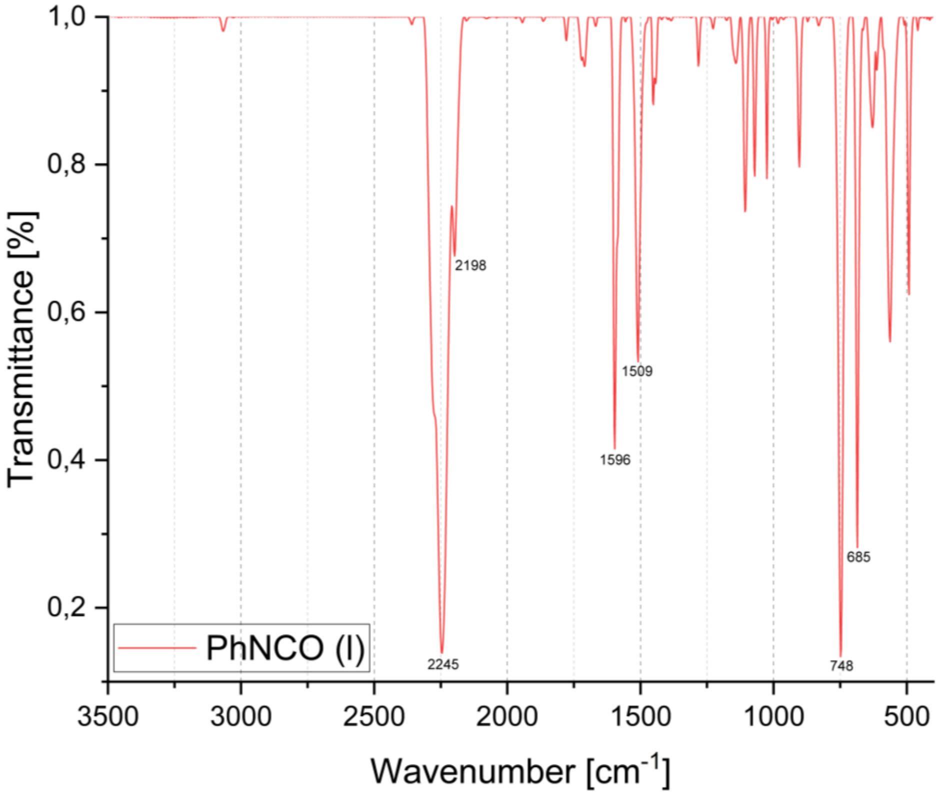

Figure S46 IR spectrum of phenyl isocyanate; some bands were labelled with their respective wavenumber.

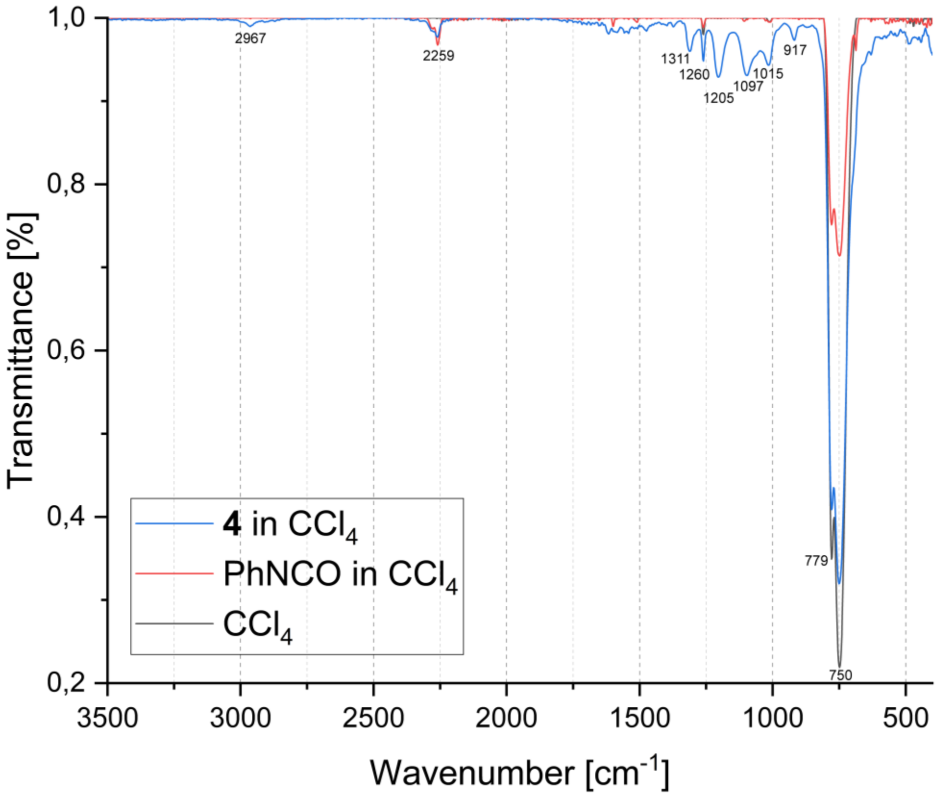

Figure S47 IR spectra of solutions of **4** and PhNCO in CCl_4 and of the solvent CCl_4; some bands were labelled with their respective wavenumber. Note that the solutions have different concentrations.

Crystallographic Data

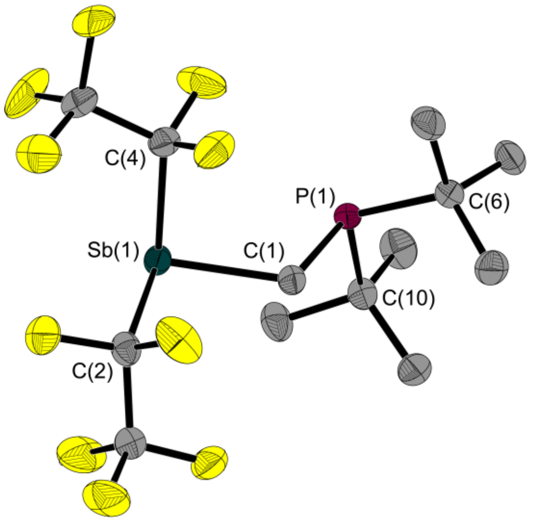

Figure S48 Molecular structure of **1** in the solid state. Ellipsoids are set at 50 % probability; hydrogen atoms and minor occupied disordered atoms are omitted for clarity. Selected bond lengths and interatomic distances [Å] and angles [°]: Sb(1)–C(1) 2.157(1), Sb(1)–C(2) 2.238(1), Sb(1)–C(4) 2.217(2), P(1)–C(1) 1.860(1), P(1)–C(6) 1.891(1), P(1)–C(10) 1.886(1), Sb(1)···P(1) 3.306(1); C(1)–Sb(1)–C(2) 93.2(1), C(1)–Sb(1)–C(4) 91.3(1), C(4)–Sb(1)–C(2) 95.8(1), C(1)–P(1)–C(6) 100.2(1), C(1)–P(1)–C(10) 101.6(1), C(10)–P(1)–C(6) 111.3(1), P(1)–C(1)–Sb(1) 110.6(1).

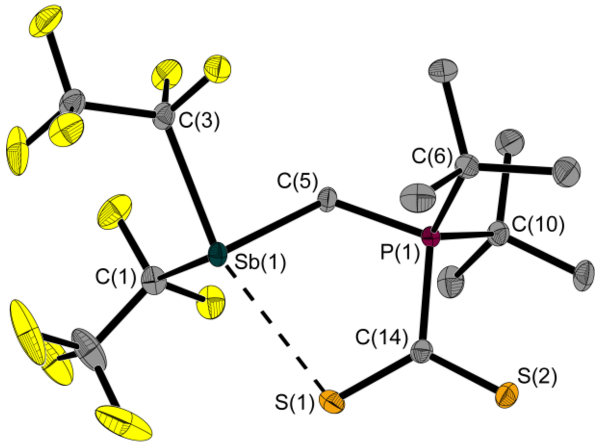

Figure S49 Molecular structure of compound **2** in the solid state. Ellipsoids are set at 50 % probability; hydrogen atoms are omitted for clarity. Selected bond lengths and interatomic distances [Å] and angles [°]: Sb(1)–C(1) 2.265(2), Sb(1)–C(3) 2.293(2), Sb(1)–C(5) 2.193(2), P(1)–C(5) 1.791(2), P(1)–C(6) 1.870(2), P(1)–C(10) 1.875(2), P(1)–C(14) 1.844(2), S(1)–C(14) 1.681(2), S(2)–C(14) 1.657(2), Sb(1)–S(1) 2.964(1); C(1)–Sb(1)–C(3) 91.1(1), C(5)–Sb(1)–C(1) 90.4(1), C(5)–Sb(1)–C(3) 85.9(1), C(5)–P(1)–C(14) 108.4(1), P(1)–C(5)–Sb(1) 115.2(1), S(1)–C(14)–P(1) 116.6(1), S(2)–C(14)–S(1) 127.1(1), S(2)–C(14)–P(1) 116.3(1), S(1)–Sb(1)–C(3) 160.8(1).

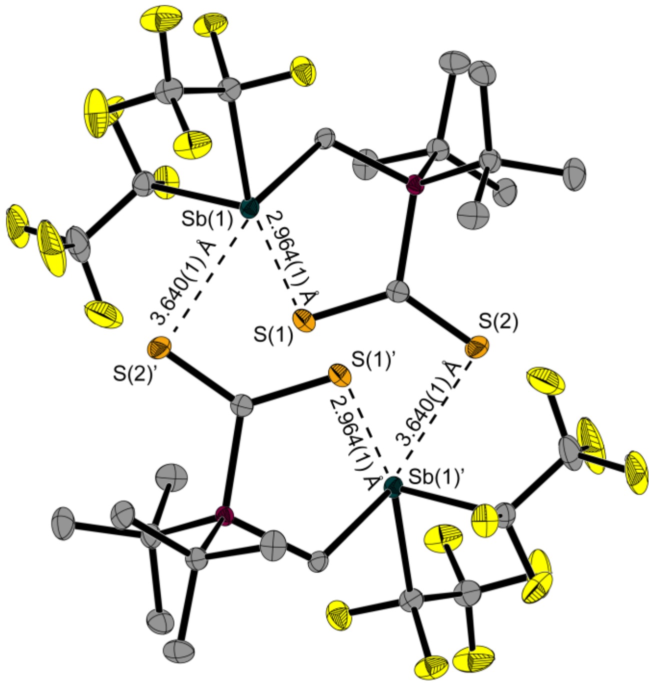

Figure S50 Molecular structure of a dimer of **2**; second molecule was symmetry generated (1−x, 1−y, 1−z). Ellipsoids are set at 50 % probability; hydrogen atoms are omitted for clarity.

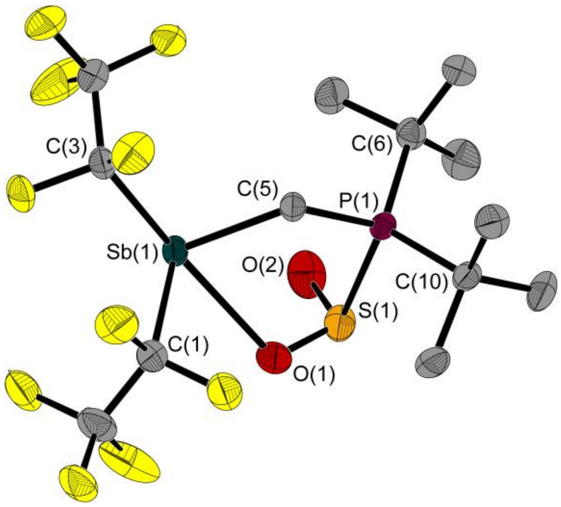

Figure S51 Molecular structure of compound **3** in the solid state. Ellipsoids are set at 50 % probability; hydrogen atoms and minor occupied disordered atoms are omitted for clarity. Selected bond lengths [Å] and angles [°]: Sb(1)–O(1) 2.483(2), Sb(1)–C(1) 2.242(3), Sb(1)–C(3) 2.281(3), Sb(1)–C(5) 2.199(2), S(1)–P(1) 2.285(1), S(1)–O(1) 1.498(2), S(1)–O(2) 1.470(2), P(1)–C(5) 1.803(2), P(1)–C(6) 1.852(3), P(1)–C(10) 1.857(2); C(1)–Sb(1)–O(1) 77.1(1), C(1)–Sb(1)–C(3) 87.7(1), C(3)–Sb(1)–O(1) 158.5(1), C(5)–Sb(1)–O(1) 78.0(1), C(5)–Sb(1)–C(1) 95.5(1), C(5)–Sb(1)–C(3) 88.6(1), O(1)–S(1)–P(1) 95.1(1), O(2)–S(1)–P(1) 102.8(1), O(2)–S(1)–O(1) 111.8(1), C(5)–P(1)–S(1) 104.3(1), S(1)–O(1)–Sb(1) 112.9(1), P(1)–C(5)–Sb(1) 114.9(1).

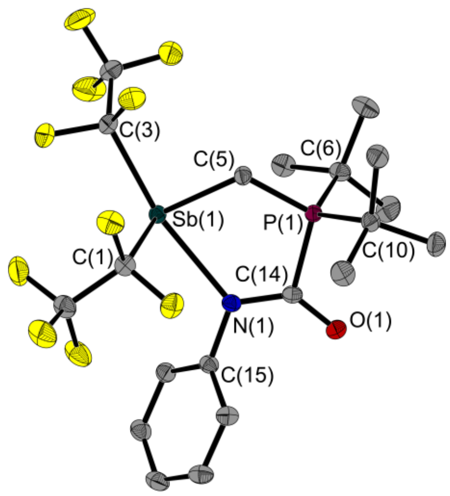

Figure S52 Molecular structure of compound **4** in the solid state. Ellipsoids are set at 50 % probability; hydrogen atoms are omitted for clarity. Selected bond lengths [Å] and angles [°]: Sb(1)–N(1) 2.473(1), Sb(1)–C(1) 2.253(1), Sb(1)–C(3) 2.332(1), Sb(1)–C(5) 2.180(1), P(1)–C(5) 1.792(1), P(1)–C(6) 1.856(1), P(1)–C(10) 1.852(1), P(1)–C(14) 1.860(1), O(1)–C(14) 1.243(2), N(1)–C(14) 1.322(2), N(1)–C(15) 1.428(2); C(1)–Sb(1)–N(1) 84.0(1), C(3)–Sb(1)–N(1) 159.7(1), C(5)–Sb(1)–N(1) 78.1(1), C(5)–P(1)–C(14) 108.0(1), C(14)–N(1)–Sb(1) 121.5(1), C(14)–N(1)–C(15) 117.7(1), C(15)–N(1)–Sb(1) 120.8(1), P(1)–C(5)–Sb(1) 113.6(1), O(1)–C(14)–P(1) 116.7(1), O(1)–C(14)–N(1) 132.0(1), N(1)–C(14)–P(1) 111.3(1).

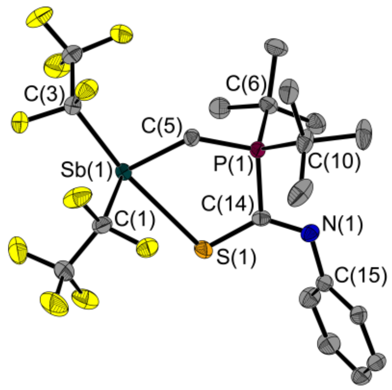

Figure S53 Molecular structure of compound **5a** in the solid state. Only one of the two molecules in the asymmetric unit is shown. Ellipsoids are set at 50 % probability; hydrogen atoms are omitted for clarity. Selected bond lengths [Å] and angles [°]: Sb(1)–S(1) 2.881(1), Sb(1)–C(1) 2.243(2), Sb(1)–C(3) 2.311(2), Sb(1)–C(5) 2.172(2), S(1)–C(14) 1.713(2), P(1)–C(5) 1.792(2), P(1)–C(6) 1.856(2), P(1)–C(10) 1.857(2), P(1)–C(14) 1.837(2), N(1)–C(14) 1.289(2), N(1)–C(15) 1.419(2); C(1)–Sb(1)–S(1) 86.1(1), C(3)–Sb(1)–S(1) 164.7(1), C(5)–Sb(1)–S(1) 80.1(1), C(14)–S(1)–Sb(1) 102.8(1), C(5)–P(1)–C(14) 109.4(1), C(14)–N(1)–C(15) 122.2(2), P(1)–C(5)–Sb(1) 119.3(1), S(1)–C(14)–P(1) 118.0(1), N(1)–C(14)–S(1) 131.8(1); N(1)–C(14)–P(1) 110.2(1).

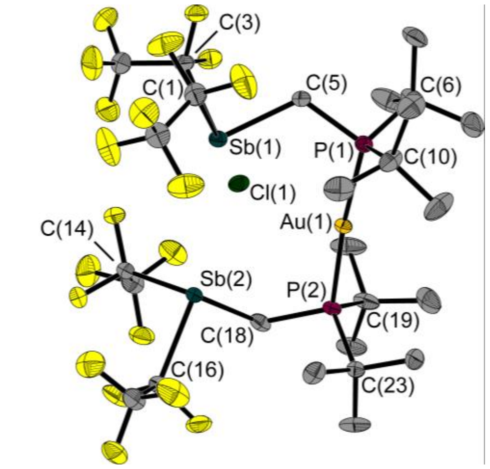

Figure S54 Molecular structure of compound **6** in the solid state. Ellipsoids are set at 50 % probability; hydrogen atoms and minor occupied disordered atoms are omitted for clarity. Selected bond lengths and interatomic distances [Å] and angles [°]: Au(1)–P(1) 2.311(1), Au(1)–P(2) 2.319(1), Sb(1)–C(1) 2.309(3), Sb(1)–C(3) 2.259(3), Sb(1)–C(5) 2.169(2), Sb(2)–C(14) 2.173(5), Sb(2)–C(16) 2.310(3), Sb(2)–C(18) 2.153(3), P(1)–C(5) 1.823(3), P(2)–C(18) 1.826(3), Au(1)···Cl(1) 2.939(1), Cl(1)···Sb(1) 2.966(1), Cl(1)···Sb(2) 2.981(1); P(1)–Au(1)–P(2) 174.6(1), C(3)–Sb(1)–C(1) 89.7(1), C(5)–Sb(1)–C(1) 93.3(1), C(5)–Sb(1)–C(3) 86.9(1), C(14)–Sb(2)–C(16) 89.8(1), C(18)–Sb(2)–C(16) 91.0(1), C(18)–Sb(2)–C(14) 107.1(2), C(5)–P(1)–Au(1) 112.7(1), C(18)–P(2)–Au(1) 113.7(1), P(1)–C(5)–Sb(1) 118.6(1), P(2)–C(18)–Sb(2) 117.8(1), Sb(1)···P(1)···P(2)···Sb(2) 4.8(1).

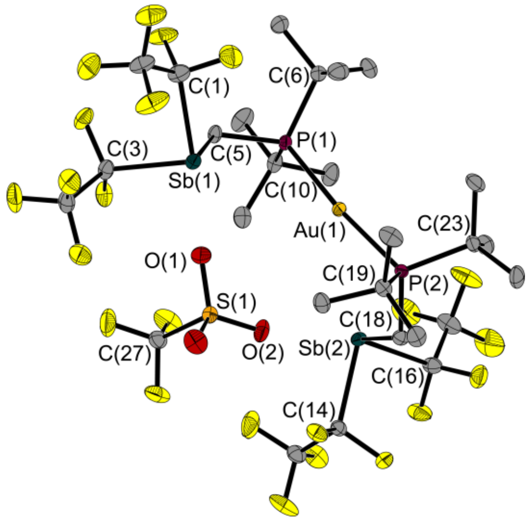

Figure S55 Molecular structure of compound **7** in the solid state. Ellipsoids are set at 50 % probability; hydrogen atoms are omitted for clarity. Selected bond lengths and interatomic distances [Å] and angles [°]: Au(1)–P(1) 2.328(1), Au(1)–P(2) 2.328(1), Sb(1)–C(1) 2.275(2), Sb(1)–C(3) 2.254(2), Sb(1)–C(5) 2.164(2), Sb(2)–C(14) 2.254(2), Sb(2)–C(16) 2.268(2), Sb(2)–C(18) 2.172(2), P(1)–C(5) 1.826(2), P(2)–C(18) 1.831(2), Au(1)···O(2) 3.521(2), O(1)···Sb(1) 2.647(2), O(2)···Sb(2) 2.808(2); P(1)–Au(1)–P(2) 173.9(1), C(3)–Sb(1)–C(1) 92.3(1), C(5)–Sb(1)–C(1) 91.8(1), C(5)–Sb(1)–C(3) 87.1(1), C(14)–Sb(2)–C(16) 89.8(1), C(18)–Sb(2)–C(16) 96.7(1), C(18)–Sb(2)–C(14) 87.6(1), C(5)–P(1)–Au(1) 116.8(1), C(18)–P(2)–Au(1) 111.7(1), P(1)–C(5)–Sb(1) 120.4(2), P(2)–C(18)–Sb(2) 115.8(2), Sb(1)···P(1)···P(2)···Sb(2) 111.1(1).

X-Ray Diffraction Studies

Single crystals were examined on a Rigaku Supernova diffractometer. Using Olex2,[6] the structures were solved with the ShelXT[7] structure solution program using Intrinsic Phasing and refined with the ShelXL[8] refinement package using Least Squares minimisation, **7** was refined with the olex2.refine[9] refinement package using Gauss-Newton minimisation. Refinement using NoSpherA2,[10] an implementation of Non-SPHERical Atom-form-factors in Olex2. The following options were used: SOFTWARE: ORCA 5.0, PARTITIONING: NoSpherA2, INT ACCURACY: Normal, METHOD: R2SCAN, BASIS SET: x2c-TZVP, CHARGE: 0, MULTIPLICITY: 1.

A single crystal of **1** was in situ grown by manually generating a seed at 283.9 K and subsequent cooling to 260 K with 1 K/h and with 50 K/h to 100 K.

Crystals of **2** precipitated from solution during the synthesis.

Solutions of **3** (*n*-hexane) and **4** (CD_2Cl_2) were cooled to −20 °C, whereupon crystals suitable for diffraction experiments formed.

Single crystals of **5a**, **6** and **7** were grown from saturated solutions in *n*-hexane (**5**), CD_2Cl_2 (**6**) and C_6D_6 (**7**).

CCDC 2350371-2350377 contain the supplementary crystallographic data for this paper. These data can be obtained free of charge from The Cambridge Crystallographic Data Centre via http://www.ccdc.cam.ac.uk/conts/retrieving.html

	FLP **1**[a]	FLP·CS_2 **2**[b]	FLP·SO_2 **3**[c]	FLP·PhNCO **4**
Empirical formula	$C_{13}H_{20}F_{10}PSb$	$C_{14}H_{20}F_{10}PS_2Sb$	$C_{13}H_{20}O_2F_{10}PSSb$	$C_{20}H_{25}F_{10}NOPSb$
M_r	519.01	595.14	583.07	638.13
λ [Å]	0.71073	0.71073	0.71073	0.71073
T [K]	100.0(1)	100.0(1)	100.0(1)	100.0(1)
F(000) [e]	2032	584	2288	1264
Crystal system	orthorhombic	triclinic	orthorhombic	monoclinic
Space group	*Pbca*	$P\overline{1}$	*Pbca*	$P2_1/c$
a [Å]	16.0957(2)	8.6271(6)	17.0497(5)	15.3875(2)
b [Å]	11.6873(1)	11.2928(9)	11.9276(3)	11.5169(1)
c [Å]	20.2429(2)	12.0061(10)	20.3495(6)	14.6407(2)
α [°]	90	74.243(7)	90	90
β [°]	90	74.812(7)	90	106.704(1)
γ [°]	90	70.602(7)	90	90
V [Å^3]	3808.00(7)	1042.64(15)	4138.3(2)	2485.09(5)
Z	8	2	8	4
$\rho_{calcd.}$ [g cm^{-3}]	1.811	1.896	1.872	1.706
μ [mm^{-1}]	1.619	1.685	1.605	1.262
2Θ range [°]	5.896–73.81	5.508–65.616	4.004–60.16	4.488–68.784
Index ranges h	$-26 \le h \le 26$	$-9 \le h \le 12$	$-24 \le h \le 24$	$-24 \le h \le 24$
Index ranges k	$-19 \le k \le 19$	$-16 \le k \le 16$	$-16 \le k \le 16$	$-17 \le k \le 18$
Index ranges l	$-33 \le l \le 34$	$-18 \le l \le 17$	$-28 \le l \le 28$	$-23 \le l \le 23$
Reflexes collected	186026	13487	77118	147790
Independent reflexes	9432	6656	6082	10161
R_{int}	0.0462	0.0343	0.0476	0.0667
Observed reflexes, $I > 2\sigma(I)$	8176	6207	4917	9052
Data/restrains/ parameters	9432/0/358	6656/0/333	6082/99/305	10161/0/313
R_1/wR_2 [$I > 2\sigma(I)$]	0.0272/0.0589	0.0292/0.0696	0.0320/0.0699	0.0287/0.0709
R_1/wR_2 (all data)	0.0337/0.0616	0.0315/0.0723	0.0453/0.0751	0.0337/0.0739
GoF on F^2	1.031	1.069	1.076	1.108
ρ_{max}/ρ_{min} [e Å^{-3}]	0.85/−1.21	0.72/−0.72	1.26/−0.60	1.64/−0.91
CCDC number	2350371	2350372	2350373	2350374

[a] **1** shows a disorder of one C_2F_5 group with the ratio 77:23. The pairs F(8)/F(8B) and F(10)/F(10B) are nearer than the resolution of 0.59 Å, therefore thermal parameters were constrained to be the same. [b] Hydrogen atoms were refined isotropically. [c] Disorder of one C_2F_5 group over two sides (55:45).

	FLP·PhNCS **5a**[d]	FLP·AuCl **6**[e]	FLP·AuOTf **7**
Empirical formula	$C_{20}H_{25}F_{10}NPSSb$	$C_{26}H_{40}AuClF_{20}P_2Sb_2$	$C_{27}H_{40}O_3F_{23}P_2SSb_2Au$
M_r	654.19	1270.43	1384.08
λ [Å]	0.71073	0.71073	0.71073
T [K]	100.0(1)	100.0(1)	100.0(1)
F(000) [e]	1296	1208	2633.473
Crystal system	triclinic	triclinic	orthorhombic
Space group	$P\overline{1}$	$P\overline{1}$	$P2_12_12_1$
a [Å]	9.9191(2)	8.8715(2)	11.7280(2)
b [Å]	16.6017(3)	10.8958(2)	18.5033(3)
c [Å]	17.2503(3)	21.7596(4)	19.9201(3)
α [°]	64.175(2)	77.654(2)	90
β [°]	85.392(1)	85.919(1)	90
γ [°]	85.358(1)	80.909(2)	90
V [Å^3]	2545.37(9)	2027.35(7)	4322.80(12)
Z	4	2	4
$\rho_{calcd.}$ [g cm^{-3}]	1.707	2.081	2.127
μ [mm^{-1}]	1.311	5.188	4.877
2Θ range [°]	5.062–65.776	4.658–70.62	6.52–78.16
Index ranges h	$-14 \le h \le 15$	$-14 \le h \le 14$	$-20 \le h \le 20$
Index ranges k	$-25 \le k \le 25$	$-17 \le k \le 17$	$-32 \le k \le 31$
Index ranges l	$-26 \le l \le 25$	$-35 \le l \le 35$	$-35 \le l \le 34$
Reflexes collected	142479	160871	200413
Independent reflexes	17900	17433	24648
R_{int}	0.0545	0.0393	0.0468
Observed reflexes, $I > 2\sigma(I)$	14475	15922	22719
Data/restrains/ parameters	17900/60/656	17433/0/545	24648/0/652
R_1/wR_2 [$I > 2\sigma(I)$]	0.0300/0.0622	0.0303/0.0595	0.0230/0.0444
R_1/wR_2 (all data)	0.0454/0.0680	0.0357/0.0614	0.0298/0.0481
GoF on F^2	1.035	1.086	1.069
ρ_{max}/ρ_{min} [e Å^{-3}]	0.99/−0.51	2.70/−2.26	1.57/−1.24
Flack parameter			−0.0142(8)
CCDC number	2350375	2350376	2350377

[d] Disorder of one CF_3 group over two sides (52:48). [e] Disorder of one C_2F_5 group over two sides (56:44).

Quantum Chemical Calculations

Adduct formation of 1 with small molecules

The starting geometries were taken from crystallographic data if available or otherwise built in Molden (Version 6.9).[11,12] After optimisation with the xtb suite,[13–17] the composite method r^{2}SCAN-3c[18–23] was used for final geometry optimisation and vibrational analysis in Orca 5.0.2.[24–27] Enthalpies and Free Gibbs Energies were obtained at the same level of theory by using rotational entropies computed according to Herzberg[28] and vibrational entropies computed using the quasi-rigid-rotor-harmonic-oscillator (QRRHO) approximation.[29] The data used in the calculation of reaction enthalpies ΔH and Gibbs free energies ΔG is shown in Table S1. Cartesian coordinates and relevant data of all computed structures can be found below.

Table S1 The enthalpies and Gibbs free energies of **1**, several small molecules and the corresponding adducts as well as the reaction enthalpy ΔH and Gibbs free energy ΔG of the adduct formation reaction calculated with the composite method r^{2}SCAN-3c.

	T / K, p / atm	Total enthalpy / E_h	Final Gibbs free energy / E_h	Molecule	T / K, p / atm	Total enthalpy / E_h	Final Gibbs free energy / E_h	ΔH / kJ mol^{-1}	ΔG / kJ mol^{-1}
**1**	298, 1	−2087.074280	−2087.163830						
	298, 10	−2087.074307	−2087.161614						
	233, 1	−2087.085052	−2087.146619						
	233, 10	−2087.085052	−2087.144920						
CO_2	298, 1	−188.554807	−188.579075	**1**·CO_2	298, 1	−2275.640467	−2275.734116	−30	23
	298, 10	−188.554810	−188.576889		298, 10	−2275.640496	−2275.731899	−30	17
	233, 1	−188.555690	−188.573878		233, 1	−2275.652235	−2275.716136	−30	11
	233, 10	−188.555690	−188.572179		233, 10	−2275.652235	−2275.714437	−30	7
CS_2	298, 1	−834.431097	−834.458100	**1**·CS_2	298, 1	−2921.525635	−2921.620495	−53	4
	233, 1	−834.432180	−834.452329		233, 1	−2921.537609	−2921.602309	−54	−9
P(*t*Bu)$_2$Me	298, 1	−696.526794	−696.579198	P(*t*Bu)$_2$Me·CS_2	298, 1	−1530.962801	−1531.023659	−13	36
PhNCO	298, 1	−399.541382	−399.581271	**1**·Ph**NC**O	298, 1	−2486.646789	−2486.749061	−82	−10
				**1**·PhN**CO**		−2486.644075	−2486.747544	−75	−6
PhNCS	298, 1	−722.486005	−722.526997	**1**·Ph**NC**S	298, 1	−2809.592235	−2809.695109	−84	−11
				**1**·PhN**CS**		−2809.592447	−2809.696820	−84	−16

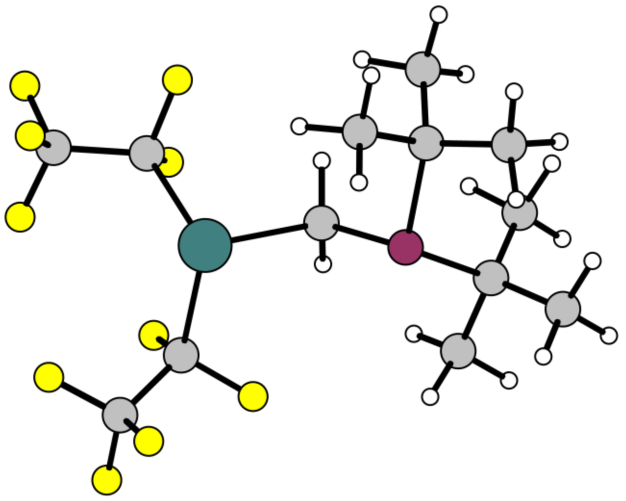

Figure S56 Molecular structure of **1** obtained from geometry optimisation at r^{2}SCAN-3c-D4/def2-TZVPP level of theory.

45

Coordinates from ORCA-job jonas_flp_frei_r2scan-3c_def2-tzvpp_d4

Sb	-0.63450975229723	-0.30444015114203	-0.58813637929149
P	2.61661783614345	0.30703008185624	-0.44761918955897
F	-2.07919154586250	1.59099344296584	1.35374410987942
F	-0.47835089800951	2.57865170218435	0.21171341853303
F	-1.96366597635449	2.44029525077127	-2.05670247530470
F	-3.03941151592227	3.41356989876977	-0.42818093013291
F	-3.55138040316357	1.37372665526154	-1.00627742445890
F	-1.41289210013971	-1.03412540272230	2.29056376740186
F	-0.79694854652710	-2.73127727181833	1.03013489148609
F	-3.16281462157581	-2.56181656011326	-0.35098113289289
F	-3.79417197587214	-0.88452547694996	0.89451955858841
F	-3.42963282732555	-2.83507592456888	1.79619740215948
C	-1.49924577350852	1.66908376032307	0.12676034785423
C	-2.53757441379075	2.24602427912466	-0.85429400427387
C	-1.54135825044603	-1.57253526646351	1.04936581393247
C	-3.01273964047046	-1.97625604788743	0.85352027446644
C	1.14579998268873	-0.04141952116050	0.64831471015005
H	0.93122045077939	0.82628899295537	1.27477409815112
H	1.24871197185710	-0.91165913169714	1.30236362921036
C	3.33302966083003	-1.45763970251736	-0.66205412718402
C	2.46196545139531	-2.11524418534212	-1.74874717918136
H	2.87465520715980	-3.10167244965072	-1.99713626959404
H	1.43332793006703	-2.28138143531198	-1.40643070488756
H	2.42703732101249	-1.51503099600530	-2.66365796470691
C	3.32513958891446	-2.36842399344622	0.57073129252409
H	2.31074711044083	-2.61213398710327	0.90033114694419
H	3.80651292909701	-3.32072870439285	0.31075770694519
H	3.87019352551444	-1.94488904219978	1.41652893600507
C	4.76479377824031	-1.35144113803473	-1.20481352720795
H	5.47406151922792	-1.05512675103120	-0.42686617633345
H	5.08253224741855	-2.33300542989722	-1.57892506053048
H	4.83621628136617	-0.63775171613451	-2.03344498364809
C	3.72458544158776	1.35966747084186	0.70440207285016
C	2.88892371148732	2.60107911172672	1.07044598065580
H	2.10428805837813	2.38273776596993	1.80017621205084
H	3.54904631324650	3.35106208795114	1.52338565511751
H	2.42219467101561	3.05297685386084	0.18786573923714
C	4.20433440952931	0.67901224017969	1.98724176198077
H	4.92566461195644	-0.11662429187137	1.78030552024899
H	4.70580679207325	1.41511396324175	2.63030962550132
H	3.37166432827279	0.25368577385836	2.55854189729136
C	4.92677672805541	1.85488384676036	-0.11713140718579
H	5.61404917638187	1.05172715311896	-0.38887414154543
H	4.59894127192719	2.34966144225845	-1.03771769283032
H	5.49220493520086	2.58492180348182	0.47627220158369

Point Group: C_1, Symmetry Number: 1
Total Enthalpy ... -2087.07428012 E_h
Final Gibbs free energy ... -2087.16382953 E_h

0: 0.00 cm**-1
1: 0.00 cm**-1
2: 0.00 cm**-1
3: 0.00 cm**-1
4: 0.00 cm**-1
5: 0.00 cm**-1
6: 6.21 cm**-1
7: 13.58 cm**-1
8: 19.06 cm**-1
9: 32.44 cm**-1
10: 38.38 cm**-1
11: 45.41 cm**-1
12: 48.67 cm**-1

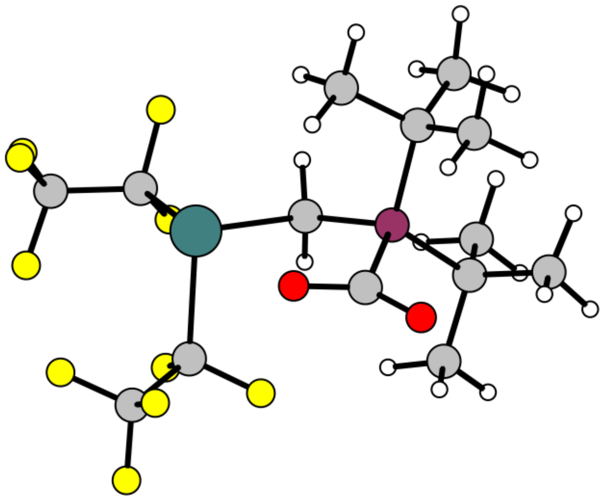

Figure S57 Molecular structure of **1**·$\mathbf{CO_2}$ obtained from geometry optimisation at r^{2}SCAN-3c-D4/def2-TZVPP level of theory.

```
48
Coordinates from ORCA-job jonas_flp_co2_r2scan-3c_def2-tzvpp_d4
  C   0.82545203092845   -0.74813339129905    0.61734026011100
  P   2.44223004427045   -0.13464420791459    0.09885683628641
  C   2.13978913179861    1.39894378232828   -1.06888561405103
  O   3.09486363324929    2.14947161457634   -1.19095095943404
  C   3.25038225073734   -1.44558293976964   -0.98337508096530
  C   4.39389177430220   -0.82145021821840   -1.79922201424185
  C   3.40281219572330    0.39194335161055    1.62473885895956
  C   4.86374114122421    0.69842138852472    1.26839619400553
  Sb -0.80132484809591   -0.22477710770175   -0.79749447660333
  C  -1.99928281616838   -1.68691302724342    0.53382785572620
  C  -3.50820465190747   -1.78482352379012    0.27028676875261
  F  -4.08152016239117   -2.81731528631593    0.91389643573097
  C  -1.35775009565160    1.58540290002985    0.45718939039808
  C  -2.25336731696673    2.57657373082913   -0.31049165164787
  F  -3.37217424542120    1.93782595927856   -0.71234734847986
  F  -2.02387249673847    1.22529842192701    1.59161967580234
  F  -0.23611265808049    2.25785103510417    0.85345458919610
  F  -1.61527811038601    3.03557372340352   -1.39543781705174
  F  -2.61254465362600    3.62134663960182    0.45155802272038
  F  -1.81962640482141   -1.57464788512361    1.88486025962389
  F  -1.46172026378835   -2.91704894590377    0.17076970774460
  F  -3.72049504913845   -1.94708826921656   -1.05358307375011
  F  -4.12173825451890   -0.65464355251761    0.65881938900546
  C   2.17715989572646   -1.94937697683287   -1.96528526175669
  C   3.77060195143728   -2.62247532768651   -0.14853720080697
  C   2.72748214975753    1.67974172128837    2.13168257741100
  C   3.33209950087403   -0.68210527889746    2.72029730048200
  O   0.99437653869111    1.36597005246712   -1.57875854483421
  H   0.54742030376722   -0.29358317433041    1.57255061668672
  H   0.81408213823029   -1.83455123400918    0.74376052560939
  H   2.66027455115819   -2.63726619407744   -2.66898565973171
  H   1.37586696621785   -2.50386246237130   -1.46662646505218
  H   1.73916694151959   -1.13268621946369   -2.54853183168740
  H   2.99391768409813   -3.05790323270045    0.48927939258762
  H   4.11439399002825   -3.40847008995981   -0.83177207168737
  H   4.62063672241856   -2.33727807932745    0.47688296586527
  H   5.19475922873939   -0.42343223406045   -1.17366581377674
  H   4.82221773484487   -1.60550779383631   -2.43488355016473
  H   4.03868496966632   -0.01345895485170   -2.44392625198565
  H   1.68386518294045    1.52142923881539    2.41820486008949
  H   3.26657493332524    2.01246231050962    3.02639214507779
  H   2.77148490283394    2.47910452406976    1.38741500292296
  H   3.81739000766186   -1.61607909322898    2.42782473966354
```

H	3.85428863912098	-0.30102410954873	3.60611656265252
H	2.30270962396279	-0.90471038123577	3.01667653233136
H	5.42219984016613	-0.20636997740031	1.01173893099668
H	4.93306881390707	1.41553668572774	0.44571783078753
H	5.34423561437312	1.13879208874132	2.15017346048179

Point Group: C_1, Symmetry Number: 1
Total Enthalpy ... -2275.64046693 E_h
Final Gibbs free energy ... -2275.73411595 E_h
0: 0.00 cm**-1
1: 0.00 cm**-1
2: 0.00 cm**-1
3: 0.00 cm**-1
4: 0.00 cm**-1
5: 0.00 cm**-1
6: 15.68 cm**-1
7: 18.03 cm**-1
8: 36.54 cm**-1
9: 41.94 cm**-1
10: 46.41 cm**-1
11: 48.58 cm**-1
12: 60.73 cm**-1

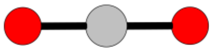

Figure S58 Molecular structure of **CO_2** obtained from geometry optimisation at r^{2}SCAN-3c-D4/def2-TZVPP level of theory.

3
Coordinates from ORCA-job jonas_co2_r2scan-3c_def2-tzvpp_d4

C	-0.00000000503786	0.00000000476514	-0.00000000000972
O	0.00000000251893	-0.00000000238257	1.16342532989592
O	0.00000000251893	-0.00000000238257	-1.16342532988620

Point Group: $D_{\infty h}$, Symmetry Number: 2
Total Enthalpy ... -188.55480747 E_h
Final Gibbs free energy ... -188.57907466 E_h
0: 0.00 cm**-1
1: 0.00 cm**-1
2: 0.00 cm**-1
3: 0.00 cm**-1
4: 0.00 cm**-1
5: 666.21 cm**-1
6: 666.21 cm**-1
7: 1366.45 cm**-1
8: 2431.59 cm**-1

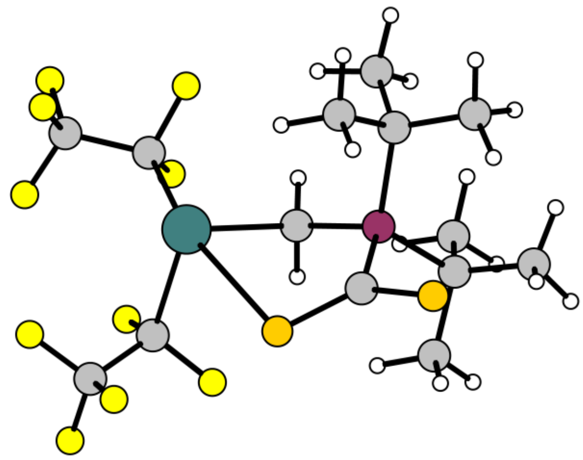

Figure S59 Molecular structure of **2** obtained from geometry optimisation at r²SCAN-3c-D4/def2-TZVPP level of theory.

48

Coordinates from ORCA-job jonas_flp_cs2_r2scan-3c_def2-tzvpp_d4

Sb	0.92391209238229	0.03347801525926	-0.67136665070325
S	-1.02022424868780	-2.06390989381207	-1.20733047917442
S	-3.98294513321531	-1.59281321667229	-1.53366056112591
P	-2.39300576933723	0.23314587488347	0.19662271134025
F	2.64793595388416	-0.91317036009009	1.67842363011768
F	0.90956635563863	-2.21689263545705	1.39552234919634
F	2.02674145843574	-3.16593475377269	-0.94943486660703
F	3.32056442954938	-3.40124684694665	0.78845574013138
F	3.73187435390820	-1.83370247579163	-0.67258765273291
F	1.98003332312375	1.79305757908134	1.77232878253911
F	1.26347885103463	2.90129320175697	0.00629672696262
F	3.49192165022857	2.17788014665606	-1.38385907509623
F	4.25661268399097	1.06619743024708	0.33052986346555
F	3.94276620021643	3.21708968271033	0.48055526540562
C	1.86709898689348	-1.50247087090844	0.73181181006067
C	2.75449602344912	-2.50441377250527	-0.03202470149969
C	2.00928143489598	1.79571595637252	0.40528173757828
C	3.45123402328006	2.07683855418313	-0.03773722595764
C	-0.69167065449335	0.46612553427654	0.77164635824203
H	-0.50807131831577	-0.24893811260086	1.57828473198771
H	-0.55675442806399	1.47281247658260	1.17159563127503
C	-2.87727219263181	1.78957745454130	-0.76805757642231
C	-2.25780633237500	1.64563680194643	-2.16964006193296
H	-2.43701687045557	2.57917803144613	-2.71545609831916
H	-1.17271646869056	1.49486921552400	-2.13413683123226
H	-2.70808275830429	0.82359183878934	-2.73121066715733
C	-2.29333446618492	3.03879736199095	-0.08843333598616
H	-1.20236466836893	3.06820273038705	-0.12807043262767
H	-2.66267388754434	3.91449069582311	-0.63476296557524
H	-2.61493047172014	3.14555301693730	0.95187705602862
C	-4.39731921809812	1.95531711929294	-0.90188777354686
H	-4.85162463240018	2.28573352162452	0.03532614651602
H	-4.58128458361923	2.73675087881186	-1.64867847414526
H	-4.88693241802798	1.03837657420337	-1.23825065853690
C	-3.38036165251107	-0.18719353916536	1.76089478756997
C	-2.96339836650880	-1.61719695690469	2.15286117064693
H	-1.88151079456112	-1.74043528732196	2.26201190270250
H	-3.42076227637091	-1.84456567212299	3.12254935805845
H	-3.32057856802916	-2.35019946014240	1.42502539687562
C	-2.99209894210157	0.79623539681140	2.87716709909510
H	-3.25987453111037	1.82753281216269	2.62876704106151
H	-3.55458352710338	0.52156707889291	3.77713997189391
H	-1.92912752779115	0.75960600496013	3.12882522012762

C	-4.90223294549104	-0.15017464175375	1.57192862563044
H	-5.27932154177034	0.86982895009613	1.48338858992560
H	-5.22296333678865	-0.72601434387228	0.70131498999486
H	-5.35598654197080	-0.59257046290966	2.46680743142885
C	-2.46651474826847	-1.23330563350074	-0.93209203747910

Point Group: C_1, Symmetry Number: 1
Total Enthalpy ... -2921.52563463 E_h
Final Gibbs free energy ... -2921.62049473 E_h

0: 0.00 cm**-1
1: 0.00 cm**-1
2: 0.00 cm**-1
3: 0.00 cm**-1
4: 0.00 cm**-1
5: 0.00 cm**-1
6: 22.50 cm**-1
7: 23.22 cm**-1
8: 34.03 cm**-1
9: 39.39 cm**-1
10: 41.74 cm**-1
11: 50.47 cm**-1
12: 55.71 cm**-1

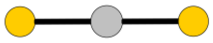

Figure S60 Molecular structure of **CS_2** obtained from geometry optimisation at r^{2}SCAN-3c-D4/def2-TZVPP level of theory.

3
Coordinates from ORCA-job jonas_cs2_r2scan-3c_def2-tzvpp_d4

C	0.00000000297790	-0.00000000281344	-0.00000000011395
S	-0.00000000148895	0.00000000140672	1.56062319819370
S	-0.00000000148895	0.00000000140672	-1.56062319807975

Point Group: D_{infh}, Symmetry Number: 2
Total Enthalpy ... -834.43109677 E_h
Final Gibbs free energy ... -834.45809978 E_h

0: 0.00 cm**-1
1: 0.00 cm**-1
2: 0.00 cm**-1
3: 0.00 cm**-1
4: 0.00 cm**-1
5: 397.24 cm**-1
6: 397.24 cm**-1
7: 675.79 cm**-1
8: 1576.24 cm**-1

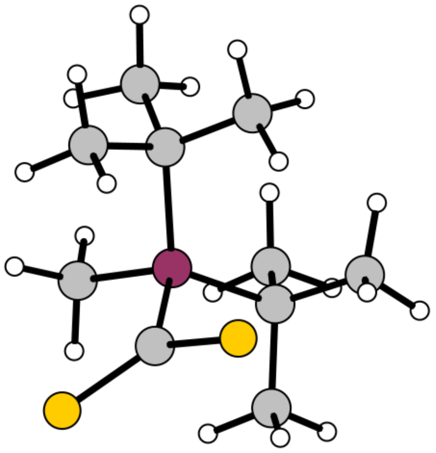

Figure S61 Molecular structure of **P(tBu)$_2$Me·CS$_2$** obtained from geometry optimisation at r^{2}SCAN-3c-D4/def2-TZVPP level of theory.

34
Coordinates from ORCA-job jonas_ptbu2me_cs2_r2scan-3c_def2-tzvpp_d4

P	0.92528607322546	0.02276930217023	-0.68290660094742
C	-0.35698468323668	-1.23294997974744	-1.02248661843006
C	-0.01088474305336	1.66687856258884	-0.63226462449340
C	-1.08112400908538	1.70470527858236	-1.73535144990469
C	2.22321819301164	-0.22847708014961	-2.03755525295280
C	1.54002742582082	-0.48198077650249	-3.39073249016411
C	3.19412788179353	0.95083235142378	-2.17856543830263
C	3.01550478136974	-1.48370806110241	-1.62530730328805
C	0.90278861783962	2.89074467295723	-0.77854306966376
H	0.10142574131844	-2.22366647184310	-0.99183437663815
H	-0.83507508841851	-1.06407295907660	-1.99009673162484
H	0.32064101294401	3.77883357149488	-0.50440510469572
H	-0.65488446881483	1.59629887335103	-2.73735270809643
H	-1.57122801097497	2.68469036160028	-1.69385832060740
H	-1.85553792836749	0.94562464982145	-1.59649390254232
H	1.23969879057922	3.02461703835672	-1.80931333271589
H	1.77202613741927	2.82798518600573	-0.11944924477552
H	0.91682902298383	0.35890574314254	-3.71009762531326
H	2.32572143693289	-0.60859118767560	-4.14522660282318
H	0.93550944127617	-1.39280723343517	-3.39328504577930
H	4.02128369441263	0.62717935946226	-2.82183793934875
H	2.72307526324473	1.81228711598191	-2.65711296309945
H	3.60333554592891	1.25591290677268	-1.21243430885646
H	3.71780033798037	-1.72056230359982	-2.43332367013806
H	3.58642056818398	-1.31226556570404	-0.70929602017689
H	2.37405717152124	-2.35759704643447	-1.47417694372158
H	0.03051913017867	1.79365375462174	1.55299131413837
H	-1.32697709347467	0.83579496077845	0.93261739061152
H	-1.34724335104503	2.59784865337147	0.76914499523900
C	-0.70007601344982	1.71312004086038	0.74437936278974
C	1.68110444857962	-0.31900869368086	0.97544483434223
S	1.09691681017636	-1.64779989152423	1.80341960269710
S	2.86937247990720	0.80485787977324	1.38255617010957
H	-1.10087061670762	-1.19960401264136	-0.22337198082740

Point Group: C_1, Symmetry Number: 1
Total Enthalpy ... -1530.96280080 E_h
Final Gibbs free energy ... -1531.02365908 E_h
0: 0.00 cm**-1
1: 0.00 cm**-1
2: 0.00 cm**-1
3: 0.00 cm**-1

4: 0.00 cm**-1
5: 0.00 cm**-1
6: 30.36 cm**-1
7: 51.03 cm**-1
8: 83.73 cm**-1
9: 137.62 cm**-1
10: 138.53 cm**-1
11: 171.92 cm**-1
12: 206.68 cm**-1

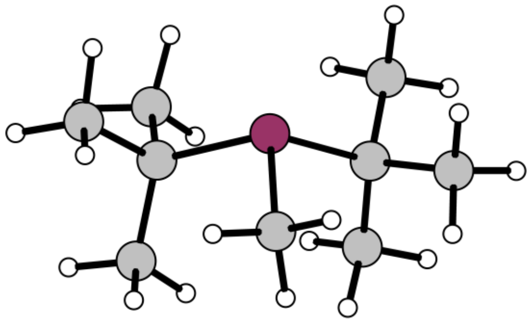

Figure S62 Molecular structure of **P(*t*Bu)$_2$Me** obtained from geometry optimisation at r^{2}SCAN-3c-D4/def2-TZVPP level of theory.

31
Coordinates from ORCA-job jonas_ptbu2me_r2scan-3c_def2-tzvpp_d4

P	1.10712685596457	0.10791165702935	-0.47066032432185
C	-0.10754470357730	-1.29307000278056	-0.66632549578192
C	-0.05628040392452	1.60303769505350	-0.73130973307175
C	-1.16153802146491	1.42666163578463	-1.77827387939226
C	2.20984826993433	-0.14756809853708	-2.01006581744489
C	1.55156719345389	0.05435888272996	-3.37697754132095
C	3.42858900271926	0.78042251401293	-1.87942360870748
C	2.71436204475705	-1.59913003518785	-1.91469343255374
C	0.77075619060072	2.85316065213391	-1.05639951522070
H	0.36919272980755	-2.23051247393073	-0.36799360654849
H	-0.50043455280128	-1.40419258343223	-1.68329253341674
H	0.13680368892325	3.74509216166872	-0.96623343648391
H	-0.76465028427859	1.26121618834952	-2.78205930429890
H	-1.77906013348733	2.33486072563954	-1.81181934690572
H	-1.82549509993722	0.59262866637836	-1.53208958959099
H	1.16230423658242	2.83345364501623	-2.07767491135299
H	1.61296638772353	2.97010720541458	-0.36467436138970
H	1.28491169970989	1.10020599279675	-3.55376091631295
H	2.25382901029681	-0.24098144525113	-4.16898216489451
H	0.65127727607731	-0.55774269585639	-3.49424061225932
H	4.14388603429664	0.56229468598885	-2.68346983894516
H	3.15795863579733	1.83655385949082	-1.95544320759365
H	3.93646002313198	0.63015744609893	-0.92079178951260
H	3.52747795963425	-1.74732986679032	-2.63688037409655
H	3.10760330065649	-1.82653103873504	-0.91649371700225
H	1.92763452197363	-2.32269269798427	-2.14926818409440
H	0.03250628428405	2.01946008137622	1.41802680535797
H	-1.29982764141757	0.94535864473663	0.96206235588304
H	-1.40191133915106	2.67158947914234	0.59400584791047
C	-0.71536756820771	1.81608135208811	0.64495566136441
H	-0.95022759807749	-1.13080223244430	0.01265657199851

Point Group: C_1, Symmetry Number: 1
Total Enthalpy ... -696.52679411 E_h
Final Gibbs free energy ... -696.57919757 E_h
0: 0.00 cm**-1
1: 0.00 cm**-1
2: 0.00 cm**-1
3: 0.00 cm**-1
4: 0.00 cm**-1
5: 0.00 cm**-1
6: 88.76 cm**-1
7: 91.64 cm**-1
8: 170.00 cm**-1
9: 210.96 cm**-1
10: 217.49 cm**-1
11: 225.23 cm**-1
12: 235.59 cm**-1

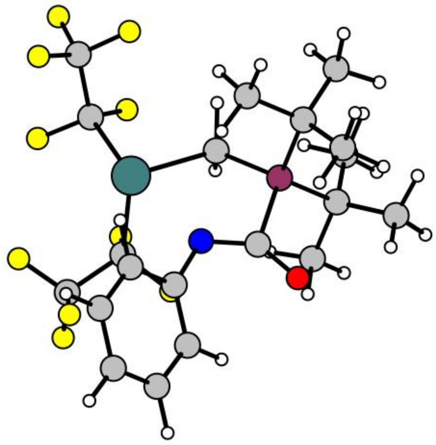

Figure S63 Molecular structure of the energetically favoured regioisomer of **4** (with N···Sb and C···P) obtained from geometry optimisation at r[2]SCAN-3c-D4/def2-TZVPP level of theory.

59
Coordinates from ORCA-job jonas_flp_phnco_nc_r2scan-3c_def2-tzvpp_d4

P	1.11719134491536	0.81806227368918	-0.19542269124041
C	-0.56760802488050	1.41001459428031	-0.42854686139997
Sb	-2.10509551680121	0.09662000344456	0.47054346436297
H	-0.80485675325239	1.43921088506361	-1.49552207367349
H	-0.70854269966436	2.41940840293874	-0.03259207731336
C	-3.58653422428964	1.78479833863400	-0.24315328851042
C	-3.87664921600509	2.74318407961115	0.92234698221932
F	-4.79553184818601	1.28054083612739	-0.64508016745813
F	-3.11380212807475	2.57269771125933	-1.27193503312302
F	-2.69948162730832	3.24285971950057	1.39398029479530
F	-4.47006577490425	2.08281765121019	1.93373977798500
F	-4.65294239430431	3.78528082964257	0.58763101286271
C	-2.44231070622068	-0.92934948134235	-1.53423496270473
C	-3.47131550335206	-2.07511489831163	-1.47015399620871
F	-2.87748823387313	-0.03405463072751	-2.46979786046212
F	-1.27518128201727	-1.45644114203493	-2.01193672676323
F	-4.64663588618114	-1.60516891447560	-1.01457118526609
F	-3.67377963062747	-2.62952013178548	-2.67682690014777
F	-3.04141212917340	-3.02900086327813	-0.62914276443422
C	1.80546964356656	1.57067222270910	1.38424130660881
C	0.70038396520460	1.47854692802235	2.45154868164410
H	4.08119409877710	0.96927641919583	-2.54033732440739
C	3.01716598269218	0.75709909220706	1.86591288530917
C	2.19359669621197	3.04081067861828	1.18013361787546
H	1.12154467343521	1.82410599675104	3.40283558122640

H	-0.15906854254765	2.11678086928412	2.22435619169161
H	0.35567380260920	0.44915251926498	2.59176834036997
H	2.45926705581871	3.46646734367045	2.15510781637372
H	3.06354500267366	3.14928739040627	0.52707950646323
H	1.36985241169711	3.63804183278454	0.77463975591210
H	3.37980184095419	1.20832376141652	2.79716851136425
H	3.83969704987879	0.75940016022937	1.14858321209703
H	2.75180204128936	-0.28345847488616	2.06898880516758
C	2.08906039635952	1.14948338956838	-1.76704056233934
C	1.85762753697271	2.58978569898632	-2.24995753314274
C	1.54296028443563	0.16831971204492	-2.82120046021275
C	3.58811804549716	0.89041809809879	-1.56392865192888
H	2.23782221354758	3.33651454594412	-1.54926780441690
H	4.04775154571179	1.63430825715701	-0.90656281230033
H	3.76889923591373	-0.11145021866581	-1.16624808943480
H	2.39486525542356	2.72062712361763	-3.19672910966183
H	0.80097100529223	2.79951921995399	-2.44107697357646
H	2.06340977540764	0.36536027886752	-3.76570327356678
H	0.47212896669118	0.29928597474243	-3.00431961879647
H	1.72683864317678	-0.87051965303822	-2.53648625717336
C	1.09212114455880	-1.05245877860904	0.09310187376664
N	-0.07412243470682	-1.40113098025810	0.60834707637938
O	2.14042834000357	-1.66296396301727	-0.13816319774789
C	-0.32825480747506	-2.74558221510668	0.96227612346787
C	0.08921104068174	-3.82752221766506	0.17739219850827
C	-0.26204454745958	-5.12147020601396	0.53943956636665
C	-1.02414742867677	-5.36060927507923	1.68057971492335
C	-1.43482535366332	-4.28756032051233	2.46527699704489
C	-1.09072203327844	-2.98872366052539	2.11084641791025
H	0.68039258369707	-3.64618780288977	-0.71248586433154
H	0.06231192458752	-5.95326461697763	-0.07974651972887
H	-1.29542833696699	-6.37543450679133	1.95532342798896
H	-2.02597784956189	-4.46021071929030	3.36018214056930
H	-1.40727873422972	-2.14988506766073	2.72479926021770

Point Group: C_1, Symmetry Number: 1
Total Enthalpy ... -2486.64678858 E_h
Final Gibbs free energy ... -2486.74906063 E_h
0: 0.00 cm**-1
1: 0.00 cm**-1
2: 0.00 cm**-1
3: 0.00 cm**-1
4: 0.00 cm**-1
5: 0.00 cm**-1
6: 21.32 cm**-1
7: 30.11 cm**-1
8: 32.95 cm**-1
9: 36.22 cm**-1
10: 41.24 cm**-1
11: 47.24 cm**-1
12: 49.67 cm**-1

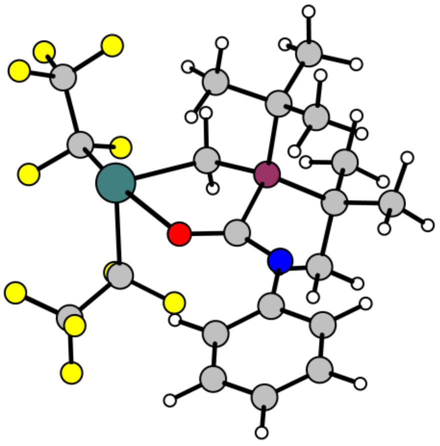

Figure S64 Molecular structure of the energetically favoured regioisomer of **4** (with O···Sb and C···P) obtained from geometry optimisation at r²SCAN-3c-D4/def2-TZVPP level of theory.

59
Coordinates from ORCA-job jonas_flp_phnco_oc_r2scan-3c_def2-tzvpp_d4

Sb	-2.46023653557181	1.08136127620692	-0.87056427455309
C	-1.76891237474293	-0.59895893315338	0.40058285632266
P	-0.01918653638778	-0.99128219264557	0.20271658205603
C	-2.14461808710259	2.55052622948772	0.83468521816948
C	-2.21050291565876	4.01350236166104	0.35850012150666
C	-4.56856518085492	0.61844196155234	0.00832526318521
C	-5.34344745415569	-0.32662849382992	-0.92411391985528
F	-0.92397717413487	2.35779599903641	1.41758400077938
F	-3.07716380316915	2.39534654842255	1.81838833602011
F	-2.04338714275148	4.88282675081406	1.36661729024426
F	-1.25851400809152	4.24063900157559	-0.56286847295537
F	-3.41141221110761	4.24207232864934	-0.20793565306018
F	-4.56225769168849	0.01457071733240	1.24548508041750
F	-5.31502899886856	1.76317845973479	0.11314476797658
F	-4.62820344096092	-1.47273280855811	-1.09624226472379
F	-5.49973370010695	0.23917671999020	-2.13509458577421
F	-6.55370617970977	-0.66888653765213	-0.45904720890708
H	-2.40708772764631	-1.45972603839168	0.18576486615603
H	-1.96770190305022	-0.28827446710299	1.43004641660665
C	0.66253453645903	-1.49338662364933	1.88384039009762
C	-0.29359621011756	-2.48156428581288	2.57218523212689
C	0.74546331975652	-0.20598001968859	2.72242704373139
C	2.06006275136585	-2.11330783418689	1.76136895489787
H	-0.38748224956552	-3.42653065715263	2.03261153571868
H	-1.29348484488198	-2.06278140720087	2.71719816347683
H	0.11449333034581	-2.70767774606223	3.56443227405161
H	1.44275840129272	0.51241554935492	2.28517463217849
H	-0.22822897464439	0.27809613475317	2.84419264845883
H	1.10442829027739	-0.47851582322072	3.72184603193338
H	2.03255826887660	-3.09365067295072	1.27664567836005
H	2.45535287437088	-2.26011693226305	2.77362217006532
H	2.73710539435438	-1.45240148396409	1.21445144385865
C	0.13129692995695	-2.27004846127021	-1.17196356924953
C	1.58478943653427	-2.36322710601538	-1.66053512425807
H	2.27274019548202	-2.69498204522183	-0.88094529054873
H	1.61923493444324	-3.09588800337901	-2.47566768554887
H	1.94465002887855	-1.40428932296541	-2.04042308619338
C	-0.36427826221298	-3.64661420428407	-0.70992320290814
H	-0.39222287444032	-4.31295941667139	-1.58035113828648
H	-1.37617779352051	-3.61091042221306	-0.29256410845205
H	0.30541520570115	-4.09813767515775	0.02685846076476
C	-0.74323670183041	-1.77886214935610	-2.33996059274013

H -1.81046347902779 -1.78813313537490 -2.09740081073709
H -0.46027463625197 -0.77475189076494 -2.67180137832665
H -0.59571949611334 -2.46358804813171 -3.18337070675018
C 0.77123017093766 0.57953089695628 -0.45074458384147
O -0.03051238943896 1.36069218822624 -1.05891236868178
N 2.04766011297954 0.58004354510934 -0.23481502695505
C 2.89933495162455 1.61544788502657 -0.64837166557693
C 4.27376398668634 1.36053733937102 -0.50974506198143
C 5.21692836589091 2.31261902508183 -0.86711138934800
C 4.80975421123920 3.54949987272441 -1.36216657948757
C 3.44977273026773 3.81663416476203 -1.49411940640602
C 2.49514533240405 2.86669145215988 -1.14904574451100
H 4.57894471947479 0.39559457729608 -0.11433726761407
H 6.27480045889338 2.09137980634642 -0.75473194079186
H 5.54528018712480 4.29878662662874 -1.63949342283198
H 3.12259735758151 4.78061204047508 -1.87458739632443
H 1.44122429460623 3.08677517955615 -1.25974103098100

Point Group: C_1, Symmetry Number: 1
Total Enthalpy ... -2486.64407499 E_h
Final Gibbs free energy ... -2486.74754418 E_h
0: 0.00 cm**-1
1: 0.00 cm**-1
2: 0.00 cm**-1
3: 0.00 cm**-1
4: 0.00 cm**-1
5: 0.00 cm**-1
6: 8.23 cm**-1
7: 14.78 cm**-1
8: 16.28 cm**-1
9: 28.98 cm**-1
10: 39.31 cm**-1
11: 42.21 cm**-1
12: 46.08 cm**-1

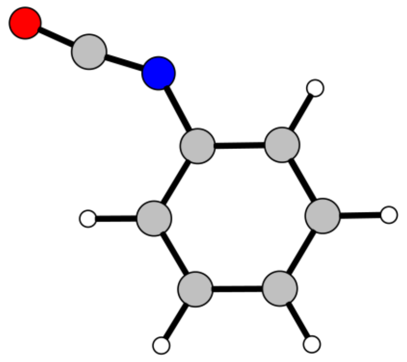

Figure S65 Molecular structure of PhNCO obtained from geometry optimisation at r^2SCAN-3c-D4/def2-TZVPP level of theory.

14
Coordinates from ORCA-job jonas_phnco_r2scan-3c_def2-tzvpp_d4
C 0.01290609215540 0.00073550114333 -0.29676041283612
C -0.22032760268064 -0.00050080555484 1.07881015592310
C 0.85418171507347 -0.00144548033292 1.97392083327375
C 2.15360136373669 -0.00134339492459 1.48492577484784
C 2.39273925533888 -0.00027482753411 0.11268303070775
C 1.31861643483960 0.00086981798576 -0.77225894447680
N -1.54705969123008 -0.00074422234079 1.52254542875056
C -2.10697429236663 0.00142411484081 2.59335907194295
H 0.66376288856335 -0.00215604506315 3.04377592481543
H 2.98556031773665 -0.00217637904267 2.18307623870024
H 3.41096711519360 -0.00029782249935 -0.26330337997126

H 1.49553476022976 0.00188211796856 -1.84369856516558
H -0.83256805258800 0.00167515607251 -0.97682849629889
O -2.77382730400205 0.00235226928145 3.55645533978703

Point Group: C_1, Symmetry Number: 1
Total Enthalpy ... -399.54138170 E_h
Final Gibbs free energy ... -399.58127097 E_h
0: 0.00 cm**-1
1: 0.00 cm**-1
2: 0.00 cm**-1
3: 0.00 cm**-1
4: 0.00 cm**-1
5: 0.00 cm**-1
6: 51.93 cm**-1
7: 94.66 cm**-1
8: 234.49 cm**-1
9: 392.36 cm**-1
10: 412.81 cm**-1
11: 473.01 cm**-1
12: 495.87 cm**-1

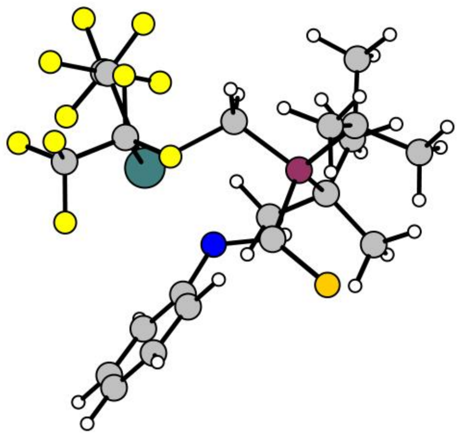

Figure S66 Molecular structure of the regioisomer of **5** with N···Sb and C···P contacts obtained from geometry optimisation at r[2]SCAN-3c-D4/def2-TZVPP level of theory.

59
Coordinates from ORCA-job jonas_flp_phncs_nc_r2scan-3c_def2-tzvpp_d4
P 1.07241083784699 0.89970836885949 0.16664544486268
C -0.68482997612487 1.19263499597578 0.46935706631772
Sb -2.06472153768870 -0.22670335404676 -0.50297080298118
H -0.89206899009737 1.09017797601380 1.53816602288419
H -0.97614248734086 2.20442565361332 0.17794293446985
C -2.36938973853226 -1.33141497532673 1.46558967334324
C -3.28470953095102 -2.56594358299826 1.34402829138749
F -2.91376930691897 -0.50364486593217 2.40539490667216
F -1.17283023534869 -1.75958110372886 1.96868243822177
F -4.48910380965239 -2.19360660714913 0.87569644198048
F -2.75133486853884 -3.45001118784052 0.48547286015716
F -3.45830580553615 -3.17331645717068 2.52932689512164
C -3.73127410466691 1.28211476754870 0.18237964365165
C -4.06992710879698 2.24858071259644 -0.96298147326007
F -3.38659637673248 2.07175424093998 1.25781497482936
F -4.89641272626268 0.63921807534792 0.50741457731711
F -5.00209377742779 3.15911836959142 -0.64524711610499
F -4.49913447568627 1.56253489917411 -2.03887736061832
F -2.94391016502480 2.92254055309096 -1.32952016086962
C 1.97161660022429 1.35577378734387 1.76271185884938
C 1.71728948298878 0.20493479866676 2.75248533271398

C	1.37448344492675	2.65269473185826	2.33860643517117
C	3.48078859210592	1.54470152449359	1.56043622119882
H	2.15055654512567	0.48939314955586	3.71851638171521
H	0.65063057877549	0.01612635899255	2.91332253308226
H	2.19184241629234	-0.72062937676641	2.41793117559988
H	1.91437901619237	2.87874210751108	3.26540114899961
H	0.79229499659175	2.04946623251343	-3.37537500813665
H	0.31580172027280	2.55881153204202	2.59316194036105
H	1.49742063691743	3.50959096078315	1.67254409854274
H	3.93536230815489	1.69059275114013	2.54766307612084
H	3.93560301358223	0.66896229978856	1.09208764778592
H	3.70335753363265	2.43492021060122	0.96469985168415
C	1.53518475264943	1.89646481236311	-1.36570083132912
C	0.49403138257345	1.55036998878604	-2.44607513630351
C	2.92884472088653	1.53864869800881	-1.90000329972112
C	1.47105463380347	3.40098149732637	-1.06108406542542
H	0.45384599731501	0.47443571997941	-2.64455672675922
H	-0.50909556341428	1.90766430323349	-2.19385338710393
H	2.98862119941271	0.49384994603552	-2.20927080550946
H	3.72092503556275	1.71714836510930	-1.17071532347929
H	3.11996479686794	2.17621749889929	-2.77193887639141
H	1.58521934600667	3.94287290847496	-2.00718143119929
H	0.51638174152380	3.70889981185507	-0.62312364007276
H	2.28240137985626	3.72041897481649	-0.40177568642646
C	1.28218720210266	-0.89614308729965	-0.23571041079885
N	0.13752138572404	-1.45392504618742	-0.54660790428104
S	2.86444020309127	-1.49109722381885	-0.23758257040078
C	0.04727603017211	-2.81876517515326	-0.90506976195352
C	0.47500460492730	-3.83039387448729	-0.04089147592680
C	0.27340466353141	-5.15957188592182	-0.38463649082897
C	-0.34377510202489	-5.49530540901854	-1.58820427022074
C	-0.76522740673610	-4.48790200466634	-2.44906921784213
C	-0.57761127820454	-3.15232120176818	-2.10886179886530
H	0.95044030397140	-3.56591505589483	0.89698518004997
H	0.60064657939240	-5.94161617471816	0.29445428766419
H	-0.49689611842555	-6.53765003674882	-1.85086352229636
H	-1.24615651467651	-4.73885091575943	-3.39003910219616
H	-0.90591657819106	-2.36118338052813	-2.77713178345319

Point Group: C_1, Symmetry Number: 1
Total Enthalpy ... -2809.59223533 E_h
Final Gibbs free energy ... -2809.69510933 E_h
0: 0.00 cm**-1
1: 0.00 cm**-1
2: 0.00 cm**-1
3: 0.00 cm**-1
4: 0.00 cm**-1
5: 0.00 cm**-1
6: 19.86 cm**-1
7: 30.98 cm**-1
8: 33.91 cm**-1
9: 36.01 cm**-1
10: 42.26 cm**-1
11: 48.40 cm**-1
12: 50.80 cm**-1

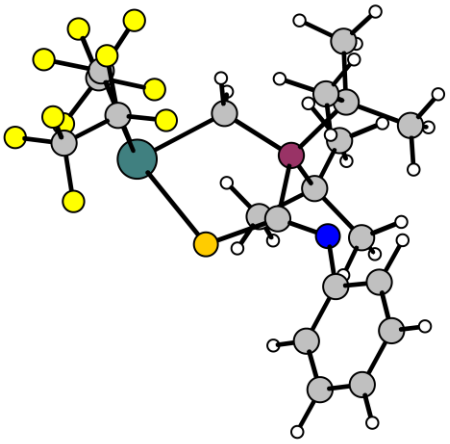

Figure S67 Molecular structure of the regioisomer of **5** with S···Sb and C···P contacts obtained from geometry optimisation at r^{2}SCAN-3c-D4/def2-TZVPP level of theory.

59
Coordinates from ORCA-job jonas_flp_phncs_sc_r2scan-3c_def2-tzvpp_d4

C	4.30901079484402	-1.59231568269537	0.23872583519138
C	3.20597182140592	-1.46050897356785	-0.61793242196131
C	3.22516050257150	-2.11690550347514	-1.85757451574971
C	4.32074819860226	-2.89356759449793	-2.21360670678373
C	5.39725427333768	-3.04847234393811	-1.34406959204026
C	5.38462271985816	-2.39430895686332	-0.11419050648015
N	2.19232360069179	-0.58926097946517	-0.20401921301779
C	0.93114670231832	-0.74965344624723	-0.42805519451434
P	0.01093677139377	0.74830635902548	0.15222477234264
C	0.67983172682718	1.29360512378331	1.83229239932900
C	2.04511632796538	1.98638478898189	1.72927968623639
C	-1.73688976697781	0.39218357260698	0.39816308779229
Sb	-2.71006560192801	-1.16931086610378	-0.83016160727253
C	-4.71038385061037	-0.24009291692267	0.01324094895066
C	-5.27960572806715	0.79944300341974	-0.96300103579689
F	-4.36448884704927	1.79363683038461	-1.14524769160944
C	-2.73433503128865	-2.57040375204728	0.97018687807733
C	-3.08342314708542	-4.01898764583140	0.58263174187533
F	-3.09935149482798	-4.83520646482096	1.64812247018411
C	0.13684506388681	2.01872664077404	-1.23371810920052
C	-0.33962542800271	3.39923774289340	-0.76011522919718
F	-3.63890311834816	-2.16882305776314	1.90889515709694
F	-1.51521958524792	-2.58554724432092	1.58711684184095
F	-4.30024555188371	-4.04698146948482	0.00615903110419
F	-2.18659022942474	-4.49010533047006	-0.30138976291935
F	-5.66940846979265	-1.21041015024654	0.15105437274269
F	-4.59697409217814	0.40299335573751	1.22786561533529
F	-5.50870448127183	0.23670670817532	-2.16389132652620
F	-6.41853230133926	1.37122912860707	-0.54229878063321
C	1.57825166954156	2.10851364700058	-1.75623040271247
C	-0.77393169569292	1.54015502619662	-2.37903542576301
C	0.81120700907954	0.01450296264703	2.67926610502381
C	-0.32176143812985	2.24241804277001	2.51505034381925
S	0.02068594232035	-2.00774222289821	-1.18033856087583
H	-2.31887980312843	1.30727928118665	0.25936604682598
H	-1.89242659369646	0.06241746911694	1.42892703365948
H	1.90299033866023	1.16810720226311	-2.20732794156520
H	2.29763052532385	2.37509073773492	-0.97943461469810
H	1.60602084008360	2.88687542322865	-2.52788648136351
H	-0.61795243844771	2.20734377682130	-3.23490917833073
H	-1.83473603486681	1.59331147704519	-2.11595193202636
H	-0.53433885148397	0.52102007244675	-2.69900590646939

H	-0.37629233195934	4.06293474094047	-1.63203057741742
H	0.34438825340370	3.84786420487736	-0.03513120914403
H	-1.34631617308977	3.37302517129721	-0.33042820141245
H	1.09799957931376	0.31053630521535	3.69507723134700
H	1.57806006241875	-0.65383883045304	2.28241277241274
H	-0.12920137104134	-0.54152038105443	2.75318385980655
H	-0.48346588490855	3.16535710740090	1.95394230343593
H	0.09697787863651	2.51965895262493	3.48954461801883
H	-1.29138970696441	1.77267067239930	2.69860044473075
H	1.97665031832039	2.95881931626544	1.23287130451773
H	2.77208788563977	1.36280873134513	1.20569108097655
H	2.40752686018800	2.16696169062891	2.74864016988952
H	2.39026548428264	-2.00735819725946	-2.53975690624596
H	4.32858247764411	-3.38999733057465	-3.18000052354156
H	6.24320110618593	-3.66806199881168	-1.62597521372174
H	6.22310100866722	-2.50061941876433	0.56823324412952
H	4.29884340532064	-1.06012470726458	1.18594957229755

Point Group: C_1, Symmetry Number: 1
Total Enthalpy ... -2809.59244734 E_h
Final Gibbs free energy ... -2809.69682037 E_h
0: 0.00 cm**-1
1: 0.00 cm**-1
2: 0.00 cm**-1
3: 0.00 cm**-1
4: 0.00 cm**-1
5: 0.00 cm**-1
6: 7.22 cm**-1
7: 17.06 cm**-1
8: 21.53 cm**-1
9: 33.68 cm**-1
10: 35.48 cm**-1
11: 38.21 cm**-1
12: 43.40 cm**-1

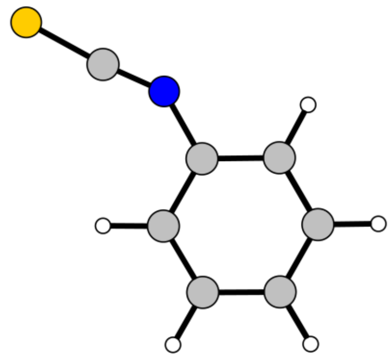

Figure S68 Molecular structure of PhNCS obtained from geometry optimisation at r²SCAN-3c-D4/def2-TZVPP level of theory.

14
Coordinates from ORCA-job jonas_phncs_r2scan-3c_def2-tzvpp_d4

C	-0.72217207816599	0.20052874956955	0.00000009187300
C	-0.66267387748478	-1.19508987361464	0.00000006541992
C	0.57418021731090	-1.82668006655011	-0.00000002830491
C	1.74839452061109	-1.07892541315479	-0.00000002078098
C	1.68203114605635	0.31304144433388	0.00000002529600
C	0.45464420006859	0.95985783416700	0.00000005712045
H	-1.58607352898607	-1.76484430993533	0.00000005114447
H	0.61969751689114	-2.91157688154262	-0.00000008142008
H	2.71255150957671	-1.57760381043363	-0.00000008097735
H	2.59474584631231	0.90171046741681	0.00000000176986
H	0.39117020201402	2.04389640047577	0.00000006599034

N	-1.96288477327010	0.81857006665575	0.00000001283596
C	-2.50599163128751	1.88848848872835	-0.00000004210573
S	-3.33761946964667	3.22862710388400	-0.00000011786096

Point Group: C_s, Symmetry Number: 1
Total Enthalpy ... -722.48600520 E_h
Final Gibbs free energy ... -722.52699700 E_h
0: 0.00 cm**-1
1: 0.00 cm**-1
2: 0.00 cm**-1
3: 0.00 cm**-1
4: 0.00 cm**-1
5: 0.00 cm**-1
6: 54.69 cm**-1
7: 63.15 cm**-1
8: 235.37 cm**-1
9: 359.46 cm**-1
10: 411.33 cm**-1
11: 411.52 cm**-1
12: 447.02 cm**-1

Interacting Quantum Atoms (IQA) and Quantum Theory of Atoms in Molecules (QTAIM)

Free molecular structures of **2** and **6** were optimised at the PBE0-D3BJ/def2-QZVPP[30–32] and r^2SCAN-3c[18] levels of theory starting from the determined XRD structures. These calculations were performed using the Orca 5.0.3 and 5.0.4 program packages[27] with the settings *TightSCF*, *DefGrid3* and *TightOpt*. The RIJCOSX accelerating approximation[33] was also used. Closed-shell singlet ground state electronic solutions were calculated in all cases.

For the structures obtained, electronic wave functions were calculated at the PBE0/def2-TZVPP level of theory using the Gaussian 16 package.[34] These were further processed by the AIMAll software[35] for analyses using quantum theory of atoms in molecules (QTAIM)[36] and interacting quantum atoms (IQA).[37] For comparison, similar calculations were performed for other molecular systems: Me_2Sb–SMe, LiF and Xe_2. All results are summarised in Tables S2 and S3 and the corresponding plots are shown in Figures S59–S64.

Summarising, for the Sb(1)–S(2) interaction in **2** the following conclusions can be made:

1. Stabilising,

2. Weak,

3. Polar,

4. Partially covalent,

5. Essentially due to the electron exchange-correlation effects.

The main results for **6** are as follows:

1. Au–P are typical polarised bonds with large covalent character.

2. Au–Cl is a weak polarised bond with large covalent character. Its stabilisation energy is ~3 times less than that for Au–P.

3. Sb–Cl are strongly stabilising primarily ionic interactions. The stabilisation energy is the same as for bonded Au–P.

4. P···Cl are purely ionic and also stabilising (twice larger than Au–Cl!), although without bond critical points and bond paths!

5. Notably, there are other, weaker bond paths Cl···F and Cl···H.

Table S2 Results of QTAIM and IQA analyses for selected atom pairs in several compounds. All quantities are in atomic units, unless otherwise stated. Atomic charge q, electron delocalisation index f_{AB}, electron density in bond critical point ρ_{BCP} [e Å^{-3}], Laplacian of the electron density in bond critical point $\nabla^2\rho_{BCP}$ [e Å^{-5}], total interaction energy between atoms A and B in IQA analysis E_{int}^{AB}, $V_{eeX}^{AB}/E_{int}^{AB}$ electron exchange-correlation contribution in E_{int}^{AB} in percent. Negative values indicate different signs of V_{eeX}^{AB} and E_{int}^{AB}. For atom numeration see Figure S59; note that the numeration in the article and in the supporting information is partly different.

Compound, atom pair A–B	q(A)	q(B)	f_{AB}	ρ_{BCP}	$\nabla^2\rho_{BCP}$	E_{int}^{AB}	$V_{eeX}^{AB}/E_{int}^{AB}$, %
**2**, Sb(1)–S(2)	1.22	0.05	0.39	0.27	0.96	$-8.20\cdot10^{-2}$	84
**2**, Sb(1)–C(19)	1.22	−1.03	0.80	0.67	1.69	$-5.49\cdot10^{-1}$	32
**2**, S(2)–C(48)	0.05	−1.28	1.59	1.55	−9.64	$-5.95\cdot10^{-1}$	67
**2**, Sb(1)···P(4)	1.22	2.22	0.03	-	-	$4.69\cdot10^{-1}$	−1
Me_2Sb–SMe, Sb–S	1.10	−0.43	0.97	0.61	1.20	$-3.25\cdot10^{-1}$	57
LiF, Li–F	0.92	−0.92	0.22	0.54	16.87	$-3.27\cdot10^{-1}$	16
Xe_2, Xe···Xe	0.00	0.00	0.05	0.02	0.21	$-5.78\cdot10^{-3}$	97

Table S3 Results of QTAIM and IQA analyses for selected atom pairs in molecule **6**. All quantities are in atomic units, unless otherwise stated. Atomic charge q, electron delocalisation index f_{AB}, electron density in bond critical point ρ_{BCP} [e Å^{-3}], Laplacian of the electron density in bond critical point $\nabla^2\rho_{BCP}$ [e Å^{-5}], total interaction energy between atoms A and B in IQA analysis E_{int}^{AB}, $V_{eeX}^{AB}/E_{int}^{AB}$ electron exchange-correlation contribution in E_{int}^{AB} in percent. For atom numeration see Figure S62; note that the numeration in the article and in the supporting information is partly different.

Atom pair A–B	q(A)	q(B)	f_{AB}	ρ_{BCP}	$\nabla^2\rho_{BCP}$	E_{int}^{AB}	$V_{eeX}^{AB}/E_{int}^{AB}$, %
Au(1)–P(4)	−0.06	1.47	0.90	0.73	1.66	$-1.97\cdot10^{-1}$	105
Au(1)–P(5)	−0.06	1.44	0.89	0.72	1.66	$-1.93\cdot10^{-1}$	105
Au(1)–Cl(92)	−0.06	−0.67	0.34	0.22	2.19	$-5.88\cdot10^{-2}$	98
Sb(2)–Cl(92)	1.22	−0.67	0.29	0.19	1.20	$-1.94\cdot10^{-1}$	25
Sb(3)–Cl(92)	1.21	−0.67	0.27	0.18	1.13	$-1.92\cdot10^{-1}$	23
P(4)···Cl(92)	1.47	−0.67	0.02	-	-	$-1.32\cdot10^{-1}$	2
P(5)···Cl(92)	1.44	−0.67	0.03	-	-	$-1.35\cdot10^{-1}$	2

Table S4 Optimised Cartesian coordinates of **2**.

Sb	7.26069120955552	6.70617307251217	2.97844295733453
S	6.26782785570409	5.23424012826055	5.24551637445319
S	7.67789017883385	2.88571662517114	6.44207536515756
P	7.94866993603683	3.43620286448896	3.56570359258784
F	4.93486521649706	7.11232468523563	1.07637708361372
F	4.43537288424945	5.67948940169329	2.62460276940982
F	4.39391155183667	7.71474650718133	4.46911572249862
F	3.07817087561091	8.04929627487239	2.79422817133999
F	4.94743625355557	9.12058261041200	2.92734751585099
F	7.33967844387640	6.48580073147103	-0.16909069509586
F	9.26346173212359	6.72649969572382	0.82876440386320
F	8.70974761251960	9.32145080309904	1.34189272374594
F	6.79026567633047	9.15579505668264	0.36845952368288
F	8.59925505804170	8.82455307642305	-0.75525687766668
C	5.08202053631142	6.84324288317814	2.38861302757735
C	4.34976397652754	7.95202796720120	3.16098737425411
C	7.95884819716866	7.16374144177519	0.82529706548204
C	8.01437196897068	8.64097275613622	0.42649707216505
C	7.40924214945600	4.62803616675127	2.34522413287108
H	6.38846637836204	4.37480132590877	2.06416020565505
H	8.03266937909162	4.56477164912953	1.45774852222764
C	9.81015292531151	3.51704998031211	3.63472496339187
C	10.16186797598182	4.74268472199343	4.48280641444025
H	11.24642430844750	4.86762546552719	4.46162102648466
H	9.72381212654772	5.66398911401443	4.09363989068735
H	9.84867704170497	4.62056719592181	5.51802179911296
C	10.38497320026284	3.70088510211160	2.22930626333765
H	10.11845780766081	4.65866296214628	1.78724515266678
H	11.47353177413165	3.67380929364696	2.31131916762814
H	10.09097031352311	2.90394897564122	1.54672123441096
C	10.43362948939839	2.27617978441495	4.26933259339283
H	10.39872466548359	1.41978201663497	3.59839775013482
H	11.48560525993605	2.49655952816392	4.46380885818038
H	9.95738150711128	2.01470140110406	5.21225201591461
C	7.16952780628392	1.82043142972172	3.04267280982121

C	5.66764539399812	1.97702942834306	3.30456724940086
H	5.22962925790593	2.84701570308903	2.81591085830603
H	5.16564803804512	1.09130215905007	2.91030724956687
H	5.45512640253202	2.04236325054047	4.37058467134527
C	7.41748253897430	1.60723325393275	1.54872681383021
H	8.47581961038040	1.47626507322187	1.32368002523984
H	6.90591537831836	0.68973245011581	1.25054507569762
H	7.02676256254614	2.41602583089053	0.93310191896890
C	7.66782387270366	0.59854237316540	3.80929852023270
H	8.68418099997337	0.32679641161413	3.53619210453289
H	7.61982840839650	0.74581053392666	4.88610643988083
H	7.02063536073471	-0.24089206919643	3.54502973158392
C	7.25873890304559	3.89039290664413	5.19830337080115

Table S5 Optimised Cartesian coordinates of **6**.

Au	3.30533015814677	6.94081540112967	4.83992743043433
Sb	5.35353389933368	9.33451817518781	7.52510968628018
Sb	3.33384019609624	10.68033881158383	3.96614527899271
P	3.82588463322386	6.21210189466914	7.00368385601110
P	2.57005857641383	7.58499626132714	2.70316140699873
F	6.57412541949649	9.72941898570595	10.46079358412020
F	4.71985323458440	8.54158855543514	10.48872083871619
F	3.29448751215505	10.76796772839221	9.75960719348659
F	5.13308538774793	11.94381946682484	9.67845478396745
F	4.48214888187557	11.06206879931002	11.56321397124453
F	8.02668608699668	8.40465127721463	8.66417891767865
F	7.99012185524944	8.24751376838790	6.48632982288306
F	8.11115711590195	10.95351801864887	6.23590428586511
F	8.03390730120302	11.19809649120810	8.40118595267908
F	9.72360765295331	10.17184034935797	7.47864438456401
F	2.43121687569656	12.96475639620672	1.92438081176337
F	0.90700502198858	11.40856538626350	2.17949848218628
F	0.24707089900621	13.85874866277609	3.26714638953311
F	1.98793643853707	13.75108564275965	4.57467714933990

F	0.42161030061071	12.23511816292579	4.71241564553190
C	5.35677067298503	9.60140879304967	9.86428332679862
C	4.55706932228401	10.86315243571227	10.23701002406177
C	7.61589342839969	9.04181242159306	7.52778092304021
C	8.39631738867613	10.36418679792213	7.40688622220498
C	5.20922257097070	7.14462151445389	7.76557322012719
H	6.11797456868596	6.79412826226592	7.26570799984742
H	5.29597890561213	6.91708232191819	8.83211214215919
C	4.51039684439799	4.44195107818148	6.93712433918265
C	5.47076897001874	4.40980720687497	5.73390348122699
H	4.92837707462978	4.55528888693641	4.79337893594933
H	5.96053537918968	3.42886031307525	5.69983493643972
H	6.25522872381991	5.17163293095999	5.79026550541723
C	5.28162140801673	4.04879185244235	8.20407767289346
H	6.16554060572159	4.67377559003956	8.35767622541797
H	5.62788267084957	3.01376632065397	8.09358414237782
H	4.66584924303411	4.09632943745975	9.10515644506495
C	3.37970781538722	3.43964632798735	6.67811407312920
H	2.74312240918741	3.29991108750787	7.55628577825983
H	3.82152441587835	2.46535748136849	6.43544781431532
H	2.75484682892619	3.74570437468135	5.83116094759540
C	2.33437589779120	6.40963406641449	8.14673049951527
C	2.53811517771076	5.78276763091198	9.52997055043146
H	2.56463835996685	4.69032926439673	9.48378125375245
H	1.69408482472938	6.06301627264999	10.17222199132854
H	3.44920653871232	6.14038940453798	10.01936231452032
C	1.08169474049409	5.82254423265676	7.47915802013096
H	0.90818843247615	6.28017872387503	6.49947421388369
H	0.21537037461210	6.03704929264274	8.11657800973912
H	1.13574687330366	4.74056842395094	7.34590367552991
C	2.12048496458102	7.92463023777170	8.29897614081177
H	2.89617675284972	8.38486795511380	8.91331052665458
H	1.16506853090266	8.09863005633832	8.80839526207840
H	2.07749516965976	8.43473012913318	7.32806561956704
C	1.87527574449120	12.13872949882404	2.85171936858677
C	1.11537551825367	13.02063756016279	3.85695931232684
C	3.13692688960703	9.28233207215120	2.27727893832358

H	2.51637596279944	9.73570527698928	1.49981777918122
H	4.15494788914126	9.17902857963799	1.89586931035726
C	3.35984745239797	6.52901733944184	1.33840791600727
C	4.84968210827776	6.41239736388575	1.70751131064981
H	5.34715503375566	7.38159977444205	1.80722053059045
H	5.36056912422985	5.85107270909162	0.91558232690909
H	4.98826140998520	5.87869200414200	2.65341061811236
C	2.75020935083120	5.12253411581055	1.32238389237285
H	2.70332719968500	4.68649175663334	2.32705202711999
H	3.38098243969933	4.47310800969436	0.70330679769521
H	1.74763077685378	5.11534361195881	0.88645501245178
C	3.23524321737195	7.16800757972635	-0.05110494988404
H	2.19635983861063	7.30569013340595	-0.35994683785445
H	3.71371063055128	6.50506382193296	-0.78260538274849
H	3.74218464856684	8.13527779380360	-0.10771943746554
C	0.67763952589747	7.60077242466455	2.68194220802553
C	0.13850586129509	6.22147007476550	3.09327469757161
H	0.27514531376301	5.46493385890944	2.31927103028434
H	-0.93843882290970	6.31192066622220	3.27992239797731
H	0.61618013978182	5.86660837631349	4.01350982576687
C	0.07870393106914	8.01634820947292	1.33489233433034
H	0.46813619532962	8.97509802827328	0.98000400039314
H	-1.00657024514093	8.12866254214936	1.44848850454900
H	0.24878016978910	7.25783817691092	0.56546649094761
C	0.25916214254850	8.60262230650550	3.77244990236157
H	0.68725581289380	8.33639538311603	4.74651510207877
H	-0.83331420855009	8.58262531770835	3.86717213285632
H	0.53825570764898	9.62877592758150	3.52888551629213
F	4.73139195383963	13.23580663845966	3.28816315686418
F	6.11769438175934	11.79481958168278	4.17251889071775
F	6.25607616846861	10.41365948612558	1.82779194537793
F	4.75193518807144	11.70635683071900	0.92683142684832
F	6.66545569075726	12.53447177870062	1.55909915906770
C	5.11244370358951	11.92379189010050	3.26733339497319
C	5.71196217464724	11.64269875145218	1.87125267851110
Cl	5.55759064866349	8.73251538763809	4.59254456574314

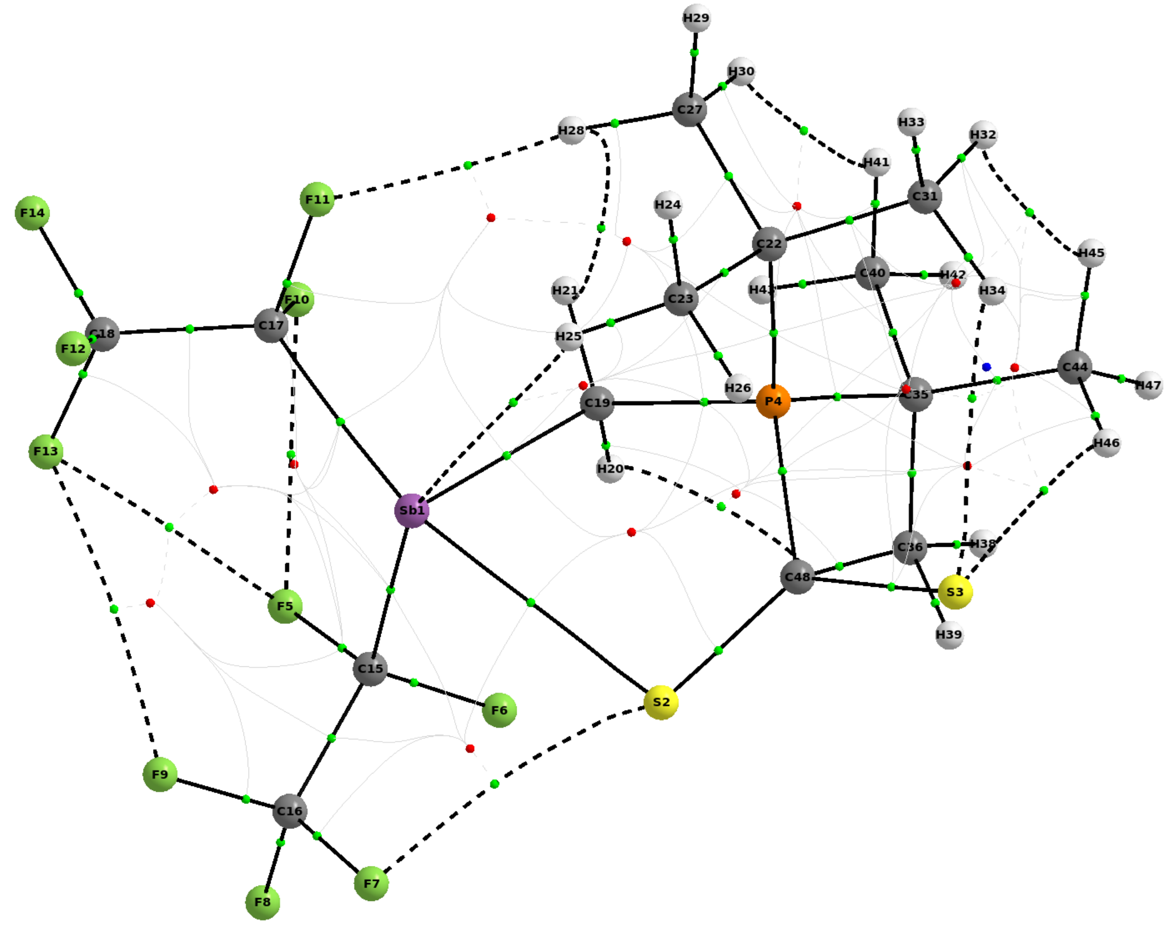

Figure S69 Bond paths and critical points in **2**. Internal working numeration of atoms is shown.

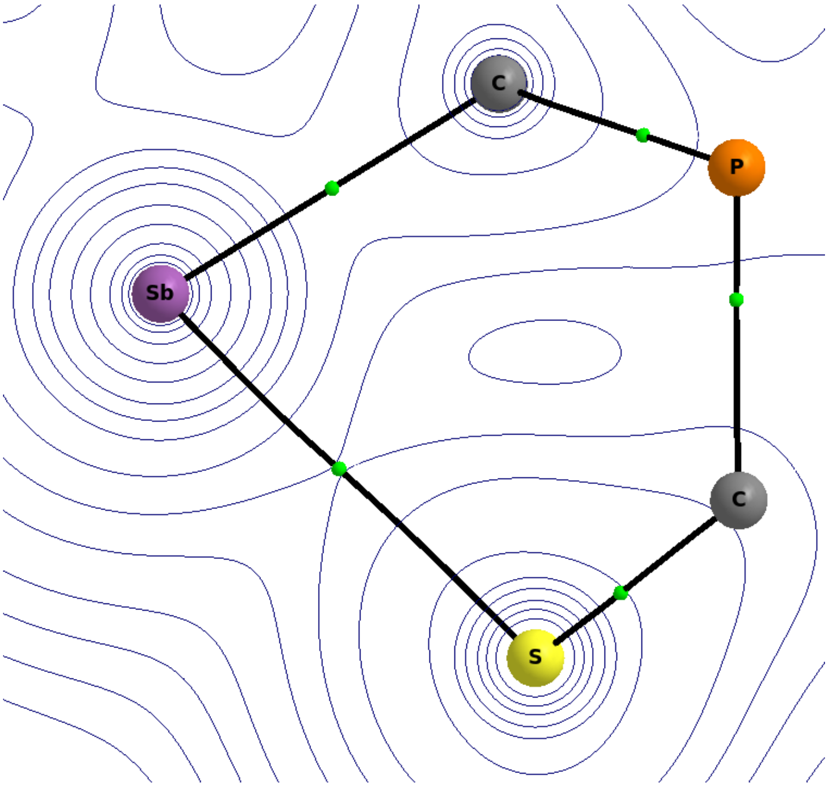

Figure S70 Contour plot of electron density in the plane C(19)–Sb(1)–S(2) of **2**.

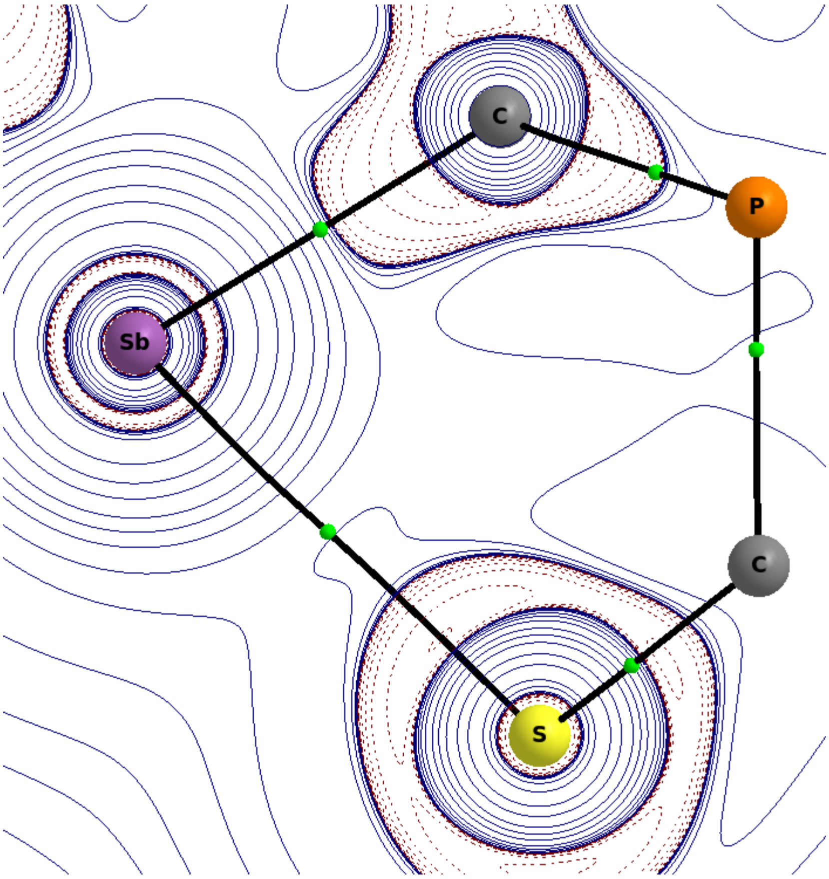

Figure S71 Contour plot of the Laplacian $\nabla^2\rho$ (positive and negative isovalues are full blue and dashed red lines, respectively) of the electron density of **2** in the plane C(19)–Sb(1)–S(2).

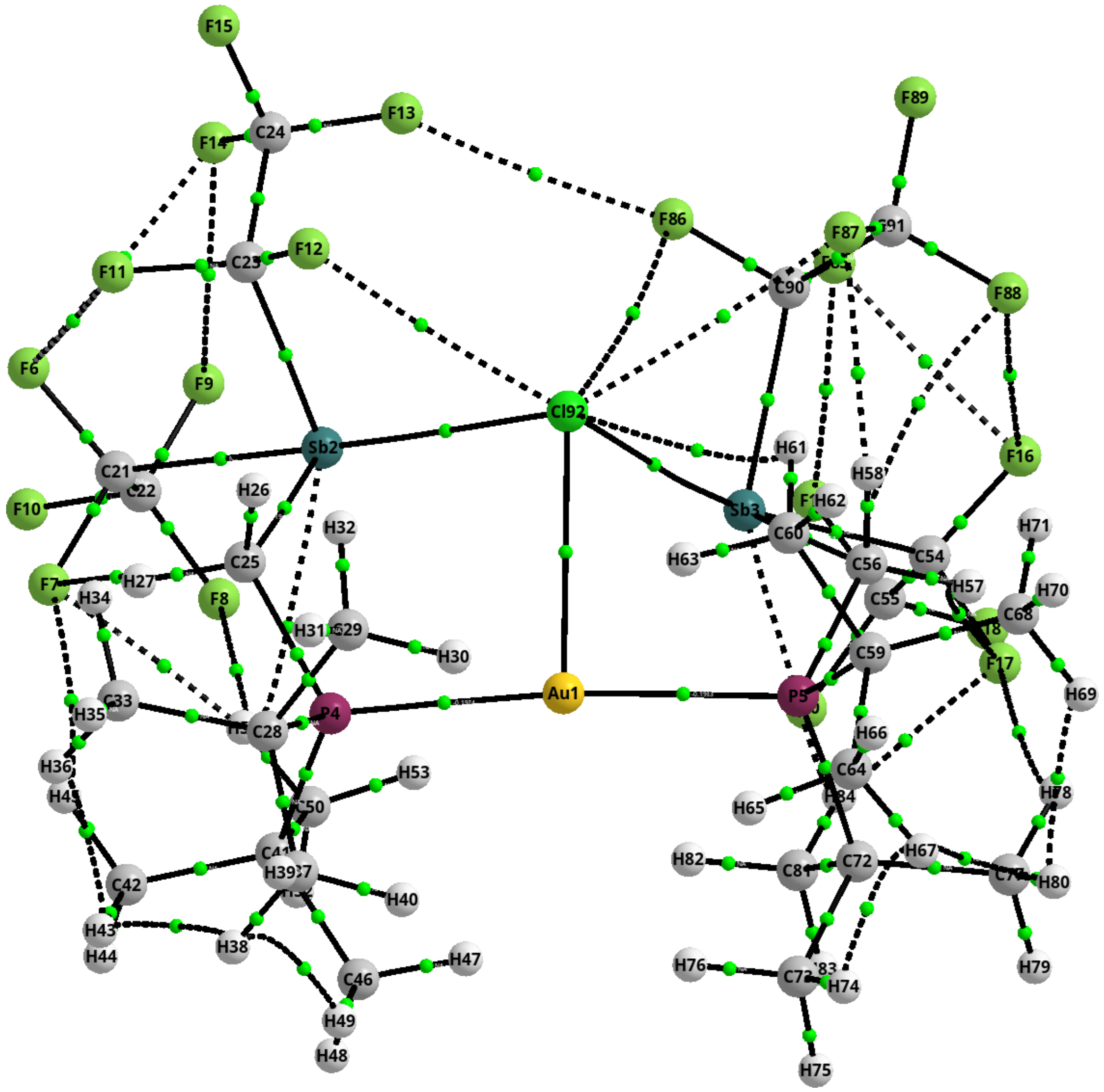

Figure S72 Bond paths and critical points in **6**. Internal working numeration of atoms is shown.

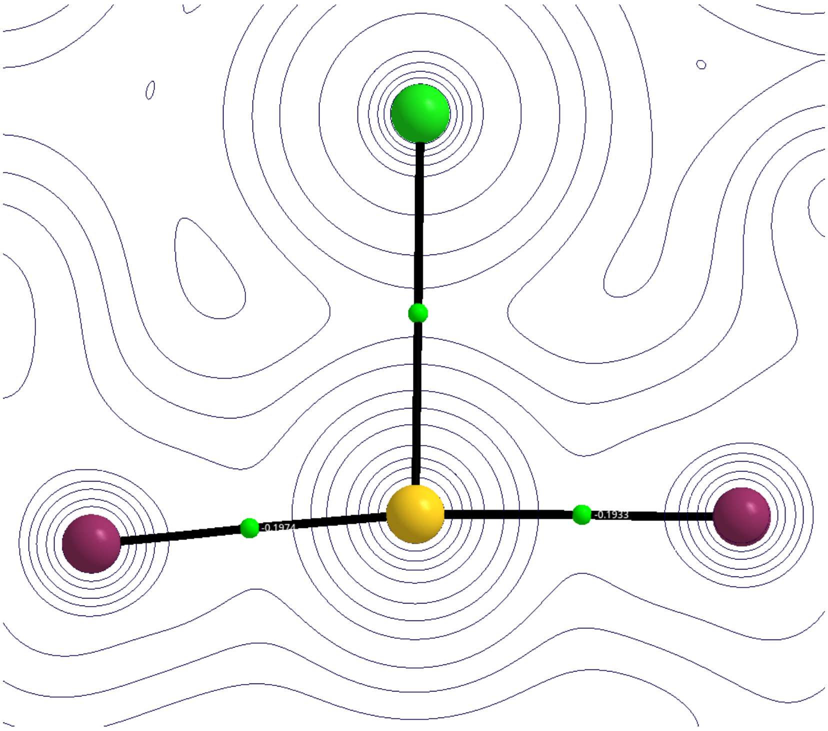

Figure S73 Contour plot of electron density in the plane Cl(92)–Au(1)(P4)–P(5) of **6**.

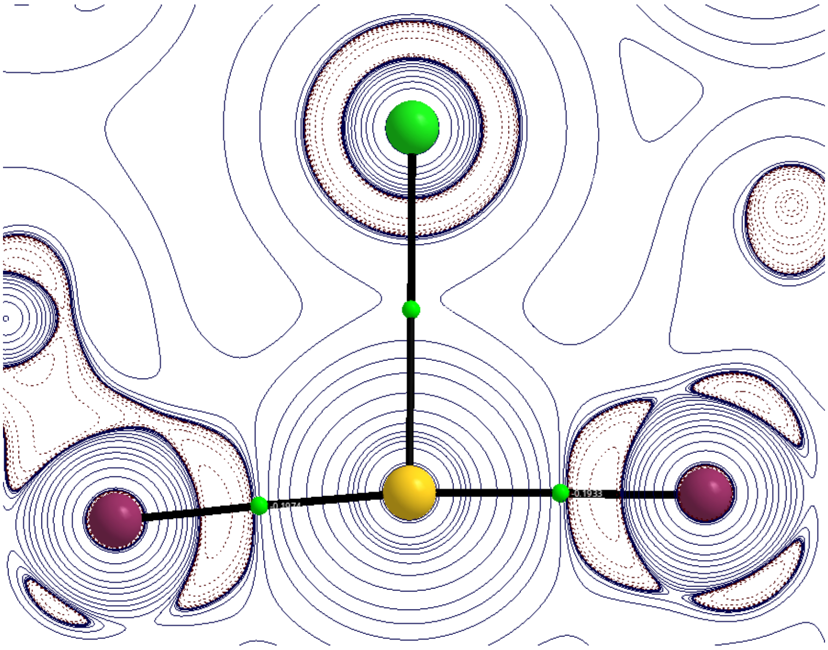

Figure S74 Contour plot of Laplacian $\nabla^2\rho$ (positive and negative isovalues are full blue and dashed red lines, respectively) of the electron density of **6** in the plane Cl(92)–Au(1)(P4)–P(5).

Fluoride Ion Affinity (FIA) of 1

The fluoride ion affinity of **1** was computed as described by Greb et al.[38] Geometry optimization and vibrational analysis was performed with the composite functional PBEh-3c.[22,31,39–41] The thermal corrections obtained were combined with the electronic energy calculated at the DSD-BLYP(GD3BJ)/def2-QZVPP[31-32,40–43] level of theory (method L3). To set up an isodesmic reaction equation for FIA computation the Me_3SiF/Me_3Si^+ system was used with its anchor value of 952.5 kJ mol^{-1}.[38] All calculations were performed with Orca 5.0.2[24–27] and the energetic data is summarized in Table S6. The cartesian coordinates of the optimized molecular structures are included below. Attempts to calculate the hydride ion affinity failed.

Table S6. The total correction to the electronic energy and thermal enthalpy correction obtained from PBEh-3c geometry optimization and vibrational analysis for **1**, [**1**–F]⁻ and the reference system Me_3SiF/Me_3Si^+ as well as the electronic energy calculated at the DSD-BLYP(GD3BJ)/def2-QZVPP level of theory.

	Total correction to electronic energy / E_h	Thermal enthalpy correction / E_h	Electronic energy / E_h	Enthalpy / E_h	FIA / kJ mol⁻¹
Me_3SiF	0.12525603	0.00094421	−508.8756731913	−508.74947295	
Me_3Si^+	0.11999520	0.00094421	−408.6884443066	−408.56750490	
**1**	0.36630269	0.00094421	−2086.4277560434	−2086.06050914	278
[**1**-F]⁻	0.36824433	0.00094421	−2186.3547241650	−2185.98553563	

Me_3SiF:

14
Coordinates from ORCA-job jonas_me3sif_pbeh-3c_def2-msvp

```
C   0.03223021586528   -0.03689916274419   -0.17684604104881
Si  0.03539906992482    0.03342238318567    1.69323025530693
C   1.77730188883268    0.09847973555284    2.37396100304842
H   0.50421189847945    0.84773661614874   -0.60767339131342
H  -0.98157999809898   -0.08985000911845   -0.57557955197408
H   2.29842062956767    0.99573877991475    2.03537511713709
H   2.36878362771907   -0.75970544186495    2.05263599927881
H   0.57574628400275   -0.90701345253884   -0.54716360934047
H   1.78550746751294    0.11246152814377    3.46467451093120
```

C -0.99040727852269 1.46880461299345 2.31958422886217
H -1.02140942014689 1.50225968005432 3.40930952520038
H -0.57863432725119 2.42079360242026 1.97916668781449
H -2.02004335357641 1.41740254836160 1.96336597455852
F -0.64977270430850 -1.34371342050898 2.22794429153873
Point Group: C_1, Symmetry Number: 1
0: 0.00 cm**-1
1: 0.00 cm**-1
2: 0.00 cm**-1
3: 0.00 cm**-1
4: 0.00 cm**-1
5: 0.00 cm**-1
6: 134.86 cm**-1
7: 155.32 cm**-1
8: 156.16 cm**-1
9: 189.59 cm**-1
10: 191.52 cm**-1
11: 233.68 cm**-1
12: 280.95 cm**-1

Me_3Si^+

13
Coordinates from ORCA-job jonas_me3si+_pbeh-3c_def2-msvp
Si -0.00206938228384 -0.00005263858114 0.00231734055847
C -0.01525883980758 0.09019991428207 1.83259635241751
C 1.58193594831287 -0.22023344877842 -0.89053842945871
C -1.56774959213409 0.13215893411611 -0.94059018784654
H 0.32777202921434 -0.86080438292304 2.25507576475128
H 0.69496648519360 0.84111283246969 2.19047584582851
H -0.99773995322048 0.30924258670431 2.24715253468615
H 2.44989840651760 -0.22859338736725 -0.23358974344146
H 1.71591536966484 0.56898809612536 -1.63683823192286
H 1.57070159364161 -1.15750167699511 -1.45685401107184
H -1.99750931559327 1.13149486007440 -0.80885537466944
H -1.44237321072027 -0.04385197035459 -2.00753381493732
H -2.31848953878533 -0.56215971877239 -0.55281904489374
Point Group: C_1, Symmetry Number: 1
0: 0.00 cm**-1
1: 0.00 cm**-1
2: 0.00 cm**-1
3: 0.00 cm**-1
4: 0.00 cm**-1
5: 0.00 cm**-1
6: 41.94 cm**-1
7: 46.46 cm**-1
8: 79.90 cm**-1
9: 214.11 cm**-1
10: 214.71 cm**-1
11: 226.01 cm**-1
12: 619.94 cm**-1

$(F_5C_2)_3SbCH_2P(CH_3)_3$ (**1**)

45
Coordinates from ORCA-job jonas_flp_pbeh-3c_def2-msvp
Sb -0.65331955228676 -0.67590733231297 -0.37045133892419
P 2.52505341822511 0.30210783800830 -0.35597802766942
F -1.70481098596768 1.90809749769698 0.85052187593805
F -0.16766530498031 2.22535870039469 -0.65779365025363
F -1.86864278933799 1.38574782223146 -2.59694643261188

F	-2.64200425029697	3.05160347028161	-1.46320488646787
F	-3.41948689719190	1.06525253607587	-1.13475564870596
F	-1.37425438457679	-0.56012226084692	2.58384894058765
F	-1.13102627419034	-2.55358014638939	1.72992037157762
F	-3.50690797706338	-2.19499375958161	0.36182307770542
F	-3.78533295192762	-0.31576166408017	1.39102390565569
F	-3.64731841367399	-2.14871728909035	2.51503606908143
C	-1.25264248253492	1.47894793353724	-0.34535623002999
C	-2.31871475508655	1.76719462385291	-1.40104067392055
C	-1.66882008746663	-1.31996288847613	1.50982316355538
C	-3.18393373728098	-1.49842601078651	1.45375026552218
C	1.15597886214797	-0.28628394987643	0.75911261046907
H	0.90811320654279	0.49477004765405	1.47583067606565
H	1.40291442281892	-1.17907424770468	1.33454396666466
C	3.36640544158483	-1.33262498006591	-0.83609134075038
C	2.57750170086944	-1.85877863822383	-2.04229325491660
H	3.06075651861730	-2.75633813680764	-2.43776273220518
H	1.55564354395163	-2.14352748826439	-1.78578583865624
H	2.52478083006954	-1.12397793975892	-2.84624077940171
C	3.40381903924019	-2.42402495339818	0.23107405529915
H	2.40847066842139	-2.78644007281953	0.49066391328579
H	3.95683463124976	-3.28846846988813	-0.14723071927105
H	3.89441243203281	-2.10645013946429	1.14936662678587
C	4.79006497116080	-1.04375534184040	-1.31409666770247
H	5.46635706931107	-0.80901084811127	-0.49248079231786
H	5.19292679457949	-1.92706962591632	-1.81604135678425
H	4.82549136510703	-0.21956173072714	-2.02879047742450
C	3.60517102729692	1.25979996401904	0.87531274906519
C	2.70912165887142	2.34089501497445	1.49547756656458
H	2.01929594442698	1.94356164706996	2.23928491763087
H	3.33494247142805	3.07220431060907	2.01209894085528
H	2.12774887361668	2.88017764273056	0.74648608923382
C	4.23121088021329	0.43394849382755	1.99345645895803
H	4.97686807090042	-0.26664475080888	1.61854544823670
H	4.73729598461656	1.09064405963759	2.70683250840335
H	3.48510994348868	-0.13403057902240	2.55212909760619
C	4.69338979026329	1.99164030718105	0.08223377020859
H	5.42752251722330	1.32001754760780	-0.35657828310012
H	4.26384159548372	2.58851040721245	-0.72353853246137
H	5.23499217010310	2.67102237965979	0.74553759861892

Point Group: C_1, Symmetry Number: 1

0: 0.00 cm**-1
1: 0.00 cm**-1
2: 0.00 cm**-1
3: 0.00 cm**-1
4: 0.00 cm**-1
5: 0.00 cm**-1
6: 16.90 cm**-1
7: 23.43 cm**-1
8: 34.09 cm**-1
9: 42.43 cm**-1
10: 46.84 cm**-1
11: 51.20 cm**-1
12: 57.52 cm**-1

$[(F_5C_2)_3Sb(F)CH_2P(CH_3)_3]^-$ ([**1**-F]$^-$)

46
Coordinates from ORCA-job jonas_flp_f-_pbeh-3c_def2-msvp

C	1.16580932035303	0.16451351323966	0.45380491724807

P	2.70412651855450	0.17874366425675	-0.58505797327181
C	3.82808435437397	1.36357518578013	0.38574748272766
C	5.12637627418646	1.57564151582678	-0.39986534019276
C	3.33363477697287	-1.61035629788894	-0.37992889543483
C	4.82549165706601	-1.69342897530967	-0.70292919796550
Sb	-0.60678242348704	0.35039754518225	-0.81639231273067
C	-1.38144975456885	-1.57663610470088	0.44004387540165
C	-2.74575007670933	-2.17858819466208	0.14962949325365
F	-2.91301289308990	-3.41453758934561	0.65725645680055
C	-1.86716666873124	1.64658621008947	0.56026492802065
C	-3.00598616575575	2.33392586606834	-0.19056464548141
F	-3.80744190407385	1.40700512168391	-0.72937256470460
F	-2.46469205293931	0.97557869623780	1.58385907147992
F	-1.13003956546469	2.62989339333296	1.14361712874977
F	-2.55985358733874	3.10541692287392	-1.17648920596508
F	-3.76458822142403	3.10396927044019	0.60322816530793
F	-1.23741939536089	-1.61134378965610	1.80549126625374
F	-0.49335657213874	-2.54379193412096	-0.02937350217281
F	-2.93481363400126	-2.27782505784267	-1.17320678080589
F	-3.73599344481292	-1.43036082357292	0.63955849655200
C	2.58728730471923	-2.38911003818003	-1.47125151709345
C	3.08318136037342	-2.29468472616894	0.96408967645467
C	3.08997235147974	2.71013163208825	0.39554943409408
C	4.14577195143654	0.94913419152589	1.81752317365891
H	1.13678932179449	1.06119263697079	1.07055915343908
H	1.11237433707445	-0.68501377659766	1.12625207826254
H	2.87978600115720	-3.44427744380570	-1.44536129972547
H	1.50718802539090	-2.35030015909816	-1.34160481230861
H	2.81719364705240	-1.99877789759482	-2.46349446032502
H	2.02260971065303	-2.44810128020980	1.15678335645954
H	3.54802681475933	-3.28610569964962	0.96200894239617
H	3.50157804496003	-1.73902938152985	1.80266982363040
H	5.44848438882376	-1.29366345378354	0.09805701863040
H	5.11342064494735	-2.74088502855818	-0.83852706893039
H	5.07521708819387	-1.16399011845758	-1.62458369371271
H	2.22700438321786	2.71684224406883	1.05953650245913
H	3.77143775081048	3.49355451775339	0.74120781709124
H	2.72212470316236	2.98257593453629	-0.59386697515536
H	4.77453703937351	0.05820455790140	1.85537960010128
H	4.68315243128291	1.74779162748394	2.34031395710102
H	3.23553938938087	0.73959910924592	2.38137221755686
H	5.77478629770785	0.70262982973821	-0.38727286540865
H	4.92180406936568	1.82502740803351	-1.44243800784043
H	5.69433894487121	2.40377744770083	0.03541217626914
F	0.19614245640286	2.16015072868590	-1.24140109017290

Point Group: C_1, Symmetry Number: 1

0: 0.00 cm**-1
1: 0.00 cm**-1
2: 0.00 cm**-1
3: 0.00 cm**-1
4: 0.00 cm**-1
5: 0.00 cm**-1
6: 13.78 cm**-1
7: 20.29 cm**-1
8: 31.23 cm**-1
9: 37.69 cm**-1
10: 40.26 cm**-1
11: 50.06 cm**-1
12: 54.43 cm**-1

References

1 J. L. Beckmann, J. Krieft, Y. V. Vishnevskiy, B. Neumann, H.-G. Stammler and N. W. Mitzel, *Angew. Chem. Int. Ed.*, 2023, **62**, e202310439.

2 F. Eisenträger, A. Göthlich, I. Gruber, H. Heiss, C. A. Kiener, C. Krüger, J. U. Notheis, F. Rominger, G. Scherhag and M. Schultz, *New J. Chem.*, 2003, **27**, 540.

3 D.R. Williamson and M. C. Baird, *J. Inorg. Nucl. Chem.*, 1972, **34**, 3393–3400.

4 M. A. Beckett, G. C. Strickland, J. R. Holland and K. S. Varma, *Polymer*, 1996, **37**, 4629–4631; b) U. Mayer, V. Gutmann, and W. Gerger, *Monatsh. Chem.*, 1975, **106**, 1235–1257.

5 J. Ramler and C. Lichtenberg, *Chem. – Eur. J.*, 2020, **26**, 10250–10258.

6 O. V. Dolomanov, L. J. Bourhis, R. J. Gildea, J. A. K. Howard and H. Puschmann, *J. Appl. Crystallogr.*, 2009, **42**, 339–341.

7 G. M. Sheldrick, *Acta Crystallogr.*, *Sect. A*, 2015, **71**, 3–8.

8 G. M. *Sheldrick, Acta Crystallogr.*, *Sect. C*, 2015, **71**, 3–8.

9 L. J. Bourhis, O. V. Dolomanov, R. J. Gildea, J. A. K. Howard and H. Puschmann, *Acta Crystallogr*., 2015, **A71**, 59–75.

10 F. Kleemiss, O. V. Dolomanov, M. Bodensteiner, N. Peyerimhoff, M. Midgley, L. J. Bourhis, A. Genoni, L. A. Malaspina, D. Jayatilaka, J. L. Spencer, F. White, B. Grundkötter-Stock, S. Steinhauer, D. Lentz, H. Puschmann and S. Grabowsky, *Chem. Sci.*, 2021, **12**, 1675–1692.

11 G. Schaftenaar and J. H. Noordik, *J. Comput.-Aided Mol. Des.*, 2000, **14**, 123–134.

12 G. Schaftenaar, E. Vlieg and G. Vriend, *J. Comput.-Aided Mol. Des.*, 2017, **31**, 789–800.

13 S. Grimme, C. Bannwarth and P. Shushkov, *J. Chem. Theory Comput.*, 2017, **13**, 1989–2009.

14 C. Bannwarth, S. Ehlert and S. Grimme, *J. Chem. Theory Comput.*, 2019, **15**, 1652–1671.

15 C. Bannwarth, E. Caldeweyher, S. Ehlert, A. Hansen, P. Pracht, J. Seibert, S. Spicher and S. Grimme, *Wiley Interdiscip. Rev.: Comput. Mol. Sci.*, 2021, **11**, e1493.

16 S. Grimme, *J. Chem. Theory Comput.*, 2019, **15**, 2847–2862.

17 P. Pracht, F. Bohle and S. Grimme, *Phys. Chem. Chem. Phys.*, 2020, **22**, 7169–7192.

18 S. Grimme, A. Hansen, S. Ehlert and J.-M. Mewes, *J. Chem. Phys.*, 2021, **154**, 064103.

19 J. W. Furness, A. D. Kaplan, J. Ning, J. P. Perdew and J. Sun, *J. Phys. Chem. Lett.*, 2020, **11**, 8208–8215.

20 J. W. Furness, A. D. Kaplan, J. Ning, J. P. Perdew and J. Sun, *J. Phys. Chem. Lett.*, 2020, **11**, 9248.

21 S. Ehlert, U. Huniar, J. Ning, J. W. Furness, J. Sun, A. D. Kaplan, J. P. Perdew and J. G. Brandenburg, *J. Chem. Phys.*, 2021, **154**, 061101.

22 H. Kruse and S. Grimme, *J. Chem. Phys.*, 2012, **136**, 154101.

23 B. Metz, H. Stoll and M. Dolg, *J. Chem. Phys.*, 2000, **113**, 2563–2569.

24 F. Neese, *Wiley Interdiscip. Rev. Comput. Mol. Sci.*, 2012, **2**, 73–78.

25 F. Neese, *Wiley Interdiscip. Rev. Comput. Mol. Sci.*, 2017, **8**, e1327.

26 F. Neese, F. Wennmohs, U. Becker and C. Riplinger, *J. Chem. Phys.*, 2020, **152**, 224108.

27 F. Neese, *Wiley Interdiscip. Rev.: Comput. Mol. Sci.*, 2022, **12**, e1606.

28 G. Herzberg, *Molecular Spectra and Molecular Structure*. *II. Infrared and Raman Spectra of Polyatomic Molecules*, Van Nostrand Comp., Toronto, 1945.

29 S. Grimme, *Chem. Eur. J.*, 2012, **18**, 9955–9964.

30 C. Adamo and V. Barone, *J. Chem. Phys.*, 1999, **110**, 6158–6170.

31 S. Grimme, S. Ehrlich and L. Goerigk, *J. Comput. Chem.*, 2011, **32**, 1456–1465.

32 F. Weigend and R. Ahlrichs, *Phys. Chem. Chem. Phys.*, 2005, **7**, 3297–3305.

33 F. Neese, F. Wennmohs, A. Hansen and U. Becker, *Chem. Phys.* 2009, **356**, 98–109.

34 M. J. Frisch, G. W. Trucks, H. B. Schlegel, G. E. Scuseria, M. A. Robb, J. R. Cheeseman, G. Scalmani, V. Barone, G. A. Petersson, H. Nakatsuji, X. Li, M. Caricato, A. V. Marenich, J. Bloino,B. G. Janesko, R. Gomperts, B. Mennucci, H. P. Hratchian, J. V. Ortiz, A. F. Izmaylov, J. L. Sonnenberg, D. Williams-Young, F. Ding, F. Lipparini, F. Egidi, J. Goings, B. Peng, A. Petrone, T. Henderson, D. Ranasinghe, V. G. Zakrzewski, J. Gao, N. Rega, G. Zheng, W. Liang, M. Hada, M. Ehara, K. Toyota, R. Fukuda, J. Hasegawa, M. Ishida, T. Nakajima, Y. Honda, O. Kitao, H. Nakai, T. Vreven, K. Throssell, J. A. Montgomery Jr., J. E. Peralta, F. Ogliaro, M. J. Bearpark, J. J. Heyd, E. N. Brothers, K. N. Kudin, V. N. Staroverov, T. A. Keith, R. Kobayashi, J. Normand, K. Raghavachari, A. P. Rendell, J. C. Burant, S. S. Iyengar, J. Tomasi, M. Cossi, J. M. Millam, M. Klene, C. Adamo, R. Cammi, J. W. Ochterski, R. L. Martin, K. Morokuma, O. Farkas, J. B. Foresman and D. J. Fox, *Gaussian 16 Revision C.01*, Gaussian Inc. Wallingford CT, 2016.

35 K. A. Todd, *AIMAll Version 19.10.12*, TK Gristmill Software, aim.tkgristmill.com, 2019.

36 R. F. W. Bader, *Atoms in Molecules - A Quantum Theory*, Oxford University Press, Oxford, 1990.

37 M. A. Blanco, A. Martín Pendás and E. Francisco, *J. Chem. Theory Comput.,* 2005, **1**, 1096–1109.

38 P. Erdmann, J. Leitner, J. Schwarz and L. Greb, *ChemPhysChem*, 2020, **21**, 987–994.

39 S. Grimme, J. G. Brandenburg, C. Bannwarth and A. Hansen, *J. Chem. Phys.*, 2015, **143**, 54107.

40 S. Grimme, J. Antony, S. Ehrlich and H. Krieg, *J. Chem. Phys.*, 2010, **132**, 154104.

41 F. Weigend, *Phys. Chem. Chem. Phys.*, 2006, **8**, 1057–1065.

42 S. Kozuch, D. Gruzman and J. M. L. Martin, *J. Phys. Chem. C*, 2010, **114**, 20801–20808.

43 A. Hellweg, C. Hättig, S. Höfener and W. Klopper, *Theor. Chem. Acc.*, 2007, **117**, 587–597.
